# Role of Molecular Topology Elucidated in Unified Gels

**DOI:** 10.1021/jacs.6c01062

**Published:** 2026-05-07

**Authors:** Tianjing Luo, Yulin Deng, Mingda Hu, Samuel Kin-Man Lai, Linghui Kong, Zhisheng Wang, Dongwei Zhang, Yiting Liu, Jiajing He, Hang Zhou, Keying Guan, Yijiang Mu, Ho Yu Au-Yeung, Yufeng Wang

**Affiliations:** † Department of Chemistry, 25809 The University of Hong Kong, Pokfulam Road, Hong Kong, SAR 999077, P. R. China; ‡ Chemistry and Chemical Engineering of Guangdong Laboratory, Shantou 515031, P.R. China; § HKU-CAS Joint Laboratory on New Materials, The University of Hong Kong, Pokfulam Road, Hong Kong, SAR 999077, P. R. China; ∥ State Key Laboratory of Synthetic Chemistry, The University of Hong Kong, Pokfulam Road, Hong Kong, SAR 999077, P. R. China

## Abstract

Incorporating topologically nontrivial molecules (e.g., catenanes) is an emerging strategy for tuning properties of polymer networks, but the fundamental roles of these molecules remain poorly understood. Herein, we report a unified gel system employing isomeric linkers with distinct topologies, which allows for cross-gel comparison and elucidation of how topology governs mechanical and dynamic properties in polymeric materials. Our gels feature a panel of phenanthroline-based macrocyclic molecules as linkers, which adopt structures including a large macrocycle, a flexible catenane, and, through metal coordination, a rigidified catenane and a twisted figure-eight structure. By mechanical and thermodynamic analysis and simulations, we identify topology-dependent coordination and conformational entropy as the key factors driving the gels’ mechanical responses. We reveal that the toughness and energy dissipation capacity of the gels correlate directly to the conformational change allowed by each topology, proportional to the “hidden length” that can be released by the linkers. Surprisingly, metal–ligand bonds primarily tune the initial linker conformation rather than dissipate energy through bond dissociation, while they simultaneously enhance network dynamics. Our findings could guide the selection of topological molecules for engineering advanced polymer materials.

## Graphical abstract

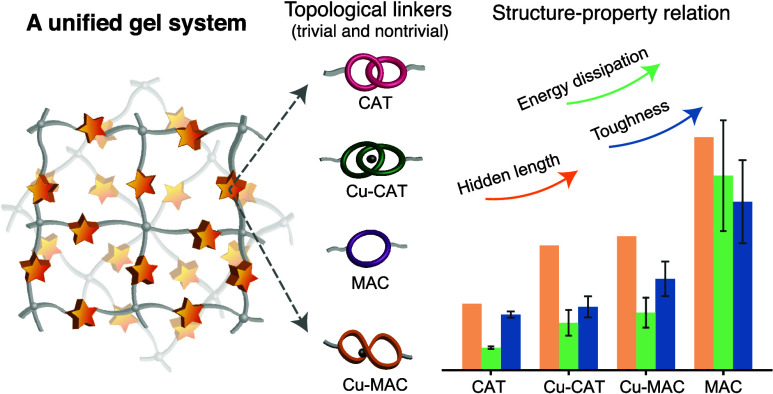

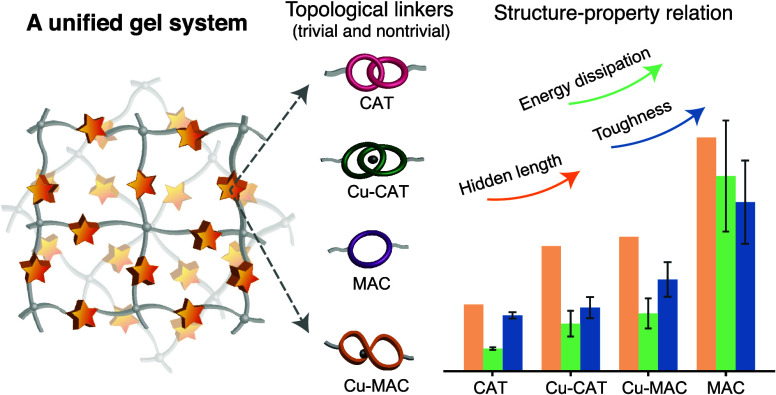

## Introduction

Strategies to tailor key mechanical properties of polymeric materials, such as strength, energy dissipation, and dynamics, are central to developing advanced polymers and soft materials for applications in tissue engineering, soft robotics, and wearable electronics.^1–11^ In this context, supramolecular chemistry offers a powerful toolset by leveraging noncovalent interactions (e.g., hydrogen bonding and metal coordination)^12–19^ and host–guest complexation^20–22^ to endow a delicate control of toughness, self-healing ability, and responsiveness, establishing supramolecular polymers and gels as a prominent class of functional materials.^23–25^

An emerging frontier in supramolecular polymers is the incorporation of topologically nontrivial (trivial) structures (e.g., catenanes^26–28^ and rotaxanes^29–31^ ) and topologically simple yet architecturally complex macrocycles^28,32–35^ in the polymers. Notably, these molecular linkers are characterized by their (co)­conformational freedom and the possession of mechanical bonds. For example, catenanes are a class of mechanically interlocked molecules (MIMs) in which the interlocked rings can slide, flip, and elongate without covalent restriction, whereas flexible macrocycles are foldable and extensible via adopting numerous conformations.^36,37^ These behaviors resemble those of fundamental polymer structures such as chain entanglements and loops, which have long been known to affect the mechanical performance of polymers but are often ill-defined and challenging to control.^38–40^ The integration of molecular linkers with a defined topology is thus a promising approach to elucidate relationships underlying mechanical properties and molecular structures, thereby providing guiding principles for designing advanced polymers.

Various studies have demonstrated the incorporation of catenanes into organic polymers, with recent efforts focusing on how the unique topology of these interlocked structures influences the mechanical properties of the resulting polymeric materials. For example, a study from De Bo has shown that tensional forces applied on a polymer can be efficiently diverted via the (co)­conformational motions of a catenane.^41^ On the other hand, Yan and coworkers have demonstrated that the intercomponent interactions involving interlocked units can regulate the dynamic responses and toughness of polymer networks.^33,42–46^ Independent works from the groups of Rowan et al. and Barnes et al. have also shown that controlling the flexibility of catenane units via host–guest binding can be exploited for tuning the mechanical properties of polymers.^26,28^

Despite these recent advances, a fundamental understanding of how the topology of different molecular linkers dictates the mechanical and dynamic properties of polymers remains elusive. In particular, current studies usually involve molecular linkers of different sizes, topologies, and chemical compositions in disparate polymer systems, rendering a direct comparison and systematic elucidation of the specific roles of the linker topology extremely challenging.^27,34,42,47,48^ Moreover, the structural variations of linkers are often coupled with different noncovalent interactions, such as metal–ligand coordination, which influence the mechanical responses of the materials. As such, we envision that constructing polymer networks within a unified system, where the linkers are chemically highly similar and differ only in their topology, is essential for direct and parallel comparisons, as well as guiding the design of polymer materials with unique characteristics from molecular topology.

Here, we report a unified gel system featuring a panel of molecular linkers derived from phenanthroline-based ligands, which share identical (or highly similar) compositions but adopt distinct structures, including a mechanically interlocked catenane or a topologically trivial macrocyclic architecture, as well as their Cu­(I)-coordinated, rigidified counterparts. Through a combination of mechanical testing, thermodynamic analysis, and molecular simulations, we have identified topology-dependent coordination and conformational entropy as two key factors that drive the gels’ mechanical responses. We reveal that the energy dissipation capacity, as well as the toughness of the gels, correlates directly to a “hidden length” that can be released from each linker, manifested as the maximum conformational change allowed by each structure. We also discover that metal–ligand bonds can enhance the network dynamics while simultaneously regulating energy dissipation. Surprisingly, they primarily tune the initial linker conformation and thus its hidden length rather than adsorb energy through bond dissociation. This work offers new insights for engineering supramolecular materials with tunable mechanical properties originating from molecular topology.

## Methods

### Gel Preparation

Tetra-arm polyethylene glycol (10 kDa) end-capped with dibenzocyclooctyne (tetra PEG-DBCO) was dissolved in acetonitrile to prepare a 15 mM stock solution. To make gels, 133 μL of this stock solution (containing 20 mg of polymer) was charged into a vial, to which a stoichiometric amount of linker (e.g., 4.9 mg of MAC or CAT, 5.7 mg of Cu-MAC or Cu-CAT, and 2.4 mg for the linear control) dissolved separately in 133 μL of solvent (dichloromethane: methanol = 4:1) was added. The mixture was quickly transferred into a Teflon mold, which was sealed by binder clips, curing the sample at room temperature for 24 h. The mold was then opened to allow the solvent to evaporate for 12 h. The dry network was subsequently swollen in propylene carbonate (PC) for 12 h at a fixed polymer fraction (1.2 mg of polymer/1 μL of PC). The PC-swollen gels were directly subjected to mechanical tests. Gels made with different linkers are swollen to comparable ratios. The Teflon molds have a dog-bone shape (for tensile tests) with dimensions of 12 × 2 × 5 mm (excluding the larger terminal parts) or a cylindrical shape (for rheometry) with a 10 mm radius and 5 mm height. Gels are typically made and tested in triplicate.

### Tensile Tests

Tensile tests were conducted using an MTS CMT2103 universal testing machine. Stress–strain curves were obtained with tensile speeds of 100 mm/min. Cyclic tensile tests were performed at strain rates of 100, 150, and 200 mm/min. The Young’s modulus was determined from the slope of the initial linear region (20% strain) of the stress–strain curve. Toughness was obtained by calculating the area under the stress–strain curve through trapezoidal integration. The energy dissipation capacity was obtained from the enclosed area of the cyclic tensile loops. Residual strain refers to the strain when the stress returns to zero during an unloading process. To accommodate experimental errors, the residual strain was obtained by subtracting the initial strain during loading from the strain at zero stress during unloading. Where appropriate, each measurement was performed three times (either on the same sample, different samples, or both), and the average values with error were reported.

### Rheometry

Oscillatory rheological experiments were conducted by using an Anton Paar MCR 302e stress-controlled rheometer. A parallel plate geometry 8.0 mm in diameter was used. The frequency sweep was performed at 25 °C, with 1% strain, over an angular frequency range of 0.01–1000 rad/s. For the three-stage time sweep measurement, the gels were sheared for three consecutive stages: 1% strain and 1 rad/s for 5 min, then at 1000% strain and 1 rad/s for another 5 min, and finally returning to 1% strain and 1 rad/s for an additional 5 min. Stress relaxation experiments were performed at 10% strain and 1 rad/s, at 25 °C. The relaxation profiles were normalized and fitted to a stretched exponential function, $G^{\prime }\left(t\right)/G_{0}^{\prime }=e^{{\left(-t/\tau \right)}^{\alpha }}$ , from which a characteristic relaxation time τ was obtained. The amplitude sweep was performed at 25 °C, with a strain range of 0.1–1000%, at a fixed angular frequency of 1 rad/s. The loss modulus (*G"*) in the amplitude sweep was first normalized by its value at 1% strain and then fitted with a normal distribution to calculate the total energy dissipated per unit volume. Temperature sweep experiments were conducted using 1% strain and 1 rad/s, with temperature cycled between 25 and 90 °C. The loss factor was calculated as tan δ = *G"*/*G'*.

## Results and Discussion

### A Unified Gel System with Directly Comparable Topological Linkers

To promote a direct comparison across molecular topologies and mechanical properties, modular networks containing the same polymer scaffold and isomeric linkers in different structures were constructed. In this case, a model A_4_+B_2_ network is constructed, whereby tetra-arm poly­(ethylene glycol) (PEG) end-capped with dibenzocyclooctyne (tetra PEG-DBCO) (A_4_) forms gels with stoichiometric azide-modified linkers (B_2_) via strain-promoted azide–alkyne cycloaddition (SPAAC) (Figure 1a). In this case, PEG with a molecular weight of 10 kDa (or 2.5 kDa per arm) was chosen. Gels were made at 7.5 mM, slightly above the overlap concentration of the polymer (6 mM), to avoid sol–gel separation while reducing chain entanglement and potential topological defects.

**Figure 1:**
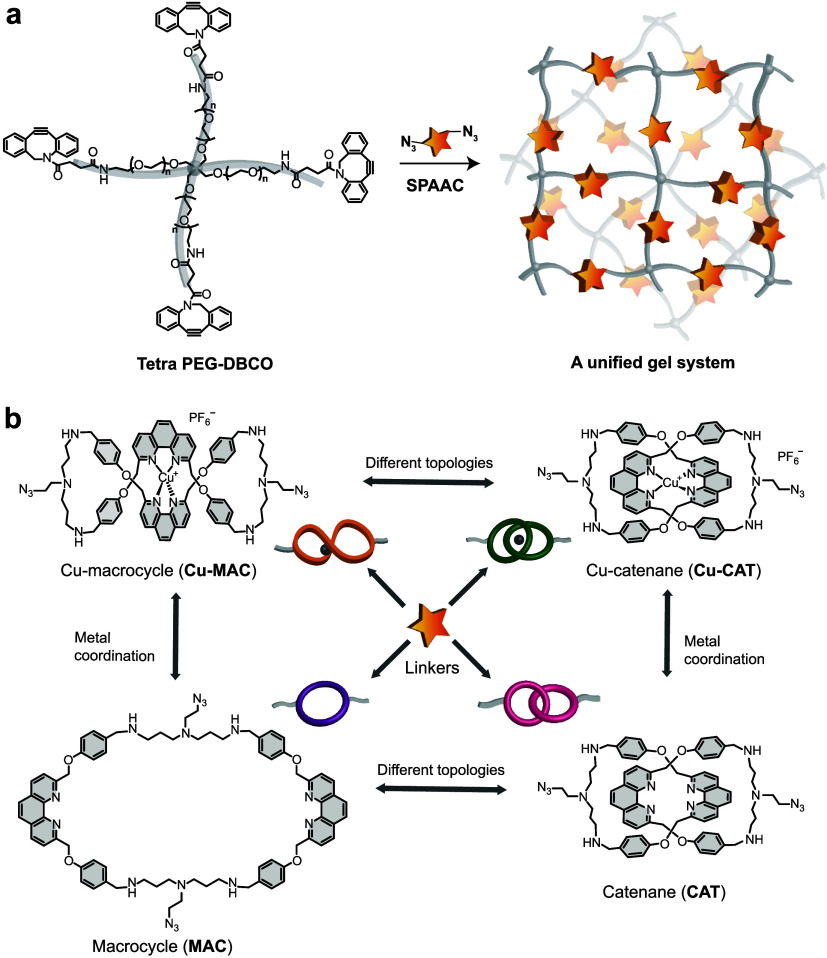
A unified gel system. (a) Schematic for the gel network and its formation. (b) Cartoon and molecular structures of phenanthroline-based topological molecules employed as elastically active linkers of the network.

The core of our design lies in a panel of four phenanthroline-based linker molecules with the same chemical composition but distinct topologies, including two isomeric Cu­(I) complexes, [Cu­(MAC)]­(PF_6_) (or Cu-MAC) and [Cu­(CAT)]­(PF_6_) (or Cu-CAT), along with their metal-free ligands, MAC and CAT (Figure 1b). The synthesis of these linkers is detailed in the Supporting Information, with their structures fully characterized.

For Cu-CAT, the two phenanthroline-based macrocycles are mechanically interlocked as a [2]­catenane, whereas Cu-MAC is a covalently linked bis­(phenanthroline) macrocyclic ligand that folds into a figure-eight conformation due to the tetrahedral coordination geometry of Cu­(I). Folding of Cu-MAC into a chiral figure-eight is confirmed by the diastereotopic splitting of the −OCH_2_– protons in the ^1^H NMR spectrum, which contrasts with the achiral structure of Cu-CAT (Figure 2a, see Figure S1 for full peak assignment). Moreover, 2D DOSY NMR studies (Figure S2) showed that Cu-CAT (*D* = 2.02 × 10^–6^ cm^2^/s) features a higher diffusion coefficient than Cu-MAC (*D* = 1.83 × 10^–6^ cm^2^/s), suggesting that the mechanical interlocking resulted in an overall more compact structure of the catenane complex as compared to the molecular folding in the figure-eight complex. The more compact structure of Cu-CAT than that of Cu-MAC is also supported by the more upfield-shifted phenyl protons (Figure 2a, blue-colored peaks) due to the reinforced π-stacking with the phenanthroline. On the other hand, the identical chemical composition of Cu-MAC and Cu-CAT is evident from the mass spectrometry data (Figure 2a). As for the metal-free ligands, MAC is a flexible macrocycle with a relatively large molecular dimension and the smallest diffusion coefficient among the four linkers (*D* = 1.59 × 10^–6^ cm^2^/s), whereas CAT is structurally compact (*D* = 1.85 × 10^–6^ cm^2^/s) but also conformationally flexible. The mechanical interlocking in CAT also resulted in an overall stronger shielding effect, and the phenyl proton signals were hence relatively more upfield-shifted than those in MAC (Figure 2b). It is worth noting that MAC and CAT are also isomeric with an identical chemical composition, as shown by the mass spectra in Figure 2b.

**Figure 2:**
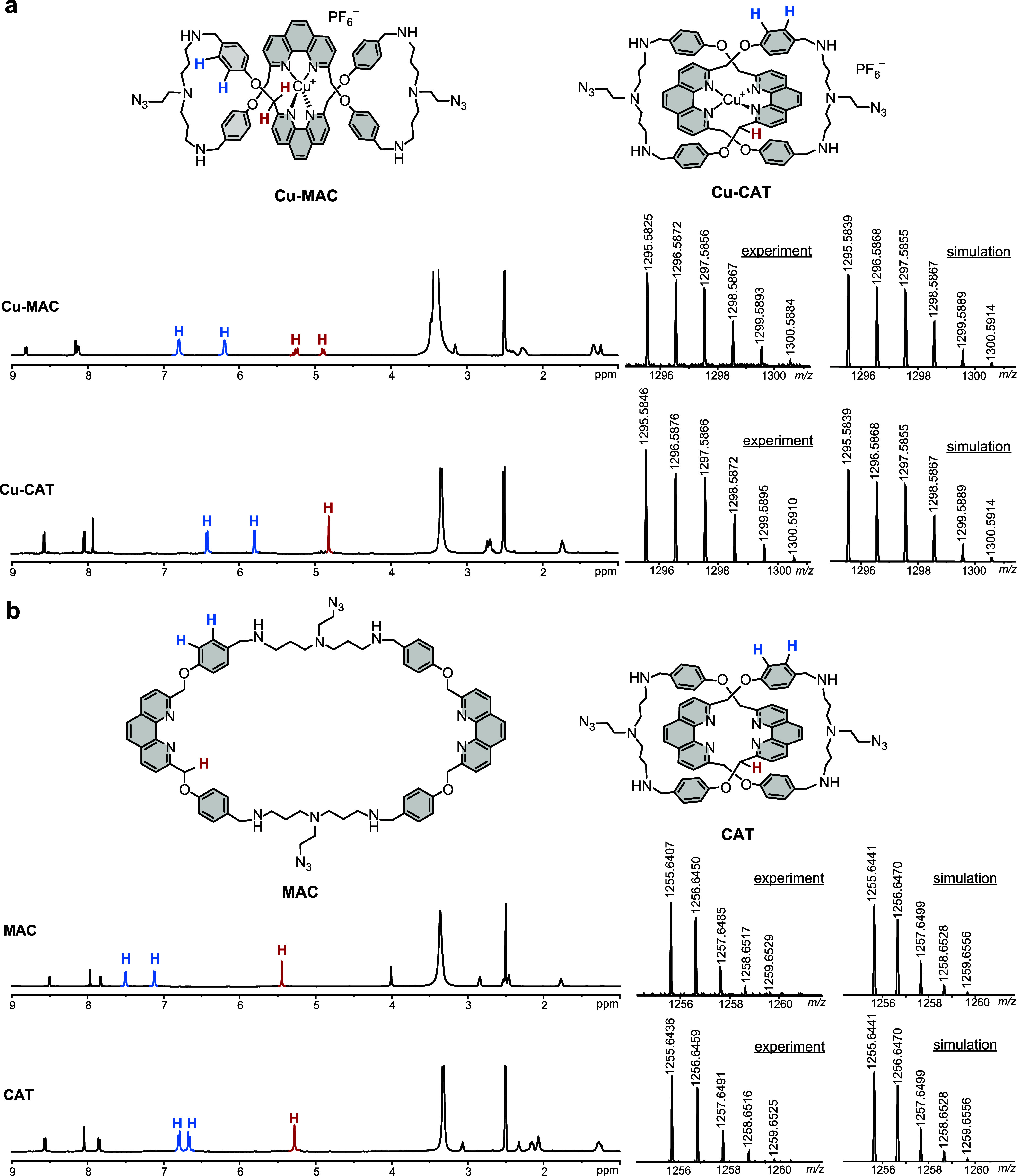
Structure and characterization of the linkers. ^1^H NMR (500 MHz, DMSO-*d*_6_, 298 K) and mass spectra of (a) Cu-MAC, Cu-CAT, and (b) MAC and CAT, respectively. The mass spectra confirm the formation of [Cu-MAC]^+^, [Cu-CAT]^+^, [MAC+Na]^+^, and [CAT+Na]^+^.

Each of these linkers is equipped with two azide groups on the opposite sides of the molecules, which are chemically incorporated into the tetra-arm PEG network as elastically active linkers via SPAAC. The SPAAC reaction is effective, reaching a high conversion of >95% within such gels, as suggested by FT-IR (Figure S3) and previous literature.^49^ Four distinct gels were thus formed, creating a two-dimensional comparison framework based on the phenanthroline ligand topology (macrocycle vs catenane) and their metal-coordination state (with vs without Cu^+^). In addition, a gel linked by a linear control (LC) molecule with similar chemical composition (i.e., the phenanthroline) was also prepared to verify the model network design and to distinguish the role of the molecular topology (Figure S4).

### Mechanical Properties of Cu-MAC and Cu-CAT Gels

The mechanical performance of the Cu-MAC and Cu-CAT gels was first evaluated. Tensile tests revealed that the Cu-MAC gel possesses a slightly lower Young’s modulus (*E* = 3.8 ± 0.7 kPa) but a higher breaking strain (341 ± 51.7%), compared to 3.9 ± 0.3 kPa and 264 ± 8.7% for the Cu-CAT gel (Figure 3a). The Cu-MAC gel also outperforms the Cu-CAT gel with a significantly higher tensile strength (567 ± 73.2 vs 495 ± 143.0 kPa). The combination of these properties results in a higher toughness for the Cu-MAC gel (1099 ± 253.7 kJ/m^3^ vs 762 ± 154.6 kJ/m^3^ for the Cu-CAT gel), indicating a superior capacity for energy absorption.

**Figure 3:**
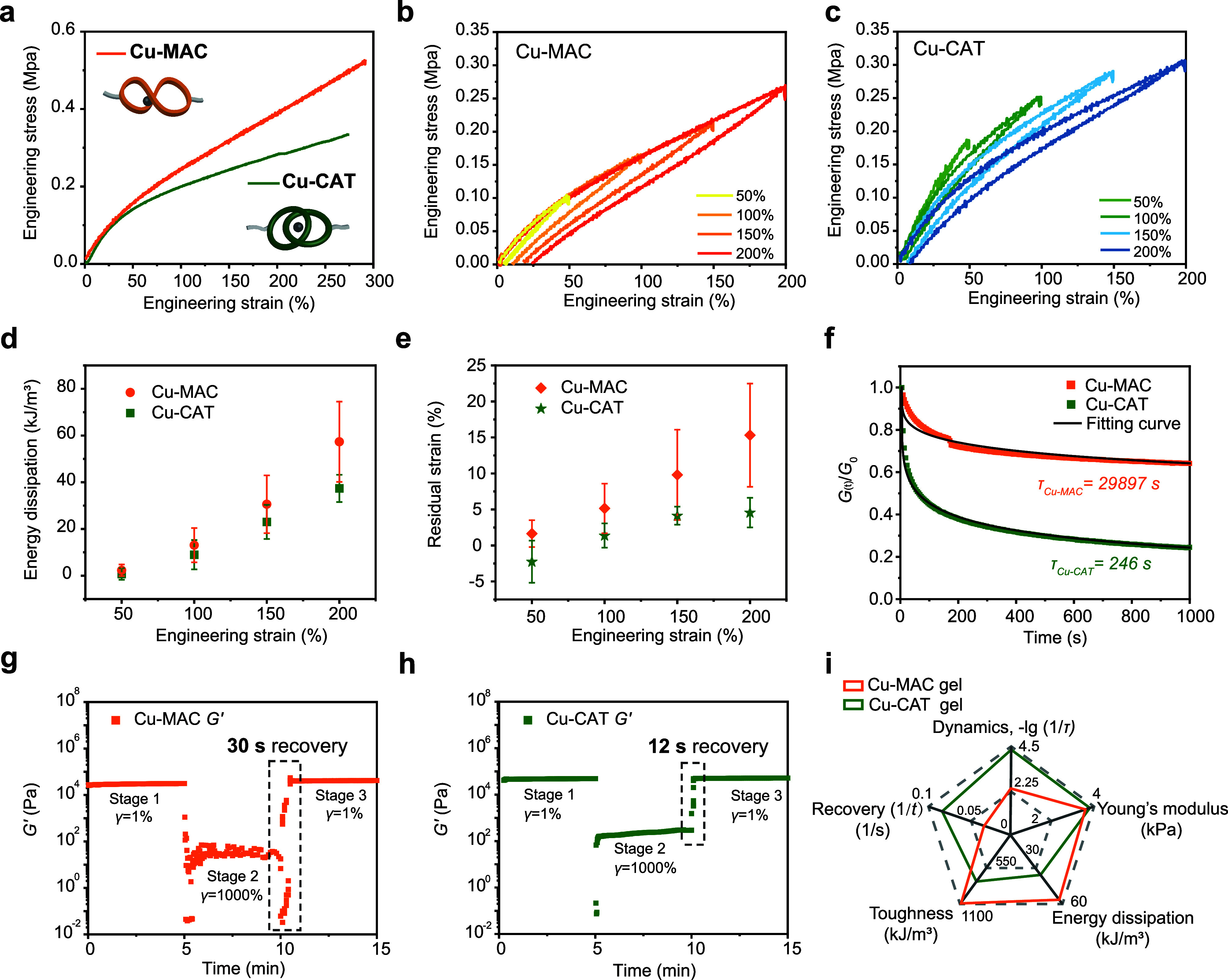
Mechanical properties of Cu-MAC and Cu-CAT gels. (a) Stress–strain curves of Cu-MAC and Cu-CAT gels (strain rate: 100 mm/min). Cyclic tensile testing of (b) Cu-MAC and (c) Cu-CAT gels under different strains (50–200%) (strain rate: 100 mm/min). (d) Energy dissipation and (e) residual strain corresponding to different strains. (f) Normalized stress relaxation of Cu-MAC and Cu-CAT gels under a shear strain of 10% at 25 °C. Three-stage rheological time sweeps for (g) Cu-MAC and (h) Cu-CAT gels. (i) Radar chart summarizes and compares the mechanical properties of Cu-CAT and Cu-MAC gels.

Cyclic tensile tests were subsequently conducted at varying strain levels to characterize the energy dissipation capacity (Figure 3b,c). Both the Cu-CAT and Cu-MAC gels exhibited apparent hysteresis loops, the size of which grew with increasing strain, demonstrating their ability to dissipate energy. The Cu-MAC gel performs better than the Cu-CAT gel in this regard across all strain levels, consistent with its greater intrinsic toughness. For example, at 200% strain, the dissipated energy for the Cu-MAC gel (57.3 ± 17.2 kJ/m^3^) is 1.5 times higher than that of the Cu-CAT gel (37.4 ± 5.8 kJ/m^3^, Figure 3d). While the energy dissipation capacity also depends on the strain rate, the trend applies to both gels (Figures S5 and S6). Following each strain cycle, both gels exhibited a residual strain, ranging from −2.3 ± 2.9% to 15.3 ± 7.2%, proportional to the applied strain (Figure 3e). The Cu-CAT gel exhibited consistently smaller residual strain in comparison to the Cu-MAC gel. The relatively more rapid recovery of the Cu-CAT gel suggests faster elastic recovery kinetics.

The gels were also investigated by oscillatory rheometry. The linear viscoelastic window (LVE) of the gels was first determined (Figures S7 and S8). Stress relaxation analysis was conducted near the LVE regime. The results showed faster decay of the shear storage modulus (*G'*) for the Cu-CAT gel than for the Cu-MAC gel. The relaxation profiles were normalized and fitted to a stretched exponential function, $G^{\prime }\left(t\right)/G_{0}^{\prime }=e^{{\left(-t/\tau \right)}^{\alpha }}$ , from which a characteristic relaxation time τ was obtained. For the Cu-CAT gel, τ_Cu‑CAT_ ∼ 250 s, indicating faster relaxation compared to that of the Cu-MAC gel, with τ_Cu‑MAC_ ∼ 30,000 s (Figure 3f).

The recovery properties of the Cu-MAC and Cu-CAT gels are further assessed by a three-stage rheological time sweep experiment (Figure 3g, h). Following a 1000% strain that induced network disruption (*G"* (shear loss modulus) > *G'*), the Cu-MAC gel showed a *G'* recovery after 30 s. In contrast, the Cu-CAT gel restored its original modulus (*G'*) within 12 s. The recovery rate of *G"* follows the same trend as *G'*, as shown in Figure S9. Overall, the Cu-CAT and Cu-MAC gels exhibit distinct mechanical properties. While the Cu-MAC gel dissipates more energy and exhibits greater toughness, the Cu-CAT gel recovers more rapidly, displaying a more dynamic behavior and showing a higher Young’s modulus (Figure 3i). These differences stem from the different topologies of the copper­(I) complexes.

### Mechanical Properties of MAC and CAT Gels

Gels containing MAC and CAT as linkers were then investigated. In addition to their distinct topology, although being isomeric, CAT and MAC can also be compared with Cu-CAT and Cu-MAC to understand the role of Cu­(I)-phenanthroline coordination, thus serving as critical controls for isolating the specific contributions of molecular topology and metal–ligand coordination to the mechanical behaviors of the gels.

The tensile profiles of the MAC and CAT gels are shown in Figure 4a. The Young’s moduli are 3.0 ± 0.2 kPa for the MAC gel and 1.0 ± 0.4 kPa for the CAT gel. The MAC gel exhibits high toughness (2,025 ± 210.7 kJ/m^3^), in contrast to the low toughness (669 ± 47 kJ/m^3^) of the CAT gel.

**Figure 4:**
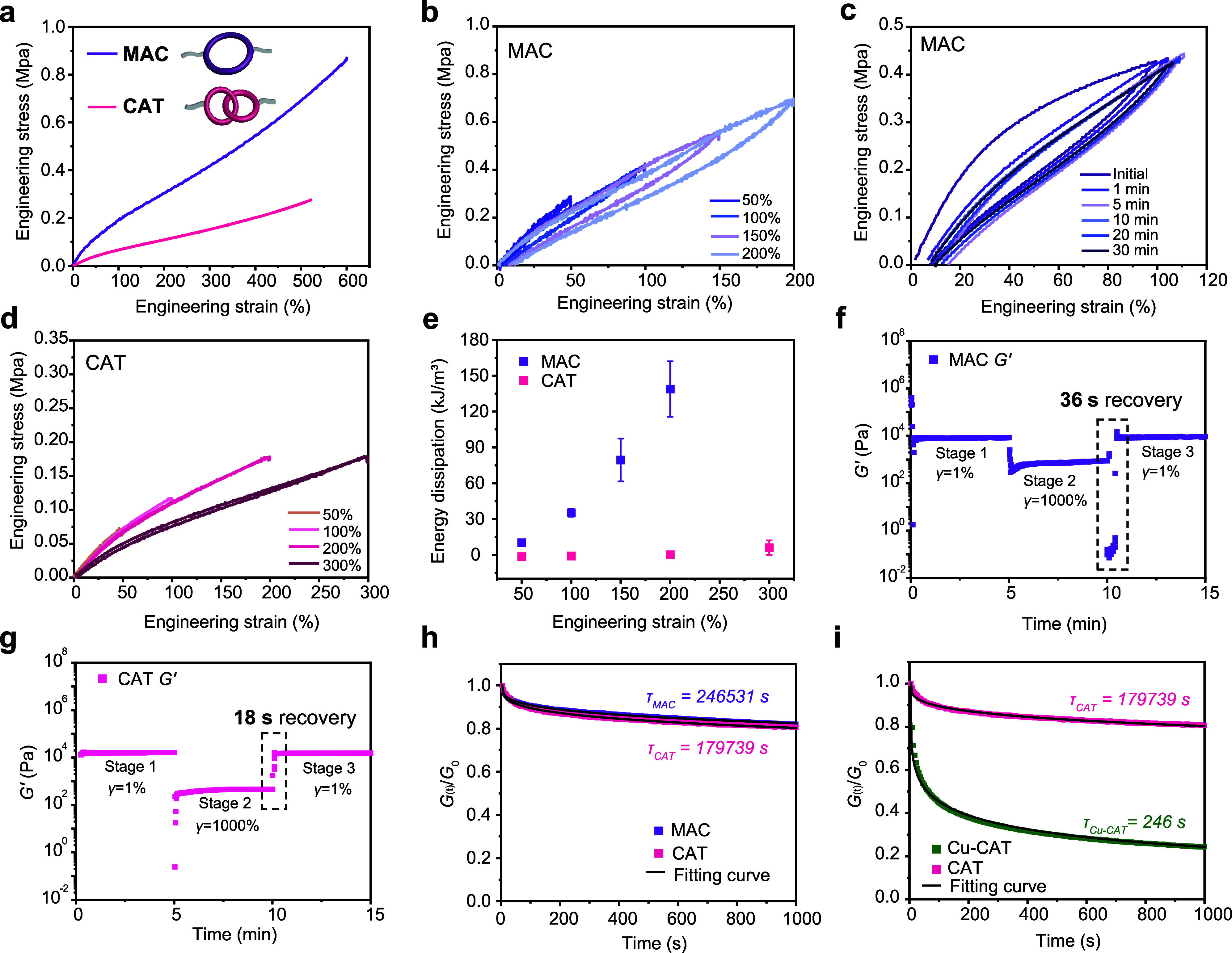
Mechanical properties of MAC and CAT gels. (a), Stress–strain curves of MAC and CAT gels (strain rate: 100 mm/min). (b) Cyclic tensile curves of MAC gel at 50–200% strain. (c) MAC gel with recovery intervals from 0 to 30 min at 100% strain. (d) Cyclic tensile curves of CAT gel at 50–300% strain. (e) Energy dissipation capacity for MAC and CAT gels at different strains. Three-stage rheological time sweeps performed for (f) MAC and (g) CAT gels. (h) Stress relaxation profiles of MAC and CAT gels with characteristic relaxation time. (i) Stress relaxation profiles of CAT and Cu-CAT gels comparing their characteristic relaxation time.

In cyclic tensile tests, large hysteresis loops were observed for the MAC gels, both at increasing strains (50–200%, Figure 4b, and Figure S10 for different strain rates) and following various recovery times between loading/unloading cycles (strain = 100%) (Figure 4c). These results correspond to substantial energy dissipation. As the recovery time increases, the area of the hysteresis loop became smaller than the initial value at 1 min of recovery and remained stable across different recovery intervals (Figure 4c). In stark contrast, the cyclic tensile profile of the CAT gels at different strain levels (up to 300%) showed negligible energy dissipation (Figure 4d). The gel behaved nearly elastically, with minimal residual strain and hysteresis, with merely 6.0 ± 6.1 kJ/m^3^ for the loop area at 300% strain. The gels also show no significant energy dissipation during cyclic stretching at different time intervals (Figure S11). The comparison of energy dissipation for the MAC and CAT gels is shown in Figure 4e.

The recovery properties of the CAT and MAC gels were characterized by rheometry. The *G’* value of the MAC gel recovered after 36 s following a 1000% strain disruption, while *G’* of the CAT gel recovered within 18 s (Figure 4f,g). The MAC and CAT gels showed comparable stress relaxation profiles, with characteristic relaxation times of τ_MAC_ ∼ 250,000 s and τ_CAT_ ∼ 180,000 s, respectively (Figure 4h). Their relaxation is much slower compared to that of the Cu-MAC and Cu-CAT gels (Figures 4h,i and S12).

The LC gels were also investigated in parallel. They show weak mechanical strength and negligible energy dissipation capacity, comparable to the properties of CAT gels (Figure S13). The lack of mechanical features verifies our model system design (i.e., tetra-arm PEG network by SPAAC), where intrinsic network defects such as loops (if any) do not surpass the topological linkers in contributing to the gel properties such as energy dissipation.

To further evaluate their mechanical and dynamic properties, amplitude sweep experiments were conducted for all gel types over a broader amplitude range, from 0.1% to 1000%. As can be observed from Figure 5a,b, at small strain values (<10%), the *G'* and *G"* values for Cu-MAC, Cu-CAT, and CAT gels are relatively stable. As the strain increases (>10%), the gels enter a strain-softening regime, where *G'* values start to decline and the *G"* values increase (for MAC gels, softening starts at an even smaller strain). The *G'* and *G"* values cross at an intermediate strain level, which is also the point when *G"* is maximized. The profiles of *G"* for all gel types are normalized and compared in Figure 5c. By fitting the loss maximum and integrating the area under each peak, we obtained the energy dissipation per unit volume. The results show that the MAC gel has the greatest dissipation, *U* = 91.2 kJ/m^3^, while the CAT gel has the smallest, with *U* = 24.8 kJ/m^3^. The Cu-CAT and Cu-MAC exhibit intermediate dissipation, with *U* = 40.1 kJ/m^3^ and *U* = 44.3 kJ/m^3^ (Figure 5d). We note that the trend for energy dissipation observed herein (rheometry) is consistent with that obtained by the tensile experiments. Furthermore, we noticed that the peaks of *G"* for Cu-CAT and Cu-MAC gels occur at smaller strain values (30–40%) compared to those of CAT and MAC gels (100% for MAC and 200% for CAT). We suspect that this has to do with the presence of metal coordination, which may facilitate gel dynamics, so that energy dissipation occurs at an early stage during sample shearing. Besides amplitude sweep, frequency sweep over a broader range was conducted, with the results shown in Figures S14 and S15. We note that there is no obvious trend (of both *G"* and loss factor) for different gels in the low-to-intermediate frequency range (<100 rad/s), while at higher frequencies, the data is not reliable, presumably due to limitations of the instrument or our gel system.

**Figure 5:**
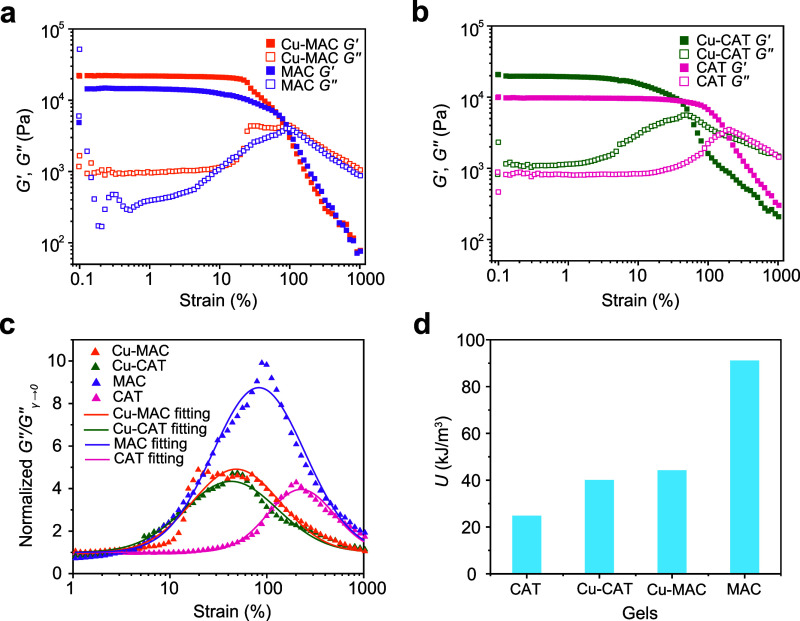
Amplitude sweeps in a broad range. (a, b) Amplitude sweep experiments of MAC, Cu-MAC, CAT, and Cu-CAT gels; the strain range is 0.1–1000%. (c) Normalized *G"* for all gels showing the loss maximum, which is fitted to a normal distribution. (d) Energy dissipation per unit volume *U* obtained by calculating the areas of *G"* profiles in (c).

Several points can be made from the above observations. First, the MAC gel exhibits pronounced energy dissipation, which is presumably due to the rich conformations possessed by the large macrocycle, similar to those containing other cyclic molecules (e.g., crown ether^34^) reported previously. In contrast, the CAT gel, featuring the interlocked catenane linker in an overall smaller ring size, dissipates little energy and displays high elasticity. Although catenanes could undergo (co)­conformational motions through ring-sliding and twisting, these motions do not seem to contribute to energy dissipation. Second, by comparing the CAT and Cu-CAT gels, metal coordination was found to increase both the Young’s modulus as well as the energy dissipation capacity. However, from the MAC gel to the Cu-MAC gel, metal coordination decreases the gel’s ability to dissipate energy. This observation is counterintuitive, as coordination bonds usually absorb energy. Third, the MAC gel shows a slower recovery dynamic, which is consistent with its larger molecular size and higher conformational freedom, whereas the CAT gel exhibits faster kinetics and rapid recovery. The addition of metal ions facilitates such conformational changes, albeit to varying extents, as shown in the stress relaxation experiments. These properties are further discussed below to gain a deeper understanding.

### Thermodynamic Framework and Thermo-Mechanic Properties of Gels

Our results show that the mechanical properties of the gels are dependent on both the linker topology and metal-coordination states. Apart from allowing cross-gel comparison, a unique advantage of our system is that all the linkers can be associated (or unified) by a single ligand-exchange equilibrium (note that *tert*-butyloxycarbonyl (Boc)-protected ligands are used for enhanced solubility) (Figure 6a, see also discussion in SI):

Cu‐(MAC‐Boc)+CAT‐Boc⇌Cu‐(CAT‐Boc)+MAC‐Boc

**Figure 6:**
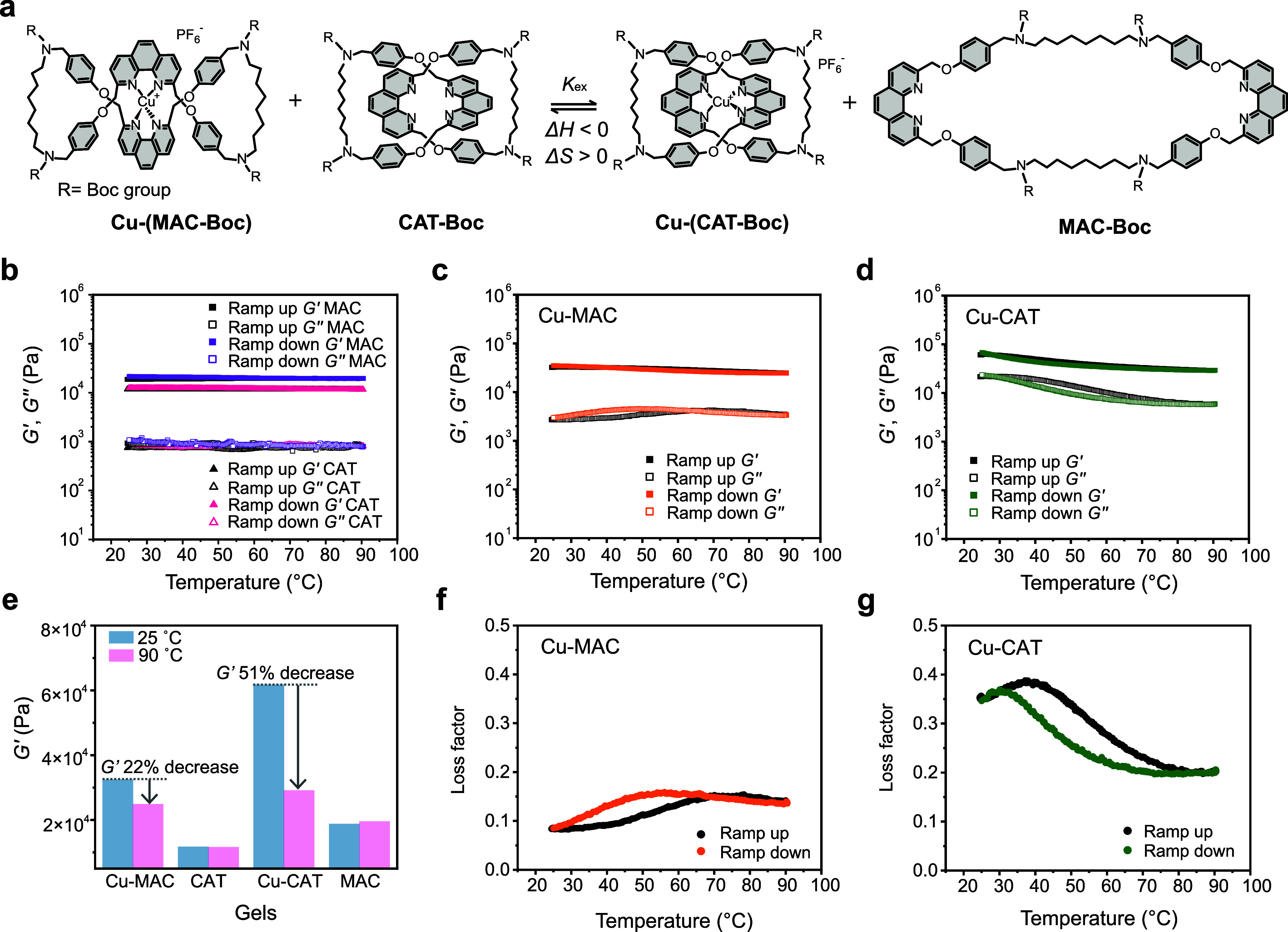
Thermodynamic analysis and thermo-mechanical properties of gels. (a) Ligand exchange equilibrium initiating from Cu-(MAC-Boc) and CAT-Boc (R = Boc). (b–d) Rheometry (*G'*, *G"*) showing a representative temperature cycle of (b) MAC, CAT, (c) Cu-MAC, and (d) Cu-CAT gels. (e) Comparison of *G'* of different gels at 25 and 90 °C during a representative temperature cycle. (f, g) The loss factors of Cu-MAC and Cu-CAT gels in a representative temperature cycle.

By investigating this equilibrium, a thermodynamic framework can be established to further unveil the role of ligand topology underlying the different mechanical properties observed in the gel system. Analysis of an equilibrated ligand mixture by ^1^H NMR spectroscopy yielded an equilibrium constant of *K*_ex_ = 13.7 for the Cu^+^ exchange from MAC-Boc to CAT-Boc (Figure S16). This suggests that the Cu­(I) coordination to the catenane is more stable than that to the topologically trivial macrocycle. Van’t Hoff analysis by variable temperature ^1^H NMR showed that the Cu^+^ exchange from MAC-Boc to CAT-Boc is both enthalpically (Δ*H* = −5.96 ± 0.3 kJ·mol^–1^) and entropically (Δ*S* = 1.16 ± 0.9 J·mol^–1^·K^–1^) favorable (Figure S17). The more favorable enthalpy for Cu^+^ to coordinate to CAT relative to MAC can be explained by enhanced secondary interactions involving the aromatic units and aliphatic linkers in the catenane upon metal coordination, which can also be evidenced from the corresponding ^1^H NMR spectra of the Cu (I) complexes (Figure 2). The more stable Cu^+^ coordination to the catenane is also consistent with the higher (though by a small extent) Young’s modulus of the Cu-CAT gel, which indicates the gel’s resistance to initial deformation. On the other hand, as MAC is less preorganized than CAT, releasing the metal ion from the Cu^+^-complexed figure-eight structure resulted in an increase in the entropy of the system. In principle, the entropy involved can be assigned to the inherent configurational entropy of the macrocycles (i.e., MAC-Boc), and the efficient energy dissipation of the MAC gel can hence be rationalized by the large conformational freedom of MAC.

The above thermodynamic analysis of linkers can be further validated by evaluating the corresponding gels in terms of their thermo-mechanical responses and recovery behaviors. The gels were subjected to successive heating–cooling cycles (25–90 °C) while their viscoelastic properties were monitored via rheometry. Several consecutive cycles were conducted to ensure a clear, stable structure–property relationship could be observed (Figures S18 and S19). Figure 6b–d displays the representative cycles for each gel. Experiments on the metal-free CAT and MAC gels showed an almost perfectly overlapping and featureless modulus profile throughout the heating–cooling cycles, confirming that the observed phenomena are solely due to the interplay of metal coordination and ligand topology (Figure 6b). As the temperature increased from 25 to 90 °C, the *G'* values of both the Cu-containing gels (i.e., Cu-CAT and Cu-MAC gels) decreased (Figure 6c,d), which is attributed to the thermally facilitated dissociation of metal-coordination bonds within the networks. Notably, the extent of gel softening upon heating is highly dependent on ligand topology. From 25 to 90 °C, *G’* of the Cu-MAC gel dropped by only 22%, which is significantly less than the 51% decrease of *G'* observed for the Cu-CAT gel (Figure 6e). This agrees with the fact that the Cu­(I) coordination in Cu-CAT is overall stronger than that in Cu-MAC. Upon cooling, the modulus for both gels recovered, indicating a high degree of reversibility of the metal-coordination bonds.

The loss factor (tan *δ = G"/ G'*) was also derived to suggest energy dissipation of gels at different temperatures (Figure 6f,g). The loss factors stay relatively constant for CAT and MAC gels, showing no temperature dependence (Figure S20), while for the metal-containing Cu-CAT and Cu-MAC gels, the loss factors are strongly dependent on temperature and show hysteresis during heating/cooling cycles. Interestingly, for the Cu-MAC gel, the loss factor increases when temperature raises, while for the Cu-CAT gel, the loss factor decreases despite the initial small increase. We explain the results as follows: at high temperature, the metal coordination will be weakened, so the dissipation ability for Cu-CAT will shift toward that of CAT and thus show a decreased value; the dissipation ability for Cu-MAC will shift toward that of MAC, showing an increased value. This observation aligns with results from tensile tests, where metal coordination increases energy dissipation of CAT (in the case of Cu-CAT) and decreases the energy dissipation of MAC (in the case of Cu-MAC). Moreover, the temperature hysteresis for Cu-CAT and Cu-MAC gels shows opposite profiles (i.e., for Cu-MAC, *G"* measured during cooling is larger than that during heating; for Cu-CAT, *G"* measured during cooling is smaller than that during heating), which can be attributed to the structural differences in the ligands, yet the specific reason remains to be explored.

### Molecular Simulation by DFT

To further understand the relationship between mechanical properties and linker structure, density functional theory (DFT) simulations on models representing CAT, Cu-CAT, Cu-MAC, and MAC were performed. Geometric optimization was first carried out for each of the linkers to obtain their energy-minimized structure. For each model, the two terminal nitrogen atoms of the azide groups were progressively displaced from their equilibrium positions, the structure was reoptimized, and the relative energy compared to the initial structure was computed. This process was repeated until the rupture of the covalent bond occurs, simulating the stretching of the polymer chain under load.

For all linker models, the energy profiles exhibit a flat region before a sharp increase as the displacement from the equilibrium position (DEP) grows (Figure 7a). The width of the flat region varies across different models and can be measured as the maximum displacement from the equilibrium position, or DEP_max_, where the relative energy change remains below 50 kJ/mol (arbitrarily defined). The DEP_max_ represents the “hidden length” that the linker molecule can release when stretched and corresponds to the extent of conformational change that a specific linker can allow.

**Figure 7:**
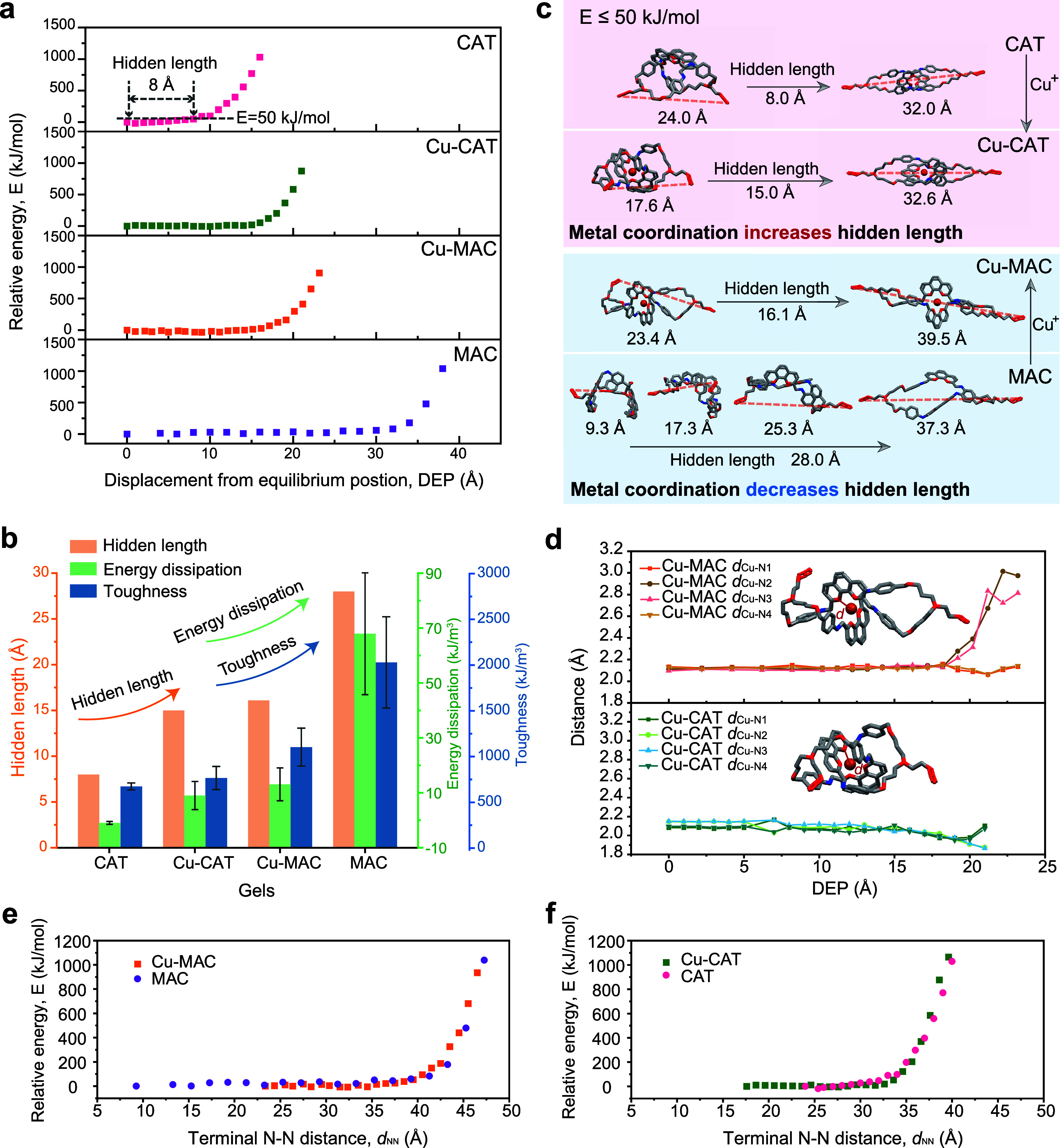
DFT simulation. (a) Relative energies of CAT, Cu-CAT, Cu-MAC, and MAC as a function of displacement from the equilibrium position. A hidden length is defined and measured for each linker. (b) The energy dissipation capacity and toughness of gels are proportional to the hidden length of the linkers. (c) Conformational changes of linkers under load and hidden length released. (d) Distance of Cu and N atoms for Cu-MAC and Cu-CAT. (e, f) Overlay of relative energy versus terminal N–N distance for Cu-MAC versus MAC (e) and for Cu-CAT versus CAT (f).

First, we observed a gradual increase in the hidden length, from CAT, Cu-CAT, Cu-MAC, to MAC. This trend in hidden length positively correlates with the energy dissipation capacity as well as the toughness of the corresponding gels (Figure 7b). The linear control linker has a small hidden length, consistent with its negligible energy dissipation capacity (Figure S21). While the introduction of hidden lengths^2,50–53^ in polymers has been an emerging strategy for tuning mechanical properties, we provide a semiquantitative analysis for this endeavor, connecting specific linker topology to the obtained properties.

Second, to our surprise, the effect of metal coordination on the hidden length differs significantly between the CAT and the MAC systems (Figure 7c). For CAT, an initial distance of 24.0 Å between the two terminal N atoms (*d*_NN_) was found, which can be stretched to 32.0 Å without increasing the overall energy by over 50 kJ/mol, corresponding to a hidden length of 8.0 Å. In Cu-CAT, the metal coordination rigidifies the structure with a shorter initial *d*_NN_ of 17.6 Å. Notably, a similar stretching profile was found for Cu-CAT and CAT, and hence a markedly longer hidden length of 15.0 Å resulted (Figure 7c, top panel). Thus, metal coordination increases the hidden length.

In contrast, MAC is much more flexible than CAT without the restriction from mechanical interlocking and can be folded more efficiently into a boat-shaped conformation, with a much shorter initial *d*_NN_ of 9.3 Å. For Cu-MAC, metal coordination induces a conformational change to an extended figure-eight conformation, significantly increasing the initial *d*_NN_ to 23.4 Å. Albeit their different extended structures, the distance between the two terminal nitrogen atoms in the fully extended state is also similar for MAC and Cu-MAC, and as such, a decrease in the hidden length from 28.0 Å in MAC to 16.1 Å in Cu-MAC results (Figure 7c, bottom panel).

It is worth emphasizing that while metal coordination rigidifies both linkers (CAT and MAC), an opposite effect in tuning the hidden length was observed. This contrast is due to the distinct topology in CAT and MAC that leads to their different conformational flexibilities and equilibrium structures (also Cu-CAT and Cu-MAC). For example, the reduction in hidden length from MAC (28.0 Å) to Cu-MAC (16.1 Å) arises because the Cu­(I)-complexed figure-eight is already in an extended conformation with limited conformational freedom for further elongation (Figure 7c). On the other hand, coconformations with an extended initial *d*_NN_ are only available for the flexible CAT but not the rigidified Cu-CAT, and as both linkers are interlocked with a similar overall structure and stretching profile, there is an increase in the hidden length from CAT (8.0 Å) to Cu-CAT (15.0 Å).

Unlike a previously reported catenane-incorporated polymer system, where the external force is applied directly to the metal-coordination site in the inner loop, our design places the pulling points on the outer loop.^42^ This difference prevents early breaking of the metal-coordination bonds. Analysis of the bond lengths between N atoms of the phenanthroline and Cu^+^ ions in Cu-CAT and Cu-MAC showed that the Cu–N bonds distort/break only after the system has experienced a marked energy increase due to the elongation/rupture of covalent bonds (Figures 7d and S22). In addition, the plot of relative energy versus terminal N–N distance reveals that, in the late stretching stage, the energy curves of Cu-MAC/MAC and Cu-CAT/CAT, respectively, approach near coincidence (Figure 7e,f). This implies that metal coordination does not directly participate in energy absorption. Its main role is to modulate the available hidden length of the topology, thereby regulating the dynamic conformational response and controlling the energy dissipation capacity. We anticipate that tuning the hidden length of an existing topological molecule becomes a facile method for regulating material properties.

## Conclusions

We have designed a unified gel system in which the polymer networks are linked by several macrocyclic molecules with identical/similar composition but diverse topologies. The mechanical and dynamic properties of the gels are thoroughly evaluated, reflecting the structural and topological differences of the linker molecules. Based on thermodynamic analysis as well as DFT simulations, we have reached several key conclusions that relate molecular topology and bonding to the properties of the gels. The CAT gels are elastic, while the MAC possesses significant conformational freedom, endowing the corresponding gel with substantial energy dissipation capacity. We observed a positive correlation between the energy dissipation capacity and toughness of the gels and the hidden length released by the topological molecules under stretching. Metal coordination modulates the hidden length, while simultaneously enhancing the gel dynamics and recovery rate.

Looking forward, our system can be further extended by systematically tuning the size of the macrocycle and catenane linkers. This allows for a precise study of how the molecular cycle size influences key viscoelastic properties, including energy dissipation efficiency and shape-recovery speed. Alternative metal ions like Zn^2+^ or Fe^2+/3+^ could also be explored, as they offer distinct coordination geometries and binding kinetics. The ability to regulate the metal coordination cross-links in polymer networks enables fine-tuning of stiffness and self-healing behaviors. Moreover, these topological molecules can be integrated into other polymer systems with practical applications, such as polyimide, which are commonly used in foldable displays. The understanding of molecular topology could facilitate the engineering of the mechanical resilience and fatigue resistance of materials. These strategies would advance the development of functional polymeric materials with durable and adaptable mechanical properties.

## Supplementary Material



## Notes

Materials and methods; synthetic procedures and characterization data; Supplementary Figures (PDF)
