# Supplementary material for: Role of Molecular Topology Elucidated in Unified Gels
Source: J Am Chem Soc. 2026 May 7;148(19):19700–11. doi: 10.1021/jacs.6c01062 (PMC13195675; doi:10.1021/jacs.6c01062)
Supplement: Supplementary file 1 [file ja6c01062_si_001.pdf]

Supporting Information for

Role of Molecular Topology Elucidated in Unified Gels

Tianjing Luo,[1,2†] Yulin Deng,[1,†] Mingda Hu,[1,†] Samuel Kin-Man Lai,[1] Linghui Kong,[1] Zhisheng Wang,[1] Dongwei Zhang,[1] Yiting Liu,[1] Jiajing He,[1] Hang Zhou,[1] Keying Guan,[1] Yijiang Mu,[1] Ho Yu Au-Yeung,[1,3,4,*] and Yufeng Wang[1,3,4,*]

[1]Department of Chemistry, The University of Hong Kong, Pokfulam Road, Hong Kong SAR, P. R. China, 999077

[2]Chemistry and Chemical Engineering of Guangdong Laboratory, Shantou, P.R. China，515031

[3]HKU-CAS Joint Laboratory on New Materials, The University of Hong Kong, Pokfulam Road, Hong Kong SAR, P. R. China, 999077

[4]State Key Laboratory of Synthetic Chemistry, The University of Hong Kong, Pokfulam Road, Hong Kong SAR, P. R. China, 999077

[†]T.L., Y.D. and M.H. contributed equally.

[*]To whom correspondence should be addressed: hoyuay@hku.hk; wanglab@hku.hk.

Table of Contents

1. Materials and methods 2

2. Molecular synthesis and characterization 5

3. Supplementary figures 13

4. Appendix: ^{1}H and ^{13}C NMR spectra of molecules and polymer, and stress-strain curves of polymer gels 28

5. References 42

1. Materials and methods

All reagents were purchased from commercial suppliers (J & K, Sigma-Aldrich, TCI, Energy, Macklin, Dieckmann, JenKem Technology, Acros Organics, and Cambridge Isotope Laboratories Inc.) and used without further purification unless otherwise noted. All solvents were of analytical grade (ACI Labscan and DUKSAN Pure Chemicals). Compounds 2-azidoethan-1-amine,[1] **Phen-CHO**,[2] **tetra PEG-DBCO**,[3] [Cu(**C8**)](PF_6),[4] **M8**[5] were prepared according to literature procedures.

Thin layer chromatography (TLC) was performed on silica gel 60 F254 (Merck, Germany, Aluminium sheet) and column chromatography was carried out using silica gel 60F (Silicycle, Canada). UPLC-ESI-MS were carried out using a Waters-Acquity UPLC H-Class system coupled with a QDa MS detector. HRMS spectra were obtained from a Bruker Impact II Ultra-High Resolution QTOF mass spectrometer. NMR spectra (including 2D NMR) were recorded on Bruker DPX spectrometers with working frequencies of 400 MHz, 500 MHz or 600 MHz for ^{1}H, and 101 MHz, 126 MHz or 151 MHz for ^{13}C, respectively. Chemical shifts are reported in ppm and referenced to solvent residues (for ^{1}H: $CDCl_3$: δ = 7.26 ppm, CD_3CN: δ = 1.94 ppm, CD_3OD: δ = 3.31 ppm; for ^{13}C: $CDCl_3$: δ = 77.16 ppm, CD_3CN: δ = 1.32 ppm, CD_3OD: δ = 49.00 ppm).

Rheological tests:

Methods regarding rheological tests are described in METHODS in the main text.

Mechanical tests:

Methods regarding mechanical tests are described in METHODS in the main text.

Gel preparation.

Typical gel preparation protocol is described in METHODS of the main text. We add a note here that gels for rheological testing were prepared with 20.0 mg of **tetra PEG-DBCO**, while those for tensile tests used a slightly higher mass of 21.6 mg, maintaining identical formulation and ensuring equivalent **tetra PEG-DBCO**/PC ratios across both samples.

DFT Simulation:

To study the force-induced behavior of supramolecular gels, DFT molecular simulations were performed on simplified constructs with varying topologies. A linear six-carbon small molecule served as the control. Initial molecular structures were constructed using Avogadro software and subsequently optimized without geometric constraints using the Gaussian 16 package. All computations employed the spin-unrestricted B3LYP hybrid functional with a mixed basis set: LANL2DZ for copper and 6-31G(d,p) for other atoms. During optimization, the convergence criterion for the density matrix was set to 10^8, and vibrational frequency analysis was conducted to verify the stability of the optimized structures. Following initial unconstrained optimization, molecular stretching was simulated by applying incremental displacements of 0.5 Å to 4.0 Å to the two terminal nitrogen atoms in opposite directions of different topologies, continuing until covalent bond rupture occurred. Throughout this process, these two nitrogen atoms remained fixed in position while the rest of the molecule was allowed to relax under constrained optimization at the same computational level. The energy difference between each stretched configuration and the initial optimized structure was calculated to determine the stretching relative energy

(E) change at each step. The relationship between E and displacement from equilibrium position (DEP)/terminal N-N distance (d_{NN}) were then plotted. We also monitored the evolution of the metal coordination geometry in the Cu-CAT and Cu-MAC complexes under deformation, specifically the changes in the N-Cu-N bond angle ($\alpha_{N\text{-}Cu\text{-}N}$) and Cu-N distances ($d_{Cu\text{-}N}$) with displacement from equilibrium.

Thermodynamic investigation:

The relative Gibbs free energy (ΔG_{ex}), enthalpy (ΔH_{ex}) and entropy changes (ΔS_{ex}) of the Cu(I) scrambling initiating from **[Cu-(MAC-Boc)]**$^+$ and **CAT-Boc** were studied by Variable-Temperature (VT) ^{1}H NMR employing the Van 't Hoff equation (Eq. S2). Stock solutions of **[Cu-(MAC-Boc)]**$^+$ and the metal-free [2] catenane ligands **CAT-Boc** at 1.6 mM and 4.0 mM, respectively, were prepared in DMSO-d_6. Sample of a 0.4:1 (mol/mol) mixture of **[Cu-(MAC-Boc)]**$^+$/**CAT-Boc** was prepared by mixing 125 μL each of the **[Cu-(MAC-Boc)]**$^+$ and **CAT-Boc** stock solutions, followed by addition of 250 μL of DMSO-d_6, then equilibrated at room temperature for 12 hours. The ^{1}H NMR spectra were collected at 318 K, 328 K 338 K, 348 K and 358 K, respectively. The spectra were collected after the reaction mixtures have been equilibrated at the designated temperature for 15 minutes within the NMR spectrometer. No significant difference was observed in the spectra collected after 15 and 20 minutes, suggesting that equilibrium has been reached. For each Cu^+ scrambling experiment, ln K_{ex} was plotted as a function of $1/T$. According to Eq. S2, $-\Delta H_{ex}/RT$ and $\Delta S_{ex}/R$ are respectively the slopes and intercepts of the linear plots.

$$[\mathbf{Cu\text{-}(MAC\text{-}Boc)}]^+ + \mathbf{CAT\text{-}Boc} \rightleftharpoons [\mathbf{Cu\text{-}(CAT\text{-}Boc)}]^+ + \mathbf{MAC\text{-}Boc} \qquad \triangle G_{ex}$$

$$\mathbf{CAT\text{-}Boc} + Cu^+ \rightleftharpoons [\mathbf{Cu\text{-}(CAT\text{-}Boc)}]^+ \qquad \triangle G_{\mathbf{CAT\text{-}Boc}}$$

$$\mathbf{MAC\text{-}Boc} + Cu^+ \rightleftharpoons [\mathbf{Cu\text{-}(MAC\text{-}Boc)}]^+ \qquad \triangle G_{\mathbf{MAC\text{-}Boc}}$$

$$\triangle G_{ex} = \triangle G_{\mathbf{CAT\text{-}Boc}} - \triangle G_{\mathbf{MAC\text{-}Boc}} = \triangle H_{ex} - T\triangle S_{ex} = -RT\ln K_{ex} \qquad \text{Eq. S1}$$

$$\ln K_{ex} = -\triangle H_{ex}/RT + \triangle S_{ex}/R \qquad \text{Eq. S2}$$

In addition, we have also independently measured the thermodynamic stability of the Cu(I) coordination to the model CAT-Boc and MAC-Boc using cyanide as a competitive ligand for UV-Vis measurement,[6] and a stability constant K_a of 9.52 (±0.45) × 10^{15} M^{-1} and 9.52 (±1.36) × 10^{14} M^{-1} was found respectively, corresponding to a relative stability of 10.0 and agrees well with the previously measured K_{ex} = 13.7.

2. Molecular synthesis and characterization

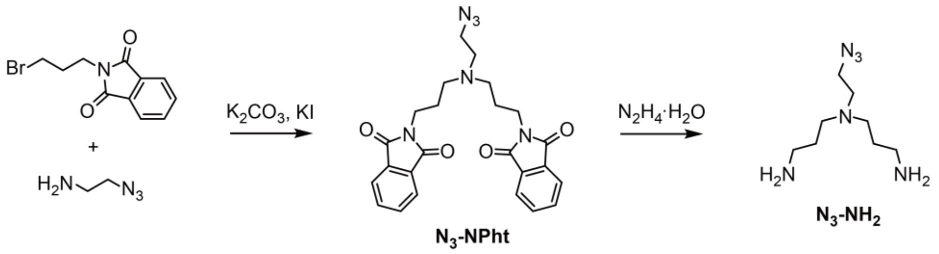

*Synthesis of **N_3-NPht***. A mixture of 2-azidoethan-1-amine (0.86 g, 10 mmol), *N*-(3-bromopropyl) phthalimide (6.70 g, 25 mmol), K_2CO_3 (4.14 g, 30 mmol) and KI (0.17 g, 1 mmol) in MeCN (100 mL) was heated at 80 °C for overnight. The reaction was cooled to room temperature and filtered, and the solvents were removed using a rotary evaporator. The residue was re-dissolved in 80 mL CH_2Cl_2, washed with water (2 × 100 mL) and brine (100 mL), dried over anhydrous Na_2SO_4 and filtered. Solvents were removed from the filtrate by a rotary evaporator and the residue was purified by column chromatography using hexane/ethyl acetate/CH_2Cl_2 (*v*/*v*/*v* = 12:8:1) as the eluent. The product was obtained as a white solid. Yield: 3.59 g, 78%. ^{1}H NMR (600 MHz, $CDCl_3$, 298 K) δ 7.84–7.79 (m, 4H), 7.72–7.68 (m, 4H), 3.75 (t, J = 7.0 Hz, 4H), 3.31 (t, J = 6.1 Hz, 2H), 2.65 (t, J = 6.2 Hz, 2H), 2.56 (t, J = 7.0 Hz, 4H), 1.83 (p, J = 7.1 Hz, 4H).^{13}C{^{1}H} NMR (151 MHz, $CDCl_3$, 298 K) δ 168.5, 134.0, 132.3, 123.3, 53.5, 52.0, 49.7, 36.3, 26.5. HRMS (ESI+): *m*/*z* $[M+H]^+$ calcd for $C_{24}H_{25}N_6O_4$: 461.1932; found: 461.1931.

*Synthesis of **N_3-NH_2***. Under an argon atmosphere, a mixture of **N_3-NPht** (1.0 g, 2.2

mmol) and hydrazine hydrate (0.7 mL, 14 mmol) in 50 mL MeOH was stirred for 24 hours at room temperature. The reaction mixture was filtered and solvents were removed from the filtrate using a rotary evaporator. The residue was re-dissolved in 30 mL CH_2Cl_2 and filtered, and the solvent was removed from the filtrate. If white solid was observed, the residue was re-dissolved in CH_2Cl_2 and filtered until a yellow oil was obtained upon solvent removal. Yield: 0.41 g, 93%. ^1H NMR (600 MHz, $CDCl_3$, 298 K) δ 3.28 (t, J = 6.1 Hz, 2H), 2.74 (t, J = 6.9 Hz, 4H), 2.64 (t, J = 6.1 Hz, 2H), 2.51 (t, J = 6.9 Hz, 4H), 1.61 (p, J = 6.9 Hz, 4H). $^{13}C\{^1H\}$ NMR (151 MHz, $CDCl_3$) δ 53.6, 52.2, 49.5, 40.5, 31.0. HRMS (ESI+): m/z $[M+H]^+$ calcd for $C_8H_{21}N_6$: 201.1822; found: 201.1820.

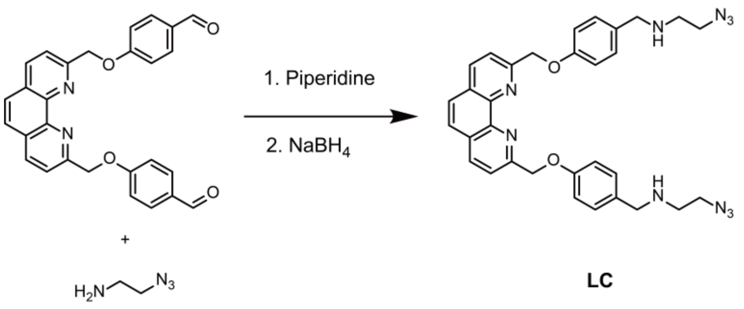

Synthesis of ***Linear control linker (LC)***. A mixture of **Phen-CHO** (0.90 g, 2.0 mmol) and 2-azidoethan-1-amine (0.87 g, 5.0 mmol) in 200 mL of 1:1 $CHCl_3/CH_3OH$ (100 mL/100 mL) was stirred for 30 minutes at room temperature, after which piperidine (0.5 mL) were added, the reaction was heated at 60 °C for 10 hours. The reaction mixture was cooled to 0 °C in an ice bath, $NaBH_4$ (0.38 g, 5.0 mmol) was added in portions and stirred in the ice bath for 1 hour. The mixture was warmed to room temperature and solvents were removed by a rotary evaporator. The yellow residue was re-dissolved in 100 mL CH_2Cl_2, washed with water (2 × 100 mL) and brine (100 mL), dried over anhydrous Na_2SO_4 and filtered. Solvents from the filtrate were removed by a rotary evaporator and the residue was purified by column chromatography using CH_2Cl_2/CH_3OH/triethylamine ($v/v/v$ = 100:1:1) as the eluent. The product was obtained

as a yellow solid. Yield: 0.96 g, 82%. ^{1}H NMR (500 MHz, $CDCl_3$, 298 K) δ 8.30 (d, *J* = 8.4 Hz, 2H), 7.94 (d, *J* = 8.2 Hz, 2H), 7.81 (s, 2H), 7.29 (d, *J* = 8.5 Hz, 4H), 7.04 (d, *J* = 8.4 Hz, 4H), 5.64 (s, 4H), 3.79 (s, 4H), 3.47 (t, *J* = 5.7 Hz, 4H), 2.83 (t, 4H). ^{13}C NMR (126 MHz, $CDCl_3$, 298 K) δ 158.5, 157.9, 145.4, 137.3, 129.8, 128.4, 126.5, 121.0, 115.0, 71.6, 52.9, 51.2, 47.7. HRMS (ESI+): *m/z* $[M+Na]^+$ calcd for $C_{32}H_{32}N_{10}O_2Na$: 611.2602; found: 611.2595.

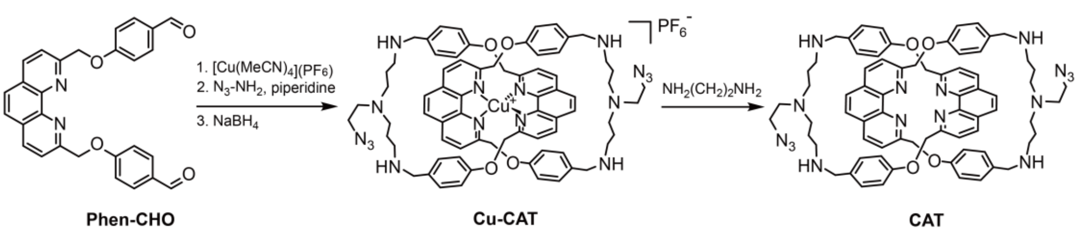

*Synthesis of **Cu-CAT***. Under an argon atmosphere, a mixture of **Phen-CHO** (0.54 g, 1.2 mmol) and $[Cu(CH_3CN)_4]PF_6$ (0.22 g, 0.6 mmol) in 300 mL of 5:3:2 $CHCl_3/CH_3CN/CH_3OH$ (150 mL/90 mL/60 mL) was stirred for 30 minutes at room temperature, after which **N_3-NH_2** (0.36 g, 1.8 mmol) and piperidine (0.5 mL) were added, the reaction was heated at 60 °C for 10 hours. The reaction mixture was cooled to 0 °C in an ice bath, $NaBH_4$ (0.14 g, 3.6 mmol) was added in portions and stirred in the ice bath for 1 hour. The mixture was warmed to room temperature and solvents were removed by a rotary evaporator. The dark red residue was re-dissolved in 50 mL CH_2Cl_2, washed with water (2 × 100 mL) and brine (100 mL), dried over anhydrous Na_2SO_4 and filtered. Solvents from the filtrate were removed by a rotary evaporator to afford an orange solid, which was washed by ethyl acetate (3 × 20 mL), re-dissolved in 30 mL CH_3CN, filtered and concentrated using a rotary evaporator to ~5 mL. Et_2O vapour was diffused to the concentrated solution in CH_3CN to give orange crystalline solids. The solvent was decanted and the product was rinsed by Et_2O (2 mL), followed by drying under vacuum to afford **Cu-CAT**. Yield: 0.53 g, 64%. ^{1}H NMR (500 MHz, DMSO-d_6, 298 K) δ 8.57 (d, *J* = 8.2 Hz, 4H), 8.04 (d, *J* = 8.2 Hz, 4H), 7.93 (s, 4H), 6.42 (d, *J* = 8.4 Hz, 8H), 5.79 (d, *J* = 8.5 Hz, 8H), 4.81 (s, 8H), 2.73–2.67 (m, 12H), 1.73 (t, *J* = 7.6

Hz, 8H). $^{13}C\{^1H\}$ NMR (126 MHz, DMSO-d_6, 298 K) δ 155.8, 153.7, 142.5, 137.6, 128.2, 127.8, 126.4, 125.5, 112.4, 70.7, 52.9, 52.3, 51.9, 48.4, 47.0, 27.7. HRMS (ESI+): *m*/*z* $[M-PF_6]^+$ calcd for $C_{72}H_{80}N_{16}O_4Cu$: 1295.5839; found: 1295.5846.

*Synthesis of **CAT***. To a solution of **Cu-CAT** (0.1 mmol, 144 mg) in CH_2Cl_2 (30 mL), ethylenediamine (2.0 mL) was added. The solution was stirred at room temperature for 15 minutes, and washed with H_2O (3 × 20 mL). Solvents were removed by a rotary evaporator, the afforded crude residue was purified by column chromatography using CH_2Cl_2/MeOH/Et_3N (*v*/*v*/*v* = 20:1:1) as the eluent. Product was obtained as a pale yellow solid. Yield: 96 mg, 78%. ^1H NMR (400 MHz, DMSO-d_6, 298 K) δ 8.56 (d, J = 8.2 Hz, 4H), 8.05 (s, 4H), 7.85 (d, J = 8.2 Hz, 4H), 6.80 (d, J = 8.3 Hz, 8H), 6.67 (d, J = 8.4 Hz, 8H), 5.28 (s, 8H), 3.07 (t, J = 5.8 Hz, 4H), 2.32 (t, J = 6.3 Hz, 4H), 2.21–2.11 (m, 8H), 2.07 (t, J = 6.6 Hz, 8H), 1.30–1.19 (m, 8H). $^{13}C\{^1H\}$ NMR (126 MHz, DMSO-d_6, 298 K) δ 157.1, 156.8, 144.8, 137.1, 133.2, 128.7, 128.1, 126.6, 122.2, 114.8, 72.6, 52.6, 52.4, 51.6, 48.4, 46.1, 27.1. HRMS (ESI+): *m*/*z* $[M+H]^+$ calcd for $C_{72}H_{81}N_{16}O_4$: 1233.6621; found: 1233.6625.

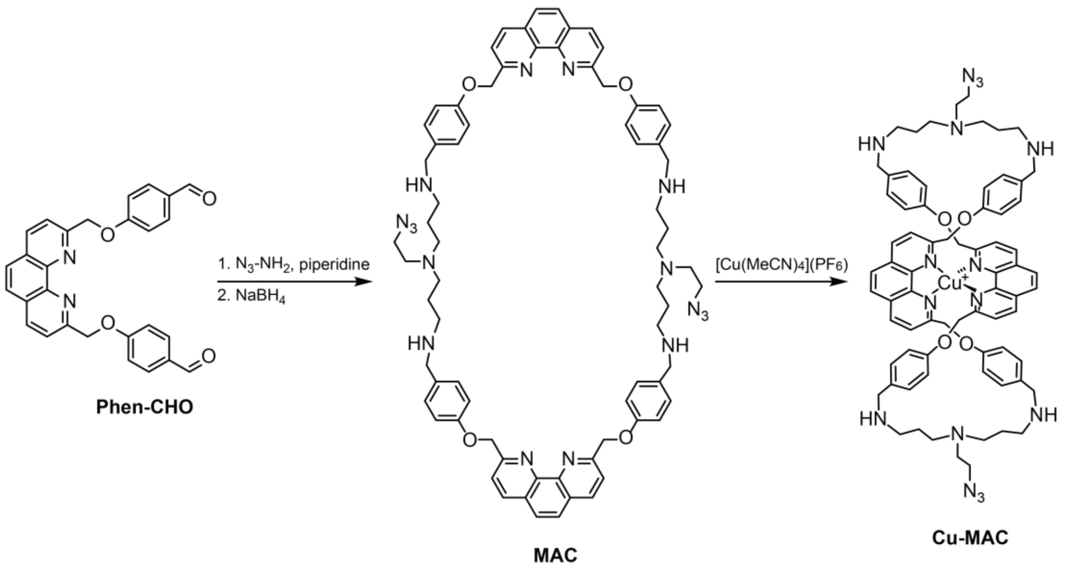

*Synthesis of **MAC***. A solution of **Phen-CHO** (0.45 g, 1.0 mmol), N_3-NH_2 (0.44 g, 2.2 mmol) and piperidine (0.5 mL) in a solvent mixture of $CHCl_3$/MeOH (450 mL/450 mL)

was heated at 60 °C for 12 hours. The reaction mixture was cooled to 0 °C in an ice bath, $NaBH_4$ (0.19 g, 5.0 mmol) was added in three portions, and the reaction mixture was recovered to room temperature and stirred for 1 hour. Solvents were removed by a rotary evaporator and the crude mixture was purified by column chromatography using CH_2Cl_2/Et_3N/MeOH (*v*/*v*/*v* = 25:1:1) as the eluent. The product was obtained as a pale yellow solid. Yield: 0.16 g, 13%. ^{1}H NMR (600 MHz, DMSO-d_6, 298 K) δ 8.50 (d, *J* = 8.3 Hz, 4H), 7.97 (s, 4H), 7.83 (d, *J* = 8.3 Hz, 4H), 7.50 (d, *J* = 8.4 Hz, 8H), 7.12 (d, *J* = 8.4 Hz, 8H), 5.45 (s, 8H), 4.01 (s, 8H), 2.84 (t, *J* = 7.7 Hz, 8H), 2.53 (t, *J* = 6.0 Hz, 4H), 2.46 (t, *J* = 6.7 Hz, 8H), 1.77 (p, *J* = 6.9 Hz, 8H). $^{13}C\{^{1}H\}$ NMR (151 MHz, DMSO-d_6, 298 K) δ 157.3, 157.1, 144.5, 137.1, 132.4, 129.5, 127.9, 126.4, 120.9, 114.5, 70.9, 53.1, 52.1, 51.4, 48.4, 46.4, 26.4. HRMS (ESI+): *m*/*z* calcd. for $C_{72}H_{80}N_{16}O_4Na$ $[M+Na]^+$: 1255.6440, found: 1255.6407.

.

Synthesis of ***Cu-MAC***. Under an argon atmosphere, a mixture of **MAC** (25 mg, 20 μmol) and $[Cu(MeCN)_4](PF_6)$ (8.3 mg, 22 μmol) in $CHCl_3$/CH_3CN/MeOH (5 mL, *v*/*v*/*v* = 5:3:2) was stirred at room temperature for 2 hours. The reaction mixture was filtered through Celite pad, and the solvents were removed using a rotary evaporator to afford the product as a dark red solid. Yield: 31 mg, 93%. ^{1}H NMR (500 MHz, DMSO-d_6, 298 K) δ 8.82 (d, *J* = 8.4 Hz, 4H), 8.17 (s, 4H), 8.13 (d, *J* = 8.5 Hz, 4H), 6.81 (d, *J* = 8.1 Hz, 8H), 6.20 (d, *J* = 7.9 Hz, 8H), 5.25 (d, *J* = 13.6 Hz, 4H), 4.90 (d, *J* = 13.7 Hz, 4H), 3.48 (s, 8H), 3.16 (t, *J* = 6.8 Hz, 8H), 2.40 (d, *J* = 6.4 Hz, 4H), 2.24 (d, *J* = 7.8 Hz, 8H), 1.34 (s, 8H), 1.24 (s, 4H). $^{13}C\{^{1}H\}$ NMR (151 MHz, DMSO-d_6, 298 K) δ 156.1, 155.8, 142.5, 138.4, 132.2, 129.1, 128.7, 126.8, 124.1, 113.7, 70.0, 52.9, 52.0, 51.3, 48.3, 45.4, 26.3. HRMS (ESI+): *m*/*z* calcd. for $C_{72}H_{80}CuN_{16}O_4$ $[M-PF_6]^+$: 1295.5830, found: 1295.5839.

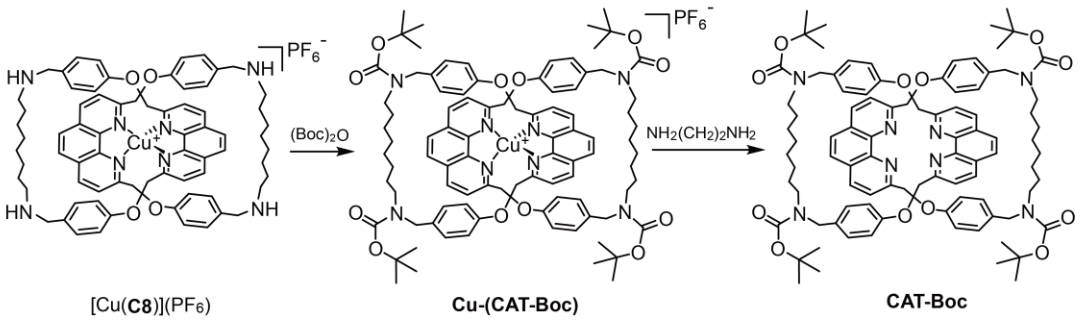

*Synthesis of **Cu-(CAT-Boc)***. To a solution of [Cu(**C8**)](PF_6) (53 mg, 40 μmol) in CH_2Cl_2 (10 mL) at 0 °C, di-*t*-butyl dicarbonate (53 mg, 0.24 mmol) was added dropwise. The reaction solution was recovered to room temperature and stirred for 2 hours. Solvents were removed using a rotary evaporator, the red crude mixture was washed with hexane (3 × 5 mL) and dried under vacuum to afford the product as a red solid. Yield: 56 mg, 81%. ^{1}H NMR (400 MHz, DMSO-d_6, 298 K) δ 8.64 (br, 4H), 8.09 (br, 4H), 7.96 (br, 4H), 6.38 (br, 8H), 5.86 (br, 8H), 4.85 (br, 8H), 4.06 (br, 8H), 3.31 (br, 8H), 3.01 (br, 8H), 1.53 (br, 8H), 1.39 (br, 36H), 1.20 (br, 8H). ^{13}C{^{1}H} NMR (151 MHz, DMSO-d_6, 298 K) δ 155.9, 154.9, 154.3, 142.5, 137.6, 130.6, 129.7, 128.3, 126.9, 126.3, 125.3, 113.0, 78.6, 70.5, 40.0, 39.8, 39.7, 39.5, 39.4, 39.2, 39.1, 30.7, 28.3, 28.1, 27.9, 26.4, 25.1. HRMS (ESI+): *m/z* $[M\text{-}PF_6]^+$ calcd for $C_{92}H_{112}CuN_8O_{12}$: 1584.7722; found: 1584.7702.

*Synthesis of **CAT-Boc***. To a solution of **Cu-(CAT-Boc)** (52 mg, 30 μmol) in CH_2Cl_2 (5 mL) was added ethylenediamine (0.6 mL), and the mixture was stirred at room temperature for 1 hour. The mixture was washed by deionized water (3 × 10 mL) and dried under vacuum to afford **CAT-Boc** as a white powder. Yield: 36 mg, 80%. ^{1}H NMR (500 MHz, DMSO-d_6, 298 K) δ 8.34 (br, 4H), 7.82 (br, 4H), 7.60 (br, 4H), 6.89 (br, 17H), 5.34 (br, 8H), 4.15 (br, 8H), 3.30 (br, 8H), 2.72 (br, 8H), 1.34 (br, 36H), 0.90 (br, 8H), 0.81 (br, 8H). ^{13}C{^{1}H} NMR (151 MHz, DMSO-d_6, 298 K) δ 157.6, 156.7, 155.0, 144.8, 136.8, 130.4, 128.5, 128.3, 126.3, 115.2, 78.3, 72.0, 28.8, 28.7, 28.3, 28.0, 26.4. HRMS (ESI+): *m/z* $[M+2H]^{2+}$ calcd for $C_{92}H_{114}N_8O_{12}$: 761.4273; found: 761.4265.

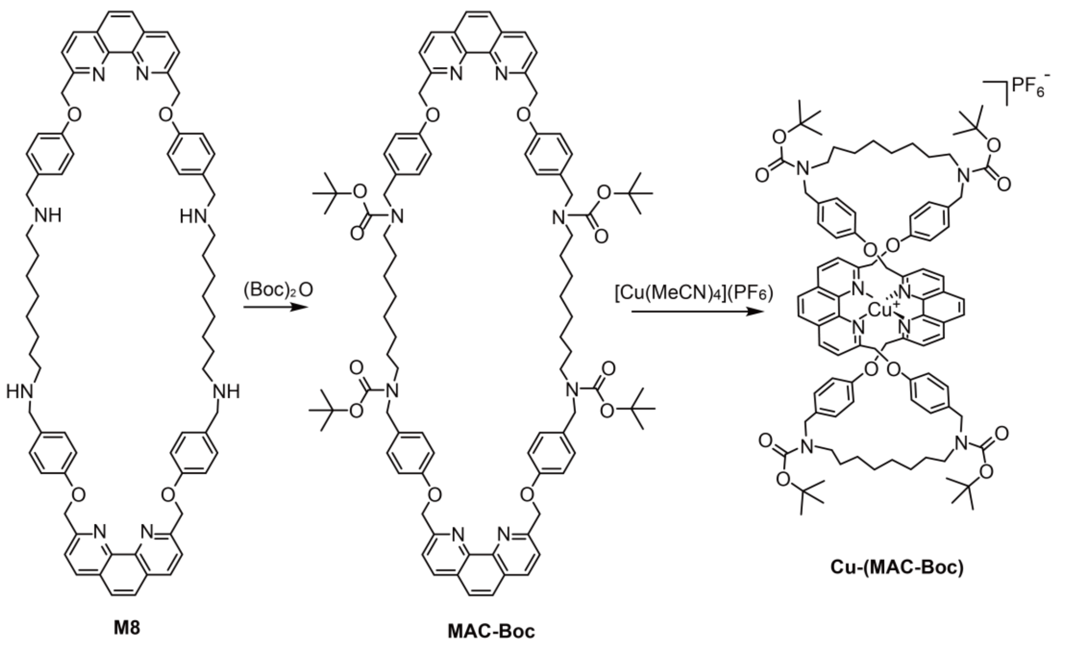

*Synthesis of **MAC-Boc***. To a solution of **M8** (34 mg, 30 μmol) in CH_2Cl_2/MeOH (5 mL, *v*/*v* = 4:1) at 0 ºC, di-*t*-butyl dicarbonate (53 mg, 0.18 mmol) was added dropwise. The reaction was recovered to room temperature and stirred at room temperature for 2 hours. Solvents were removed using a rotary evaporator and the light-yellow crude was washed with hexane (3 × 5 mL) to afford the product as a white solid. Yield: 40 mg, 89%. ^{1}H NMR (400 MHz, DMSO-d_6, 298 K) δ 8.48 (d, *J* = 8.4 Hz, 4H), 7.95 (s, 4H), 7.85 (d, *J* = 8.4 Hz, 4H), 7.14 (d, *J* = 8.6 Hz, 8H), 7.04 (d, *J* = 8.5 Hz, 8H), 5.45 (s, 8H), 4.25 (s, 8H), 3.01 (br, 8H), 1.37 (br, 44H), 1.07 (br, 8H). ^{13}C{^{1}H} NMR (151 MHz, DMSO-d_6, 298 K) δ 157.3, 155.1, 144.6, 137.2, 131.2, 128.9, 128.6, 128.0, 126.4, 121.1, 114.8, 78.5, 71.0, 31.3, 28.3, 28.0, 27.6, 25.9. HRMS (ESI+): *m*/*z* $[M+2H]^{2+}$ calcd for $C_{92}H_{114}N_8O_{12}$: 761.4273; found: 761.4269.

*Synthesis of **Cu-(MAC-Boc)***. Under an argon atmosphere, a mixture of **MAC-Boc** (30.4 mg, 0.02 mmol) and $[Cu(MeCN)_4](PF_6)$ (8.3 mg, 0.022 mmol) in $CHCl_3/CH_3CN$/MeOH (5 mL, *v*/*v*/*v* = 5:3:2) was stirred at room temperature for 2 hours. The reaction mixture was filtered through a Celite pad. Solvents were removed using a rotary evaporator to afford the product as a dark red solid. Yield: 33 mg, 95%. ^{1}H NMR

(600 MHz, DMSO-d_6, 298 K) δ 8.76 (br, 4H), 8.13 (s, 4H), 8.08 (d, J = 8.3 Hz, 4H), 6.69 (br, 8H), 6.11 (br, 8H), 5.12 (br, 4H), 4.84 (br, 4H), 4.20–4.02 (m, 8H), 3.31 (br, 8H), 2.92 (br, 8H), 1.38 (s, 36H), 0.88–0.80 (m, 16H). $^{13}C\{^1H\}$ NMR (151 MHz, DMSO-d_6, 298 K) δ 156.0, 155.5, 155.3, 142.4, 138.3, 131.6, 128.7, 128.5, 126.8, 124.3, 113.4, 78.6, 70.1, 31.3, 30.7, 28.1, 27.4, 25.6. HRMS (ESI+): m/z $[M-PF_6]^+$ calcd for $C_{92}H_{112}CuN_8O_{12}$: 1584.7722; found: 1584.7723.

Synthesis of ***tetra PEG-DBCO****.*

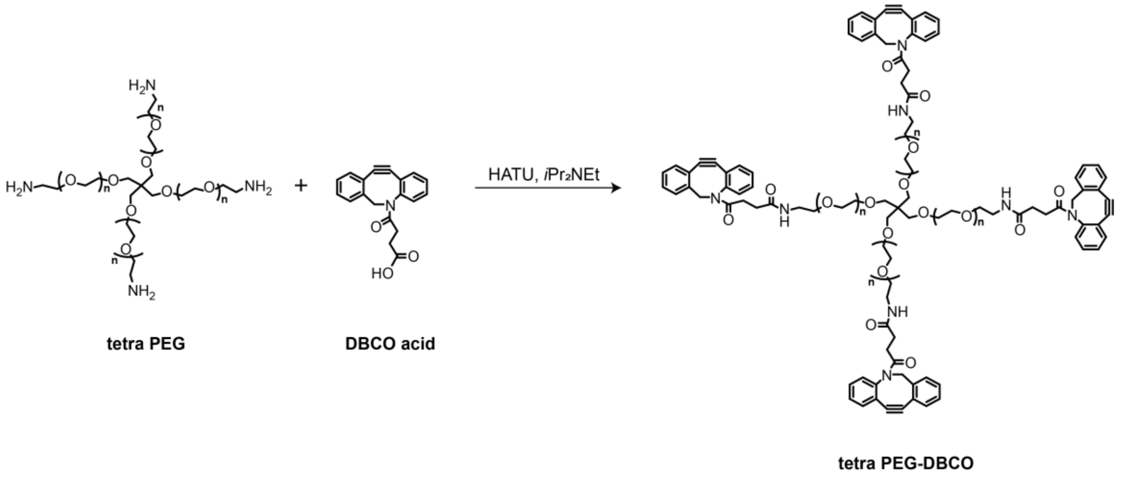

Tetra amino-DBCO-PEG (Mn = 10 kDa, 1000 mg, 0.1 mmol) was dissolved in dry DMF (2 mL). Separately, **DBCO-acid** (250 mg, 0.9 mmol) and HATU (312 mg, 0.82 mmol) were dissolved in dry DMF (2 mL) with stirring at room temperature for 5 min, yielding a pale-yellow solution. This solution was transferred to the **tetra amino-PEG** mixture at 0 °C, with the reaction vessel rinsed using dry DMF (0.5 mL) to ensure complete transfer. After adding anhydrous iPr$_2$NEt (286 μL), the reaction mixture was stirred at room temperature for 36 h. The crude product was precipitated by dropwise addition into cold diethyl ether (repeated 3×), affording a pale-yellow solid. This material was dissolved in methanol and purified by extensive dialysis against methanol to remove small-molecule impurities. Dialysis was performed using a dialysis membrane with a molecular weight cut-off (MWCO) of 2 kDa. Concentration of the dialyzed solution yielded **tetra PEG-DBCO** as a pale-yellow solid. According to ^{1}H

NMR, the end-functionalization was above 90%. ^{1}H NMR (400 MHz, $CDCl_3$, 298 K) δ 7.68 (d, J = 9.0 Hz, 4H), 7.53-7.51 (m, 4H), 7.41-7.24 (m, 24H), 6.23 (br, 4H), 5.16 (d, J = 13.9 Hz, 4H), 3.83-3.32 (m, 912H), 2.86-2.78 (m, 4H), 2.51-2.43 (m, 4H), 2.21-2.15 (m, 4H), 1.97-1.91 (m, 4H).

3. Supplementary figures

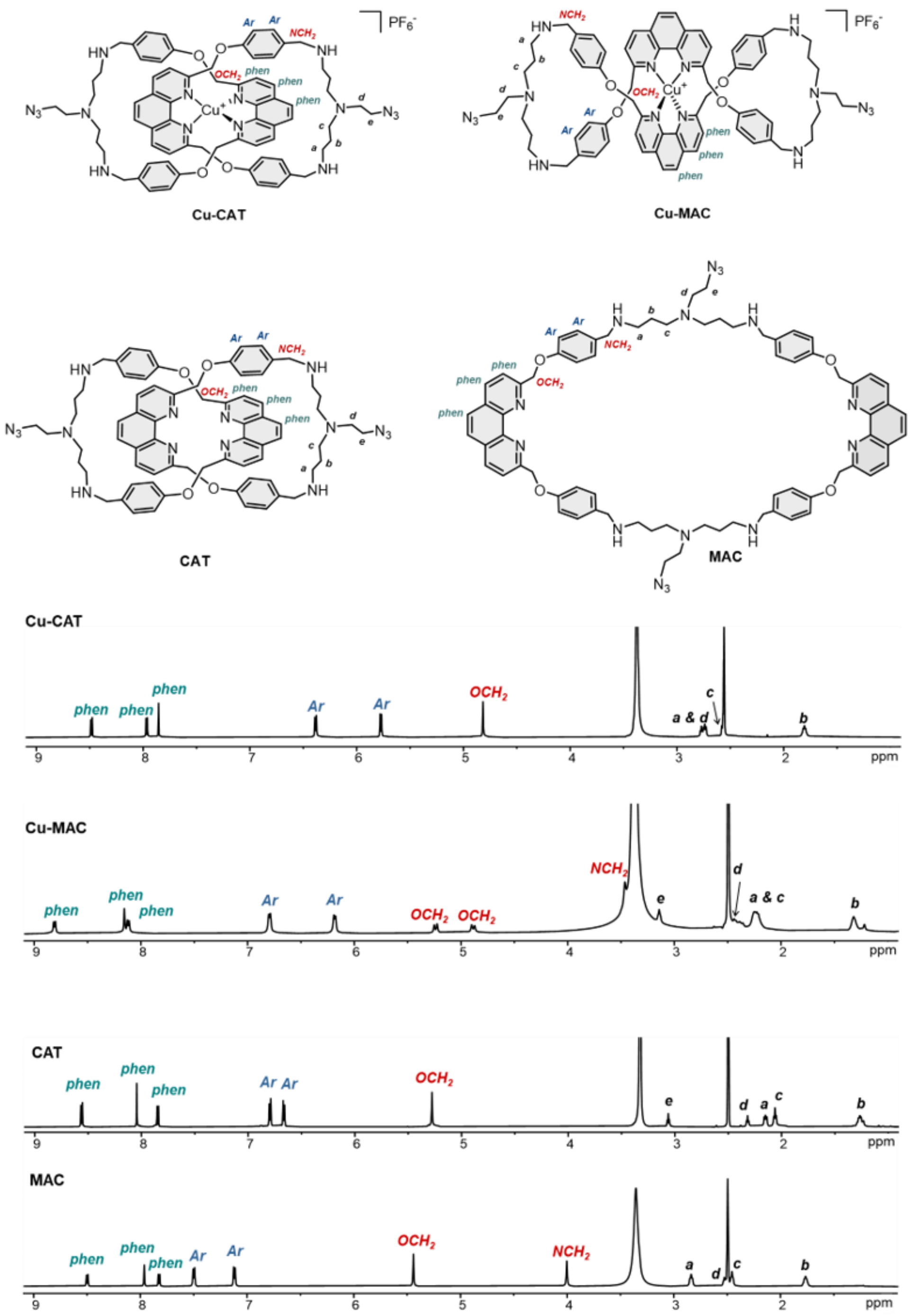

Figure S1. ^{1}H NMR (500 MHz, DMSO-d_6, 298 K) spectra of Cu-CAT, Cu-MAC, CAT and MAC, respectively, showing full peak assignment.

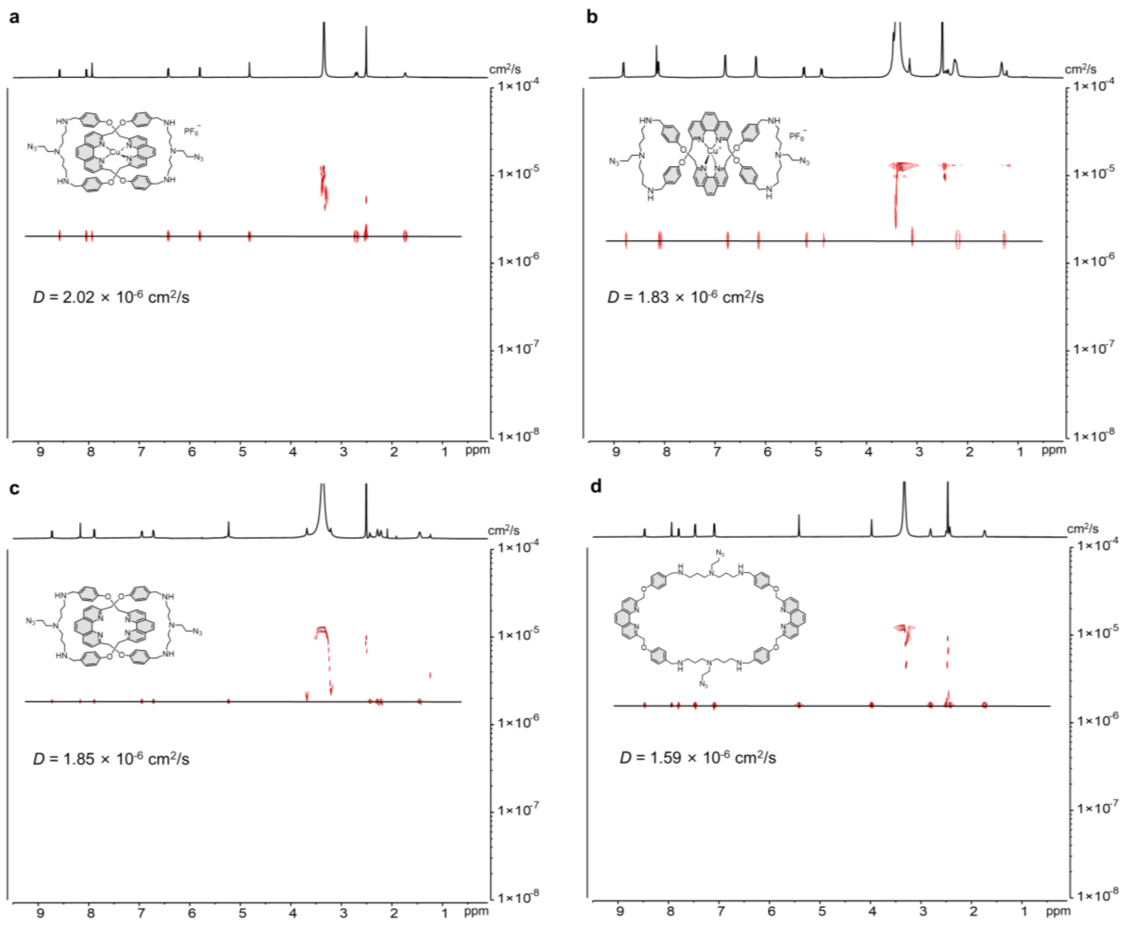

Figure S2. 2D DOSY NMR spectra (600 MHz, DMSO-d_6, 298 K) of (**a**) Cu-CAT, (**b**) Cu-MAC, (**c**) CAT and (**d**) MAC.

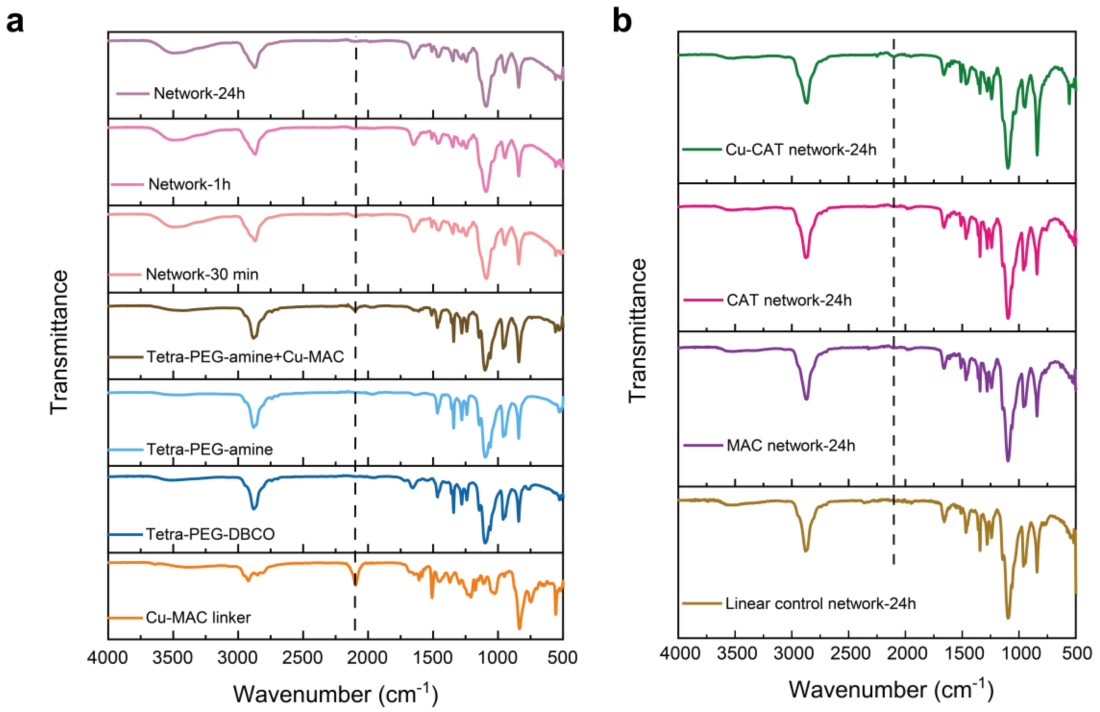

Figure S3. FT-IR spectra of (**a**) Cu-MAC linker, tetra-PEG-DBCO, tetra-PEG-amine, tetra-PEG-amine mixed with Cu-MAC linker, and Cu-MAC with tetra-PEG-DBCO at 30 min, 1h and 24h. (**b**) FT-IR spectra of Cu-CAT, CAT, MAC and linear control networks at 24h. The spectra indicate a near complete conversion of azide peak at ~2100 cm^{-1}, suggesting high conversion and effectiveness of SPAAC in forming gels.

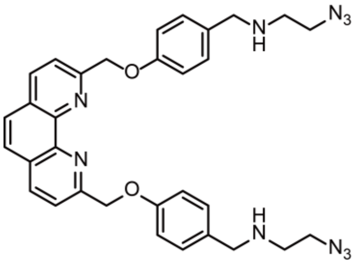

Figure S4. Chemical structure of linear control linker containing a phenanthroline group.

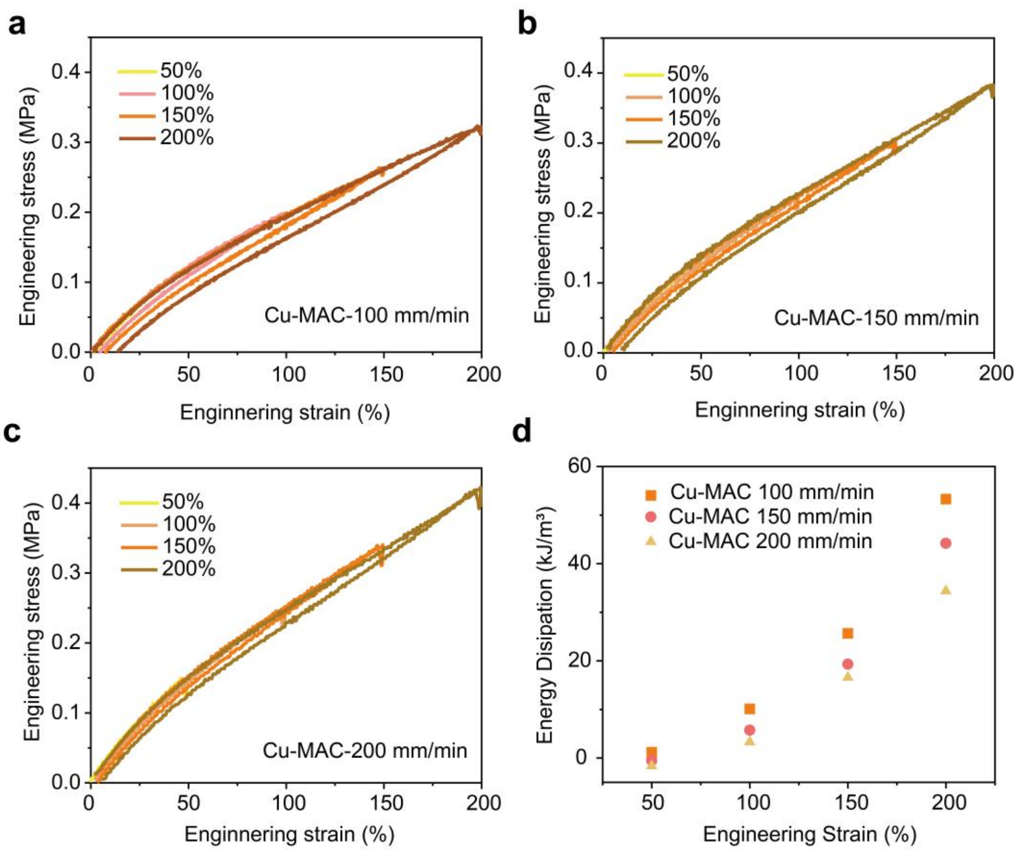

Figure S5. Cyclic tensile curves of Cu-MAC gel at 50%-200% strains under (**a**) 100, (**b**) 150 and (**c**) 200 mm/min and (**d**) corresponding energy dissipation.

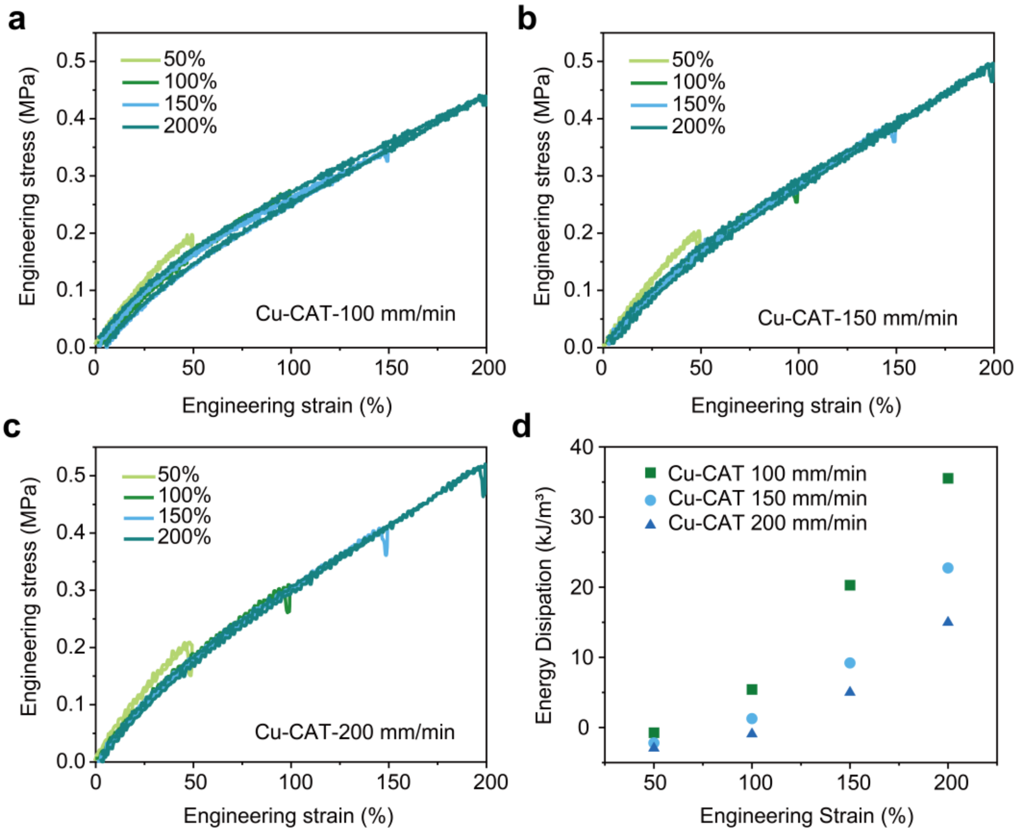

Figure S6. Cyclic tensile curves of Cu-CAT gel at 50%-200% strains under (**a**) 100, (**b**) 150 and (**c**) 200 mm/min and (**d**) corresponding energy dissipation.

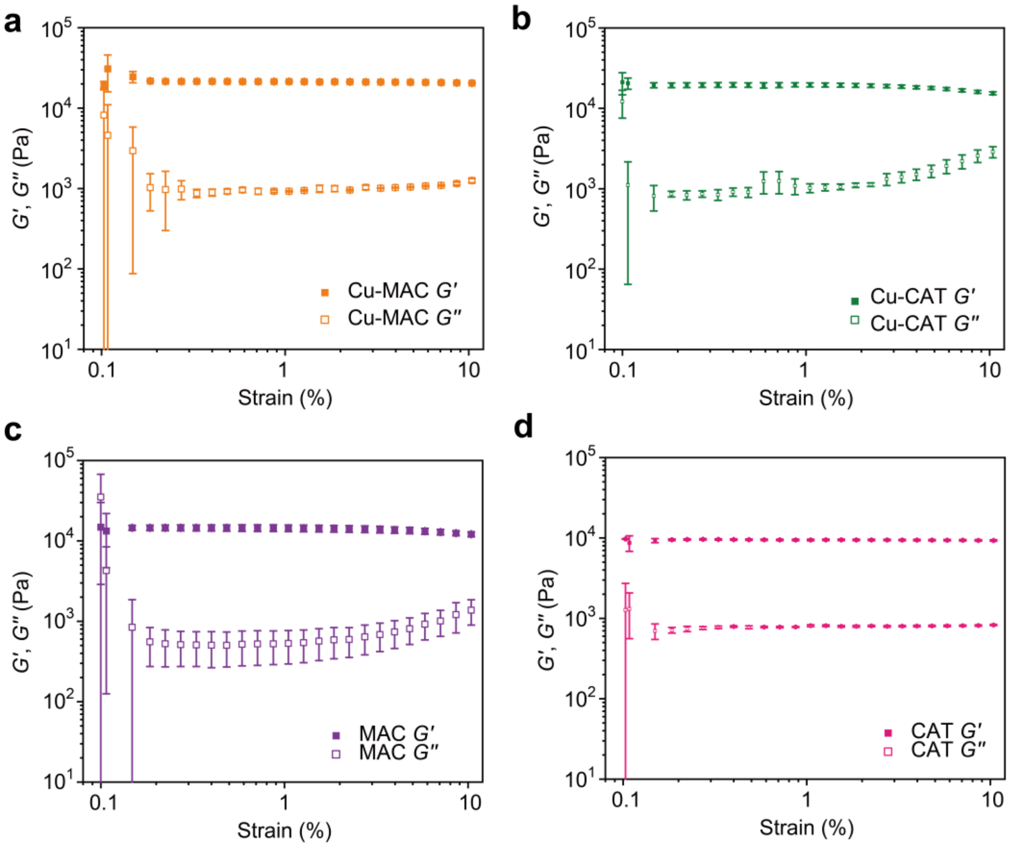

Figure S7. Amplitude sweeps of different gels showing the linear regime under 0.1%-10% strain at 1 rad/s.

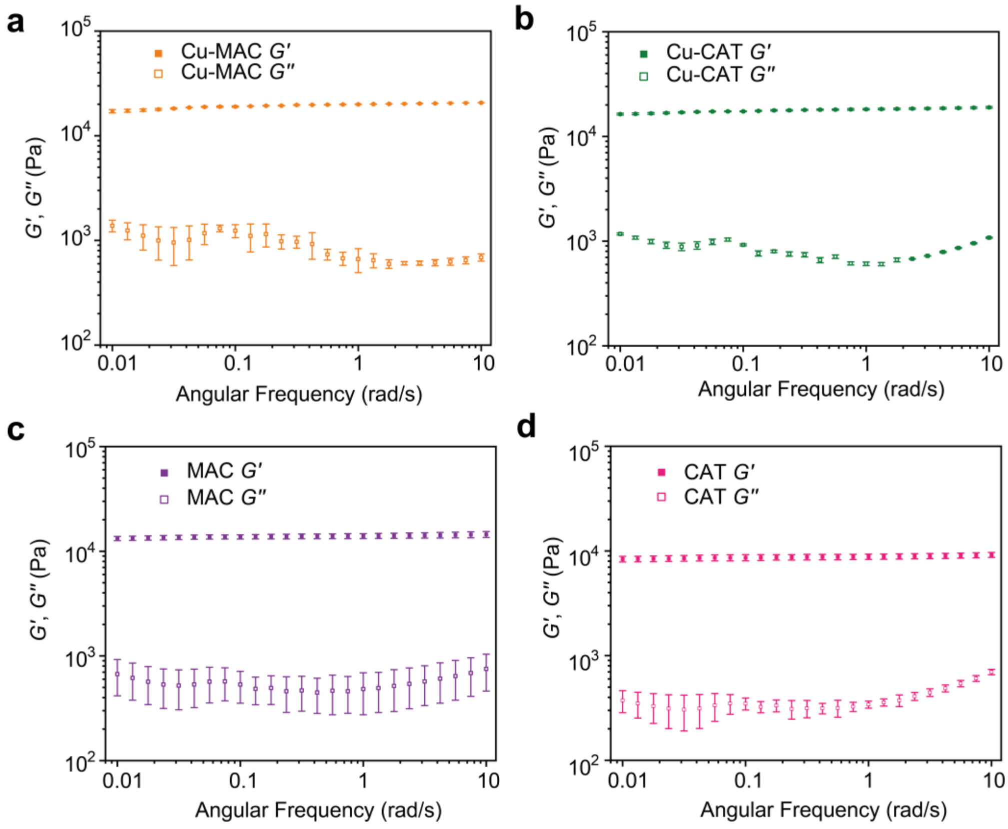

Figure S8. Frequency sweeps of different gels showing the linear regime at 1% strain from 0.01-10 rad/s.

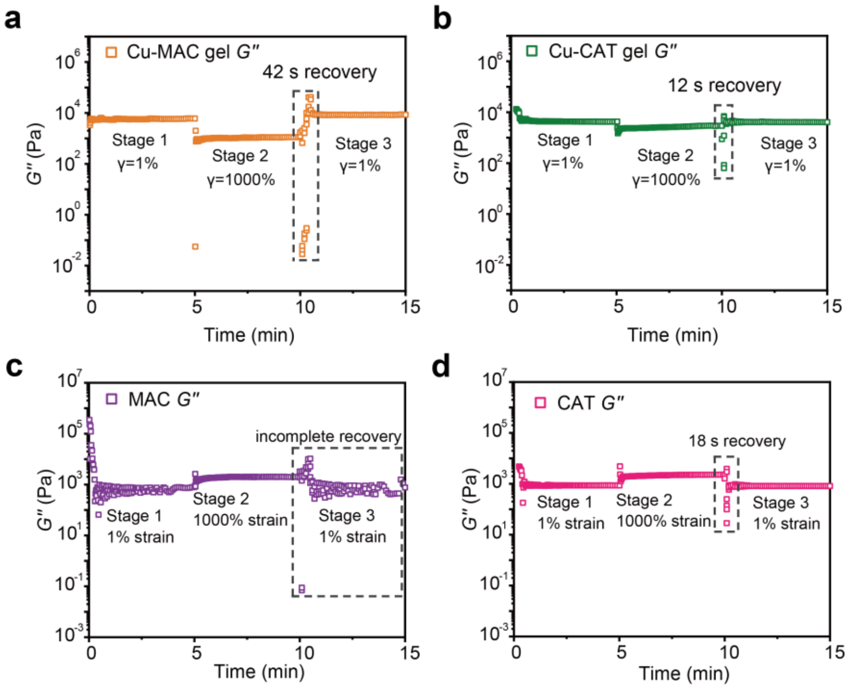

Figure S9. Three-stage rheological time sweeps. (**a**) Cu-MAC gel, (**b**) Cu-CAT gel, (**c**) MAC gel, and (**d**) CAT gel.

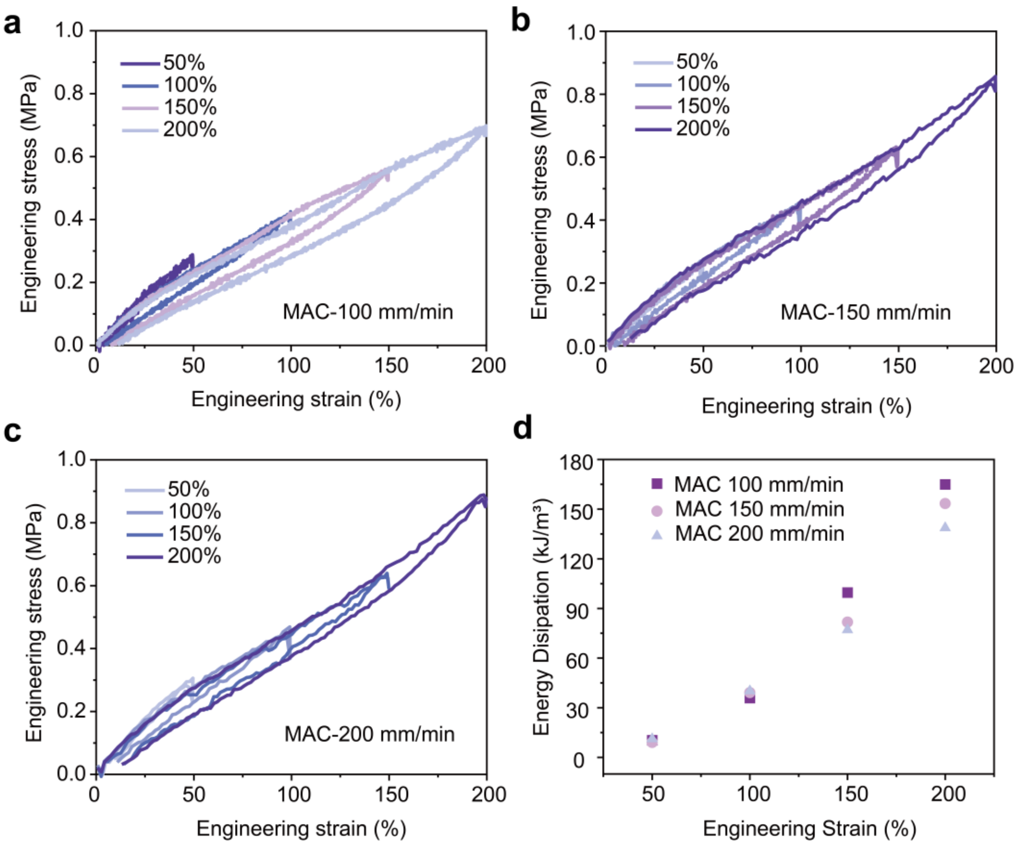

Figure S10. Cyclic tensile curves of MAC gel at 50%-200% strains under (**a**) 100, (**b**) 150 and (**c**) 200 mm/min and (**d**) corresponding energy dissipation.

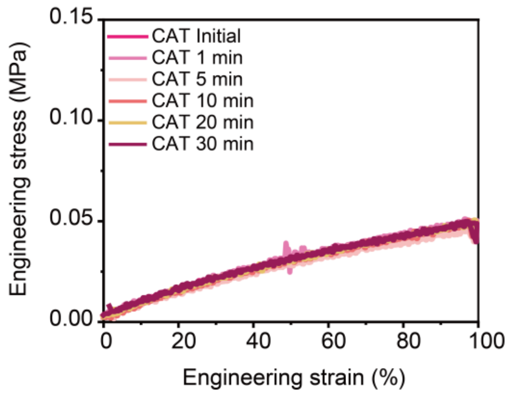

Figure S11. Cyclic tensile test of CAT gels with recovery intervals ranging from 1 to 30 min at 100% strain.

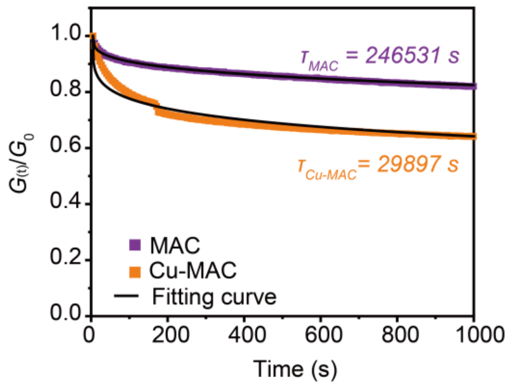

Figure S12. Stress relaxation profiles of MAC and Cu-MAC gels comparing their characteristic relaxation time.

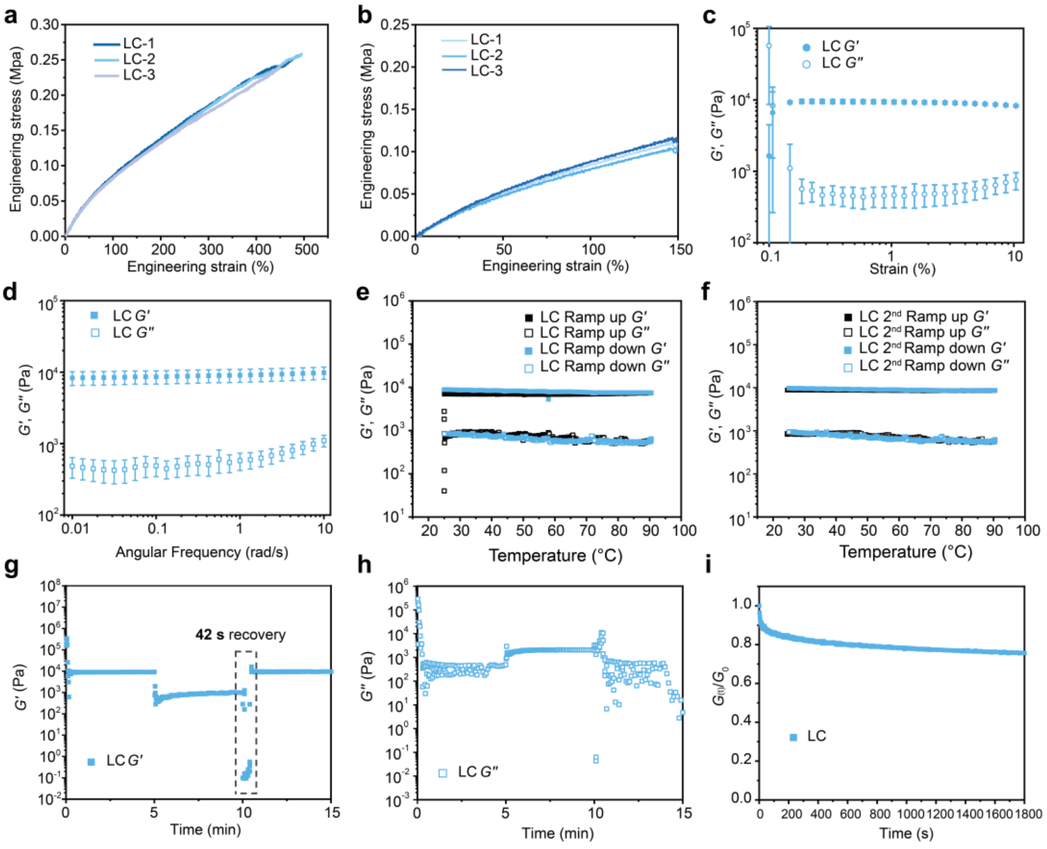

Figure S13. Mechanical and rheological properties of LC gel. (**a**) Stress-strain curves of LC gel (strain rate, 100 mm/min). (**b**) Cyclic tensile curves of LC gel at 150% strain. Each curve represents a different individual sample taken from the same gel. (**c**) Amplitude sweeps of LC gel showing the linear regime at 1 rad/s at 25°C. (**d**) Frequency sweeps of LC gel showing the linear regime at 1% strain at 25°C. (**e**) Temperature ramp

curve of LC gel. (**f**) The second temperature ramp curve of LC gel. (**g**, **h**) Three-stage rheological time sweeps for LC gel. (**i**) Normalized stress relaxation of LC gel under a shear strain of 10% at 25 ℃.

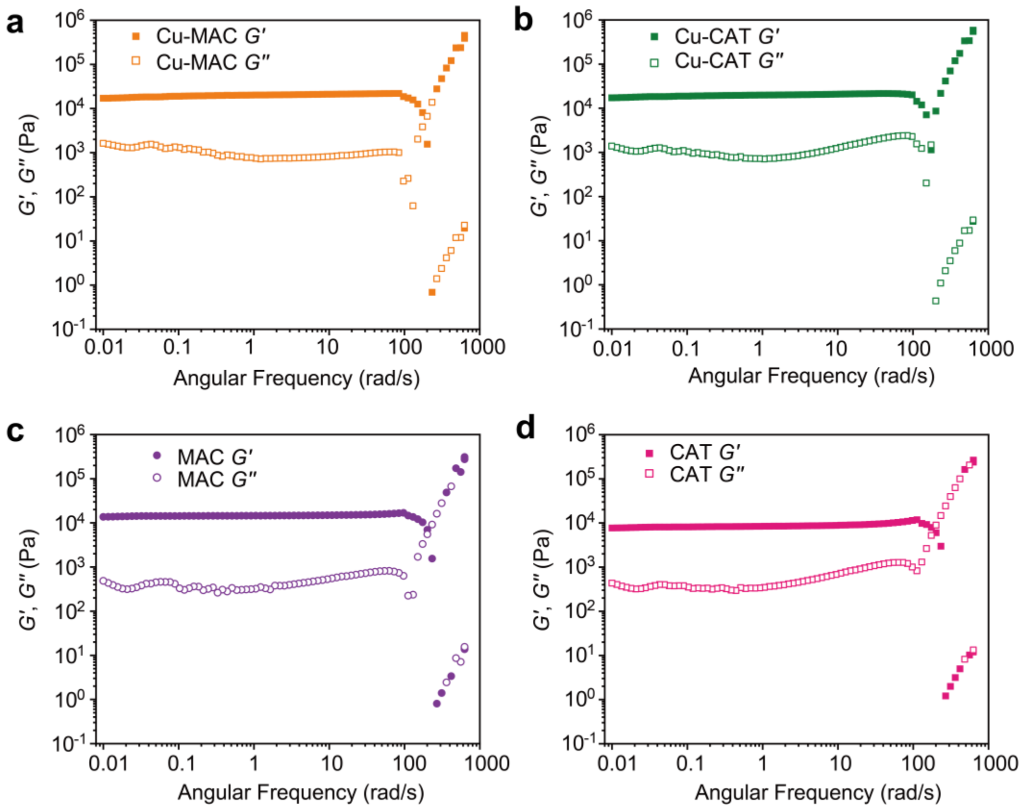

Figure S14. Frequency sweep of different gels at 1% strain from 0.01-1000 rad/s.

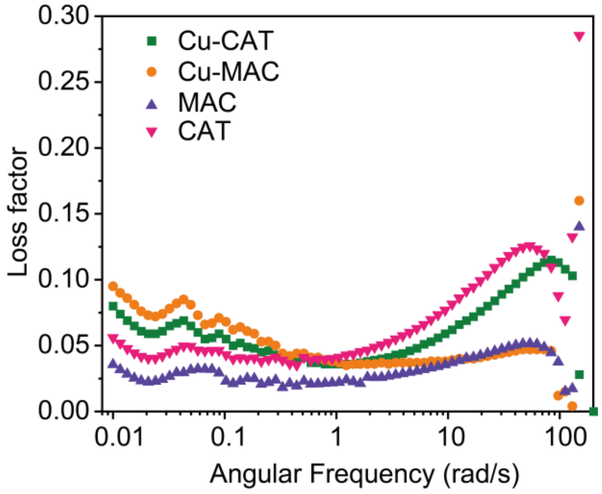

Figure S15. Loss factor of different gels at 1% strain from 0.01-1000 rad/s.

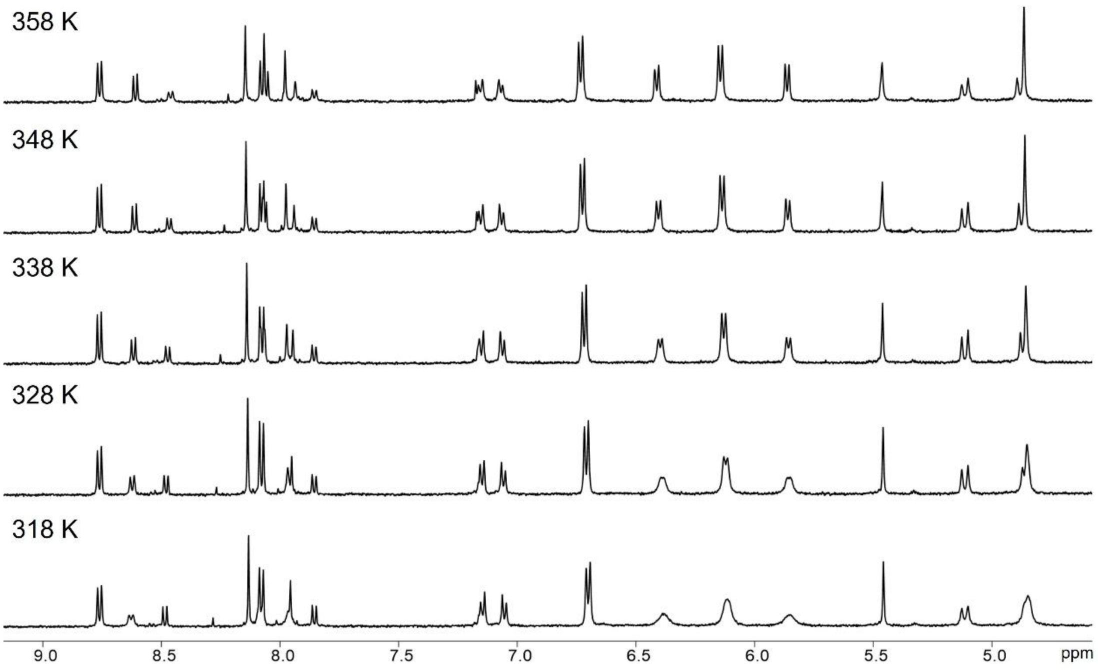

Figure S16. Partial ^{1}H NMR spectra of the equilibrated 0.4:1 (mol/mol) [**Cu-(MAC-Boc)**]$^{+}$/**CAT-Boc** at 318 K, 328 K, 338 K, 348 K and 358 K (500 MHz, DMSO-d_6).

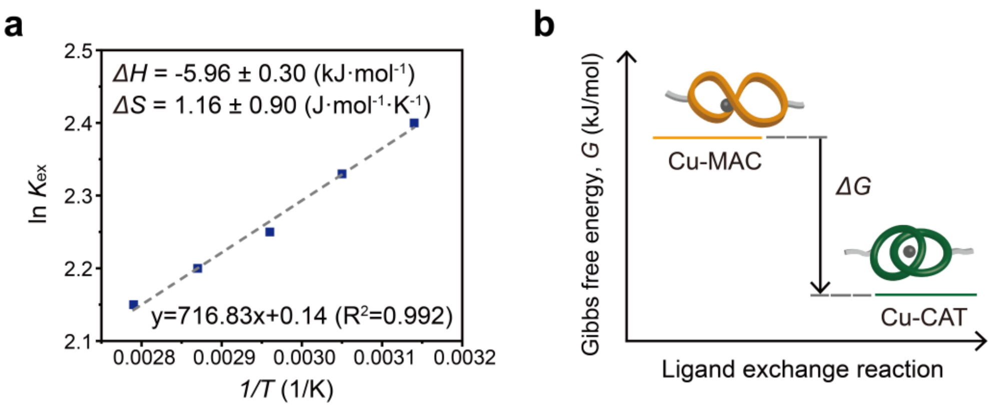

Figure S17. (**a**) Van 't Hoff fitting plot. (**b**) Gibbs energy profile showing relative energy of Cu-MAC and Cu-CAT.

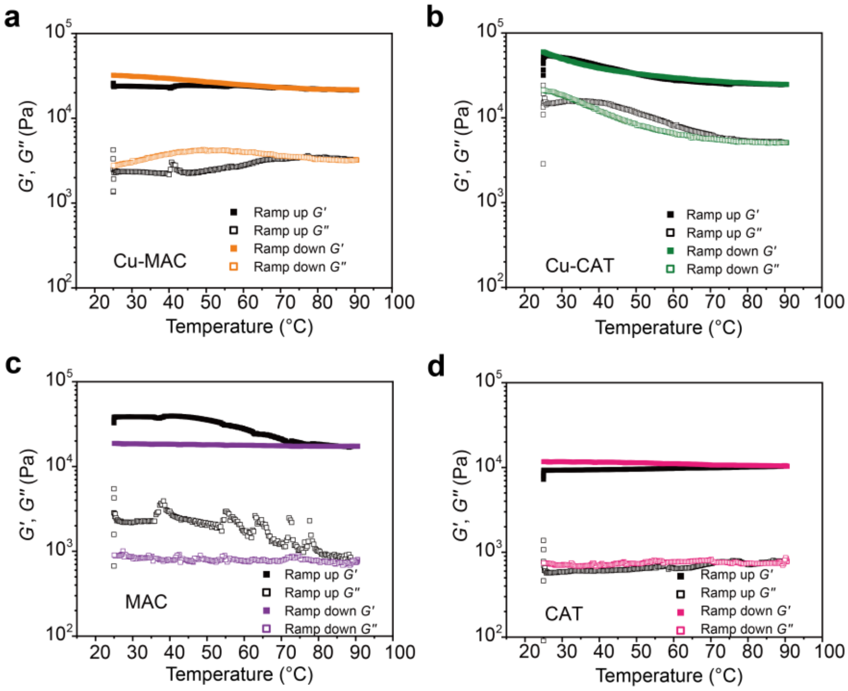

Figure S18. Data showing G' and G'' for the first cycle of heating-cooling temperature ramp from 25 to 90 °C. (**a**) Cu-MAC gel, (**b**) Cu-CAT gel, (**c**) MAC gel and (**d**) CAT gel.

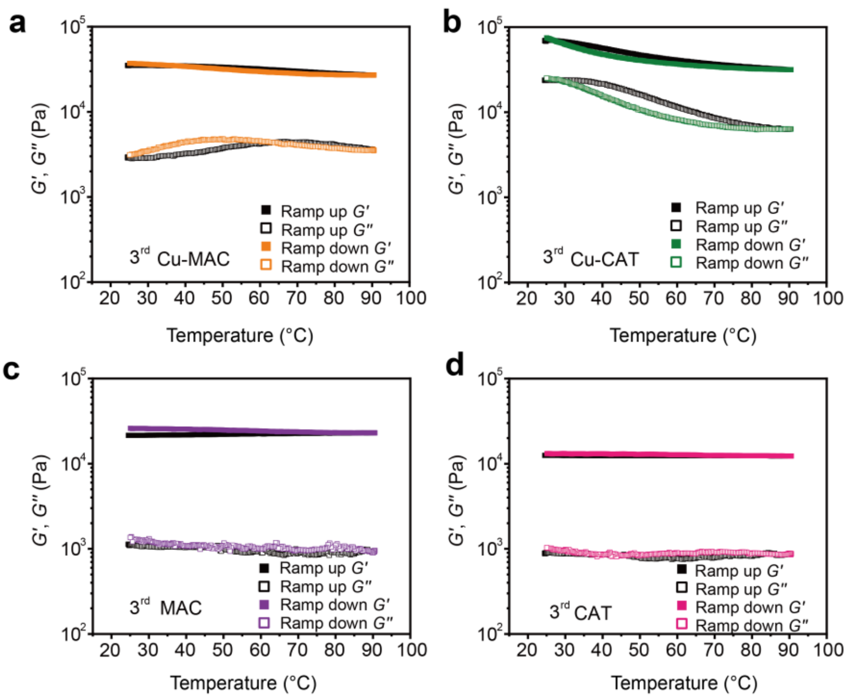

Figure S19. Data showing G' and G'' for the third cycle of heating-cooling temperature ramp from 25 to 90 °C. (**a**) Cu-MAC gel, (**b**) Cu-CAT gel, (**c**) MAC gel and (**d**) CAT gel. The second cycle is shown in main text Figure. The successive cycles do not exhibit further changes.

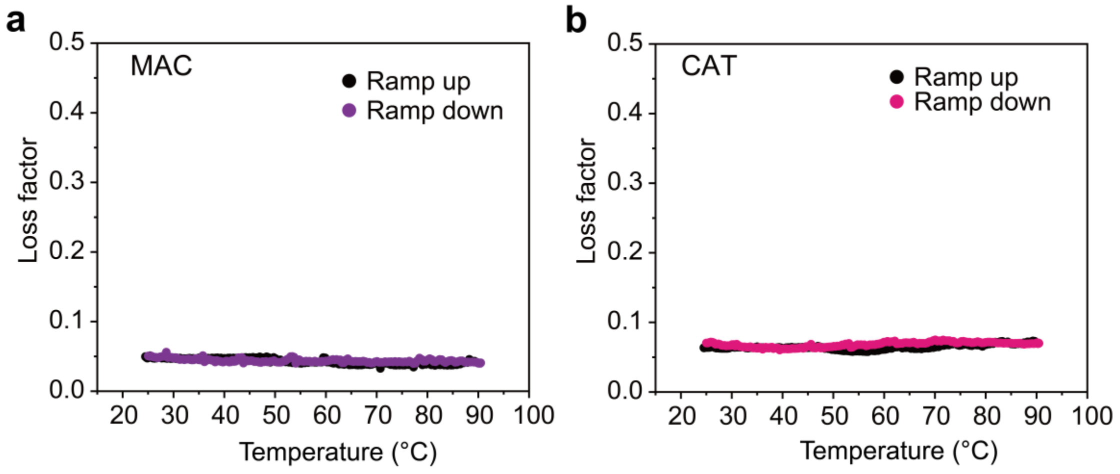

Figure S20. The loss factors of (**a**) MAC and (**b**) CAT gels in a representative temperature cycle.

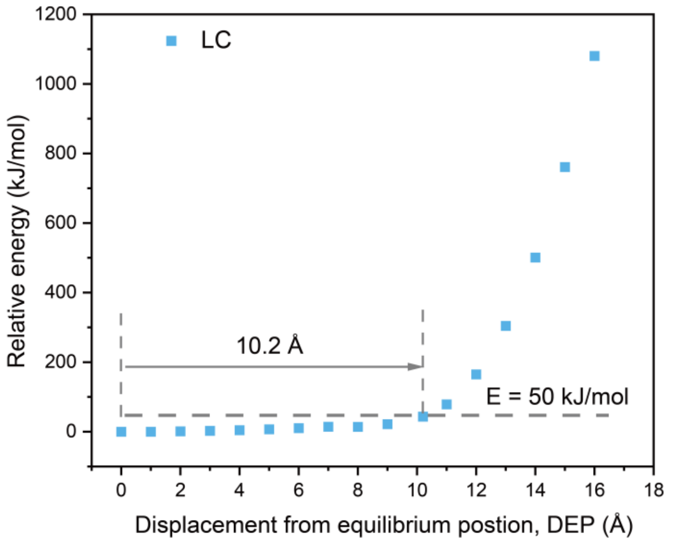

Figure S21. Relative energy by DFT simulation of linear control linker as a function of displacement from equilibrium position.

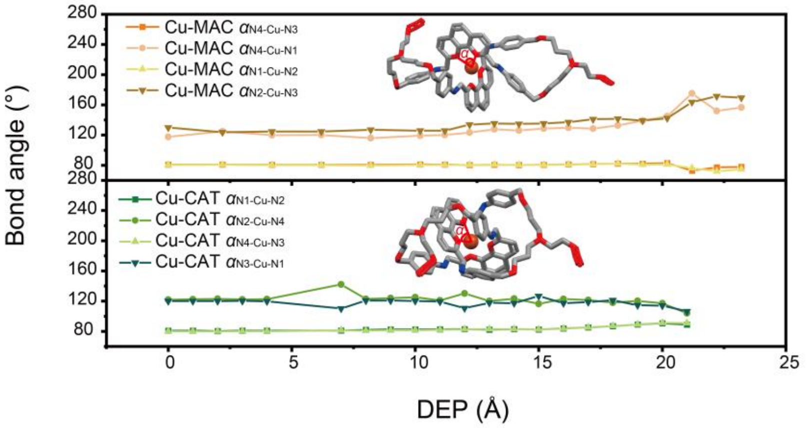

Figure S22. Metal-coordination bond angle vs. displacement from equilibrium position in Cu-MAC and Cu-CAT linkers. The bond angles remain relatively unchanged before DEP = 20Å, indicative of little bond dissociation during gel stretching.

4. Appendix: ^{1}H and ^{13}C NMR spectra of molecules and polymer, and stress-strain curves of polymer gels

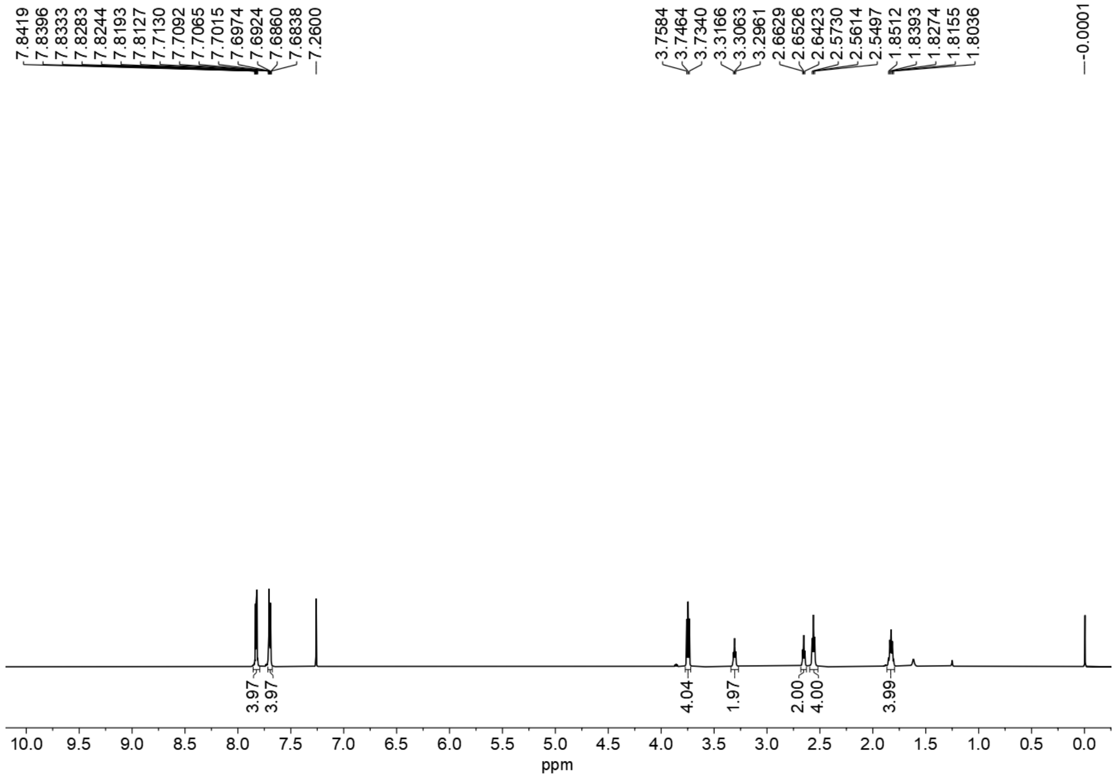

Figure S23. ^{1}H NMR (600 MHz, $CDCl_3$, 298 K) of **N_3-NPht**.

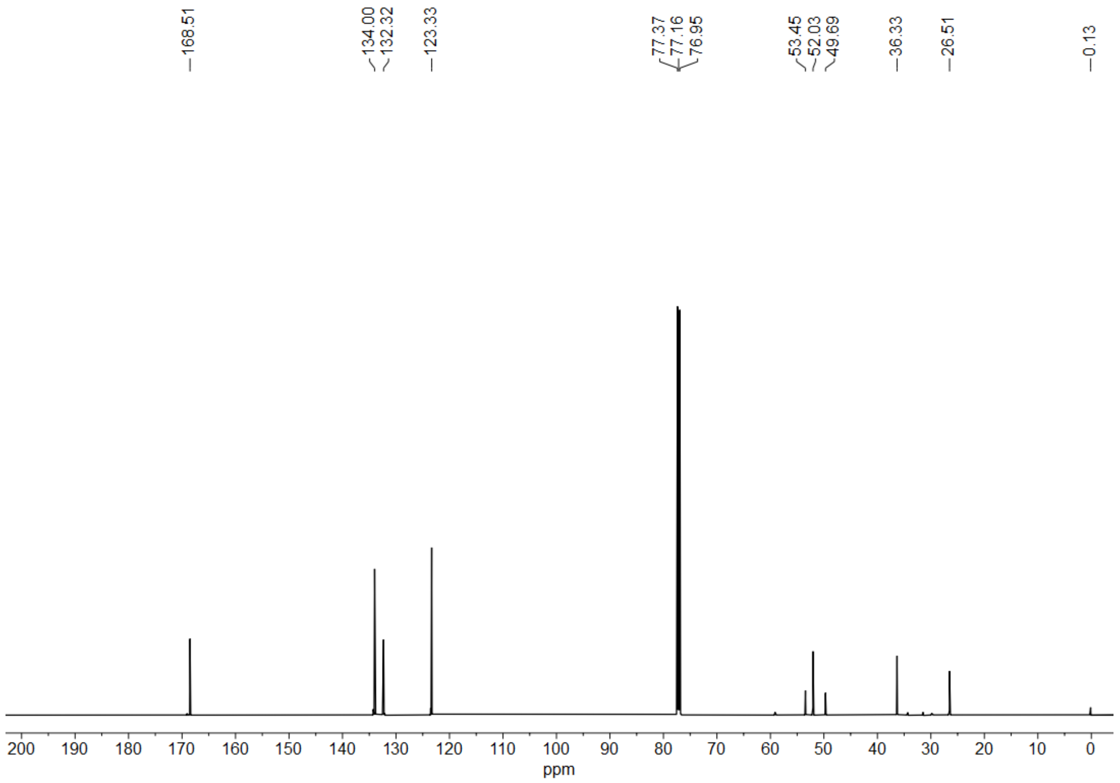

Figure S24. $^{13}C\{^{1}H\}$ NMR (151 MHz, $CDCl_3$, 298 K) of **N_3-NPht**.

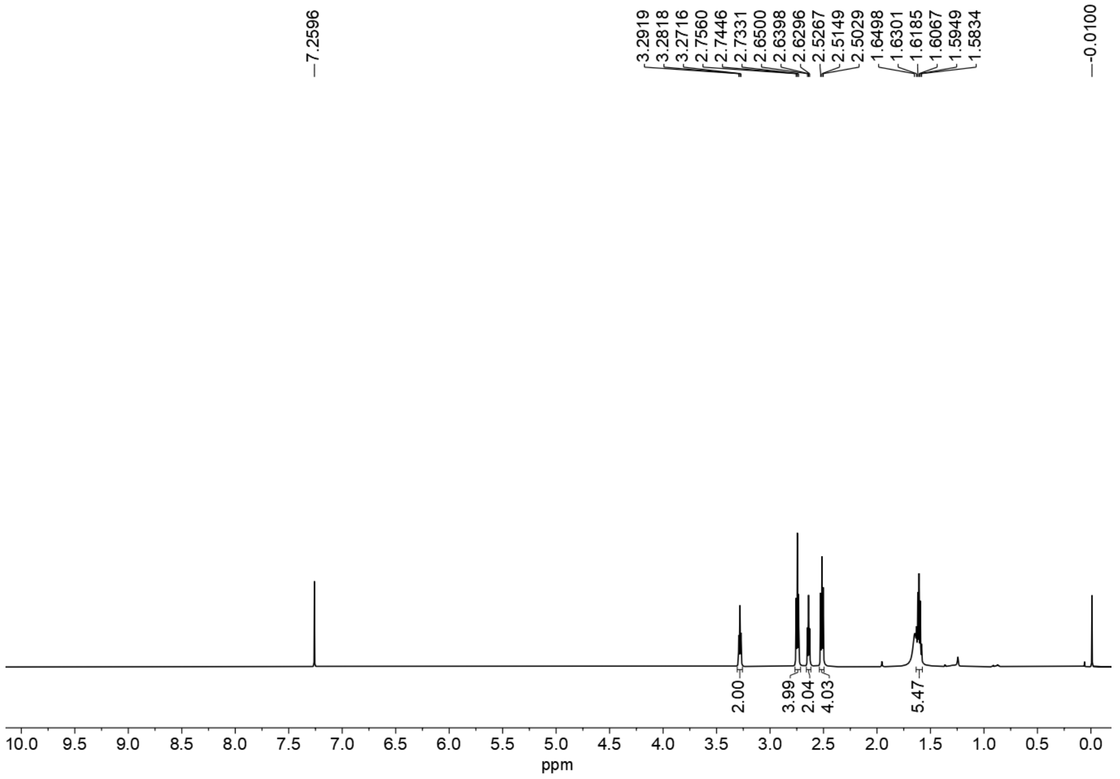

Figure S25. ^{1}H NMR (600 MHz, $CDCl_3$, 298 K) of **N_3-NH_2**.

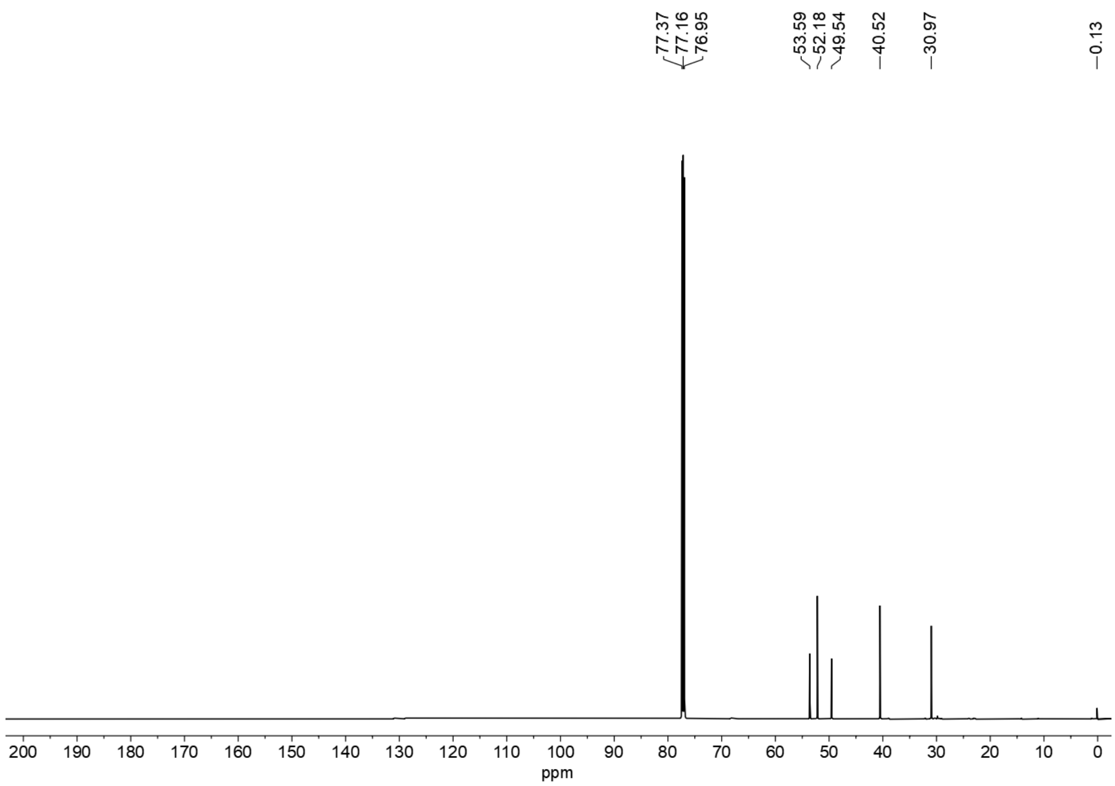

Figure S26. $^{13}C\{^1H\}$ NMR (151 MHz, $CDCl_3$, 298 K) of **N_3-NH_2**.

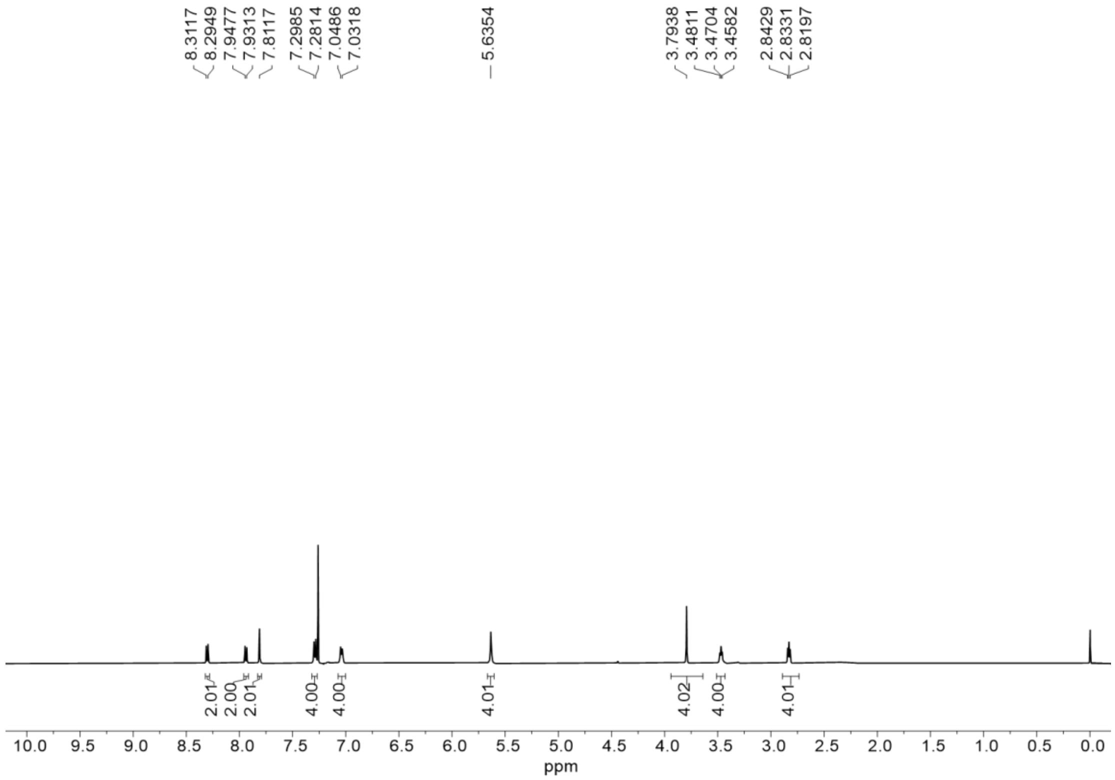

Figure S27. ^1H NMR (500 MHz, $CDCl_3$, 298 K) of **LC**.

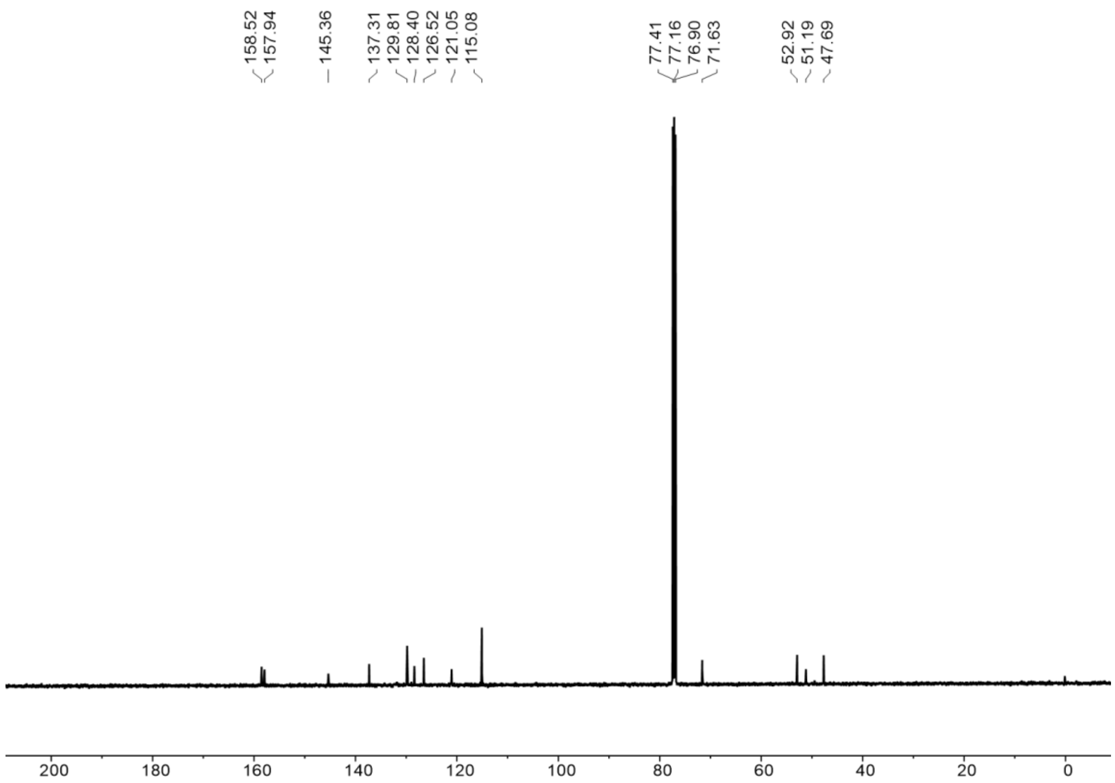

Figure S28. $^{13}C\{^{1}H\}$ NMR (126 MHz, DMSO-d_6, 298 K) of **LC**.

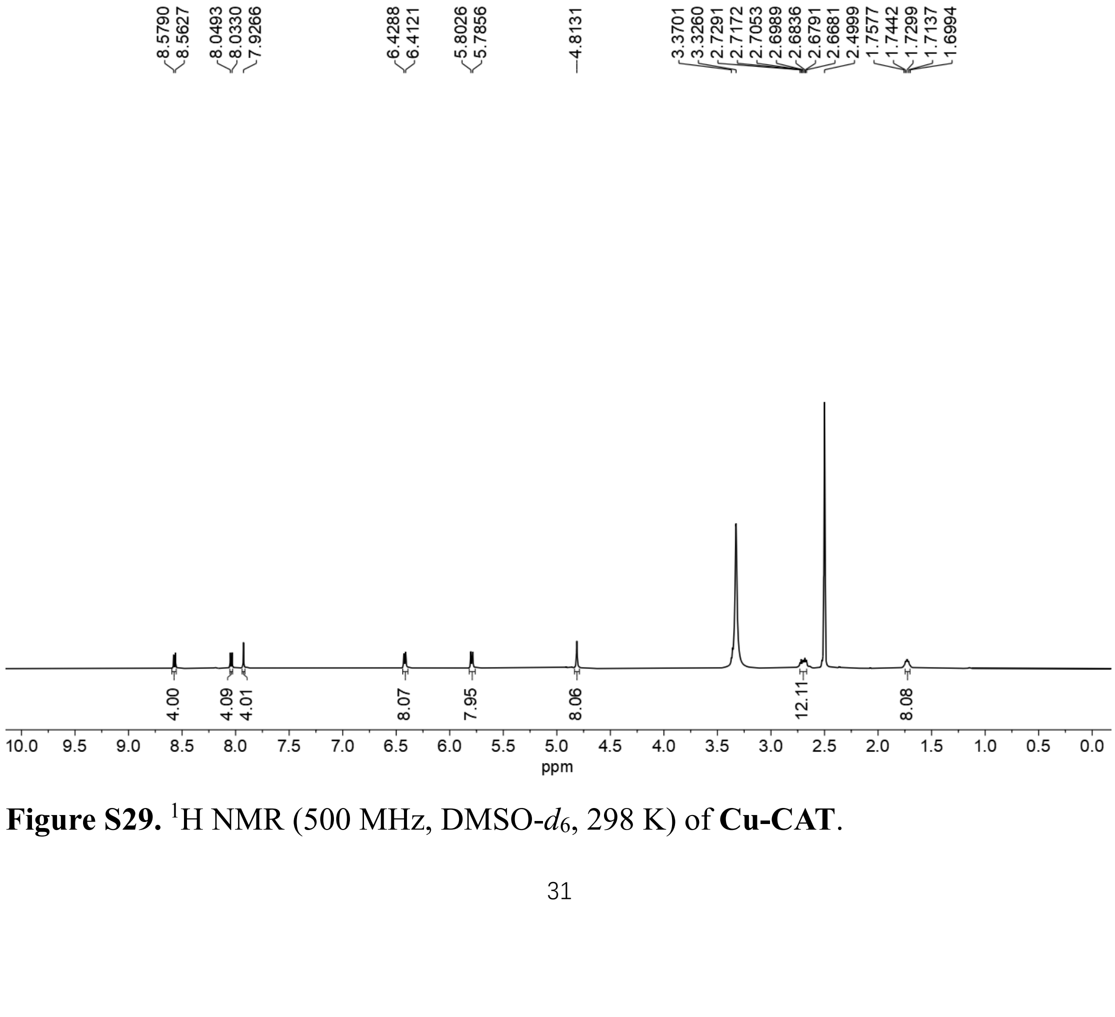

Figure S29. ^{1}H NMR (500 MHz, DMSO-d_6, 298 K) of **Cu-CAT**.

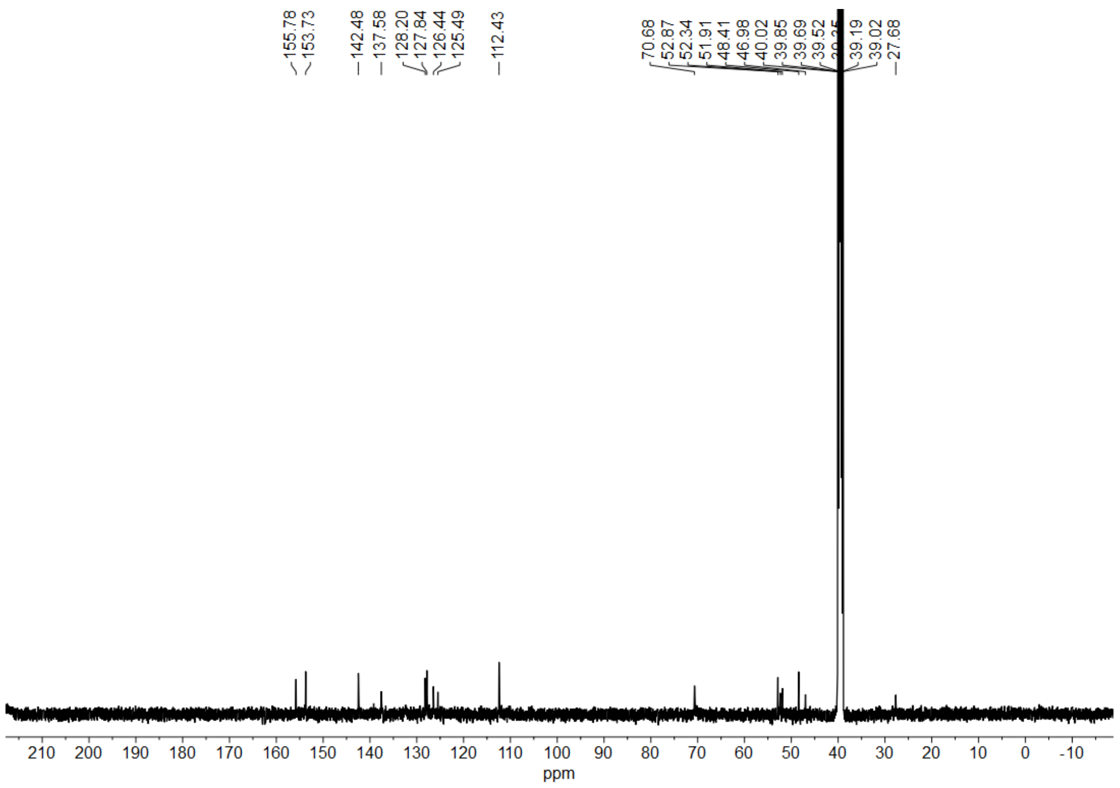

Figure S30. $^{13}C\{^{1}H\}$ NMR (126 MHz, DMSO-d_6, 298 K) of **Cu-CAT**.

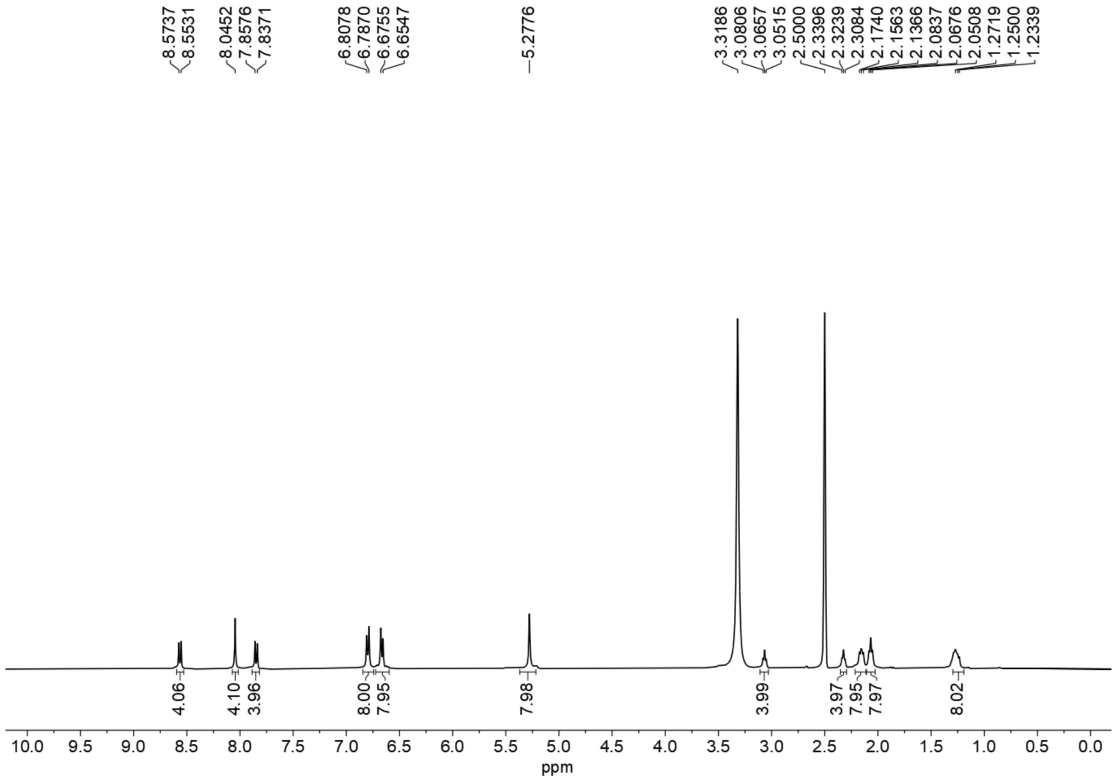

Figure S31. ^{1}H NMR (400 MHz, DMSO-d_6, 298 K) of **CAT**.

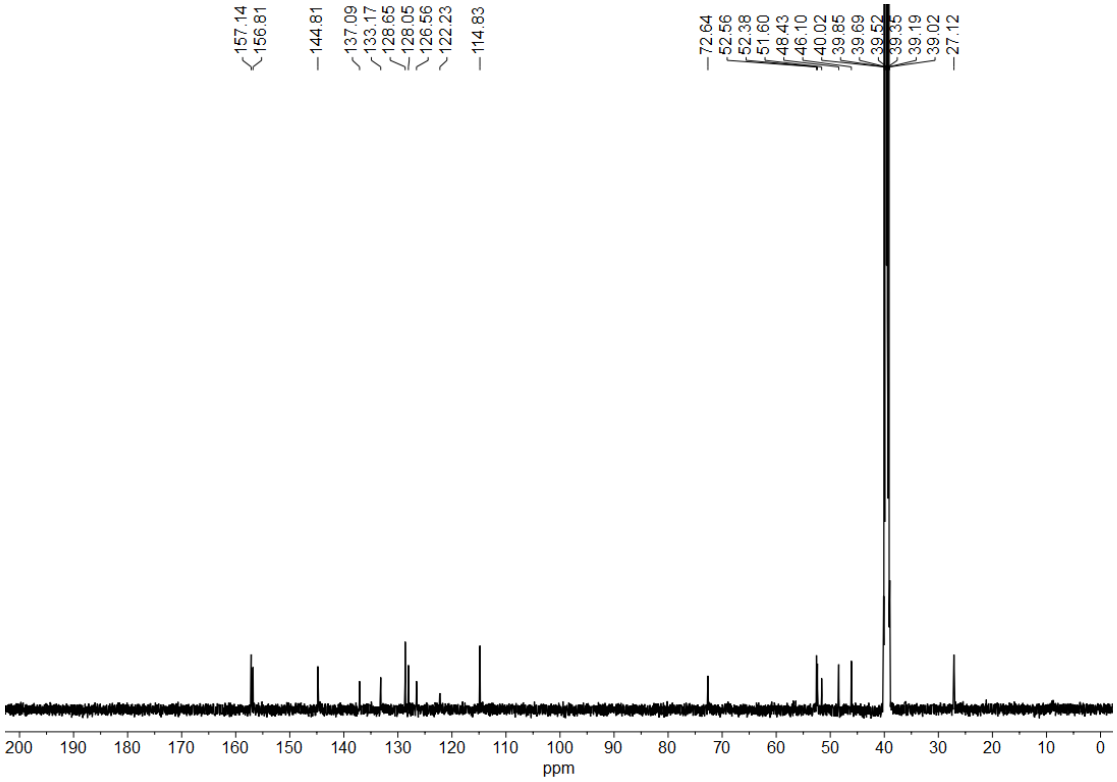

Figure S32. $^{13}C\{^{1}H\}$ NMR (126 MHz, DMSO-d_6, 298 K) of **CAT**.

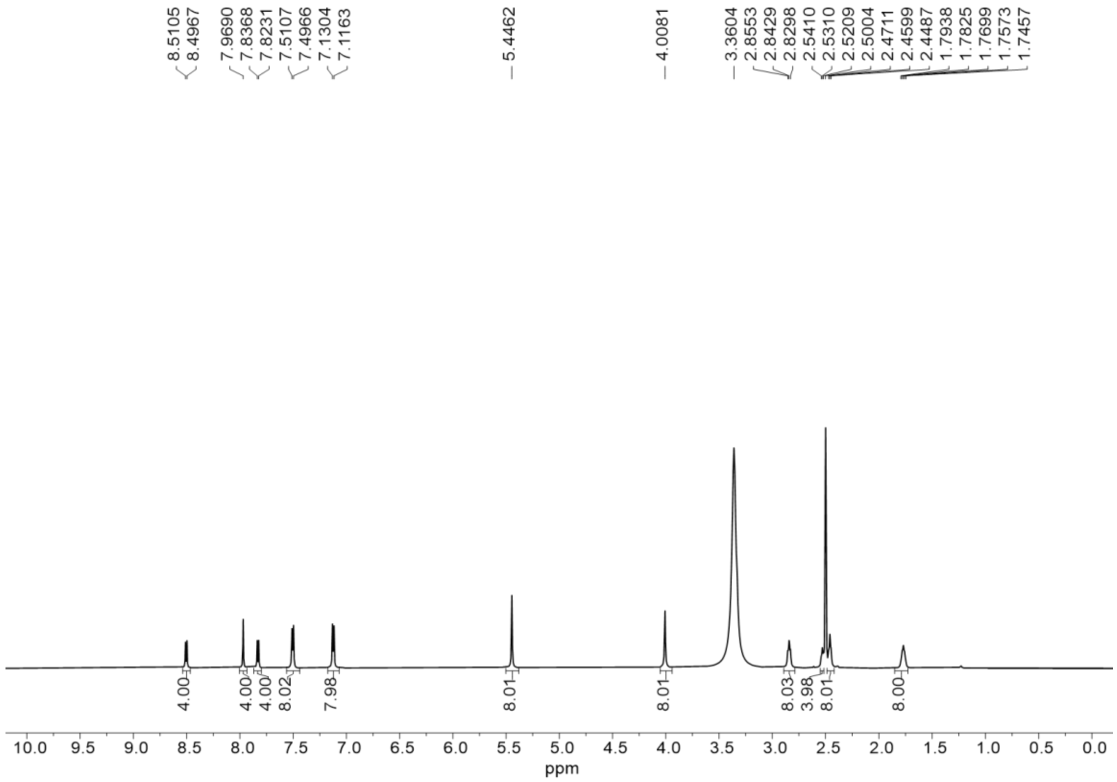

Figure S33. ^{1}H NMR (600 MHz, DMSO-d_6, 298 K) of **MAC**.

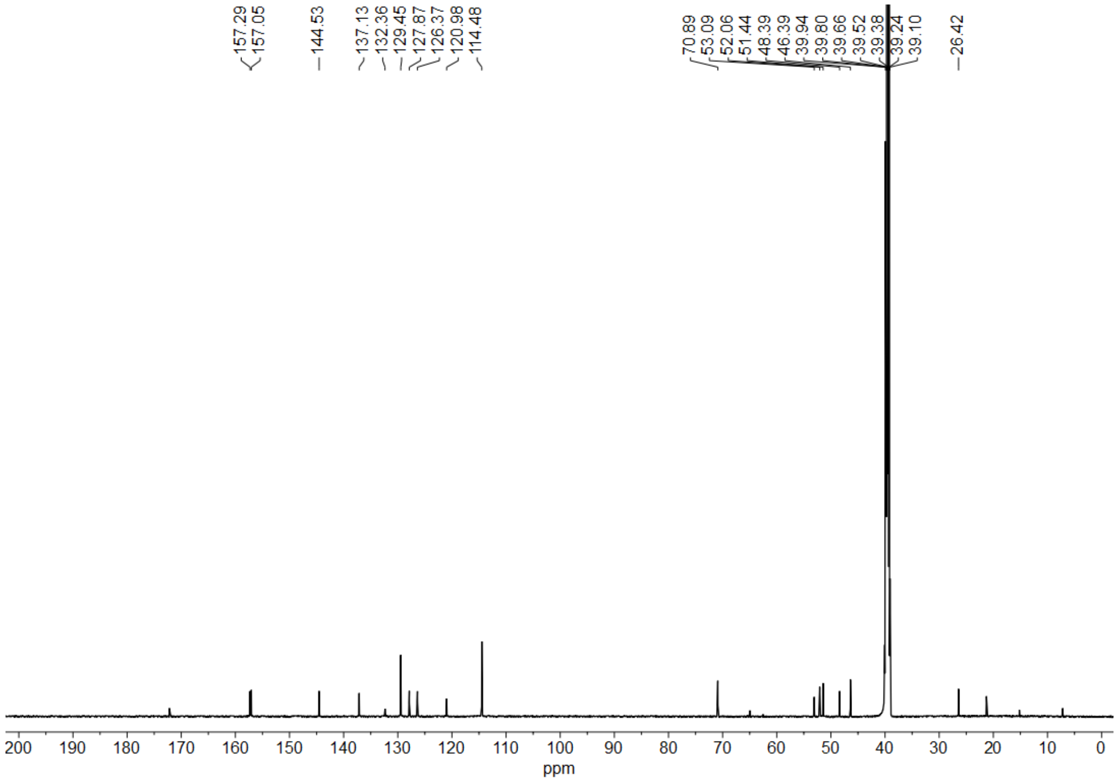

Figure S34. $^{13}C\{^{1}H\}$ NMR (151 MHz, DMSO-d_6, 298 K) of **MAC**.

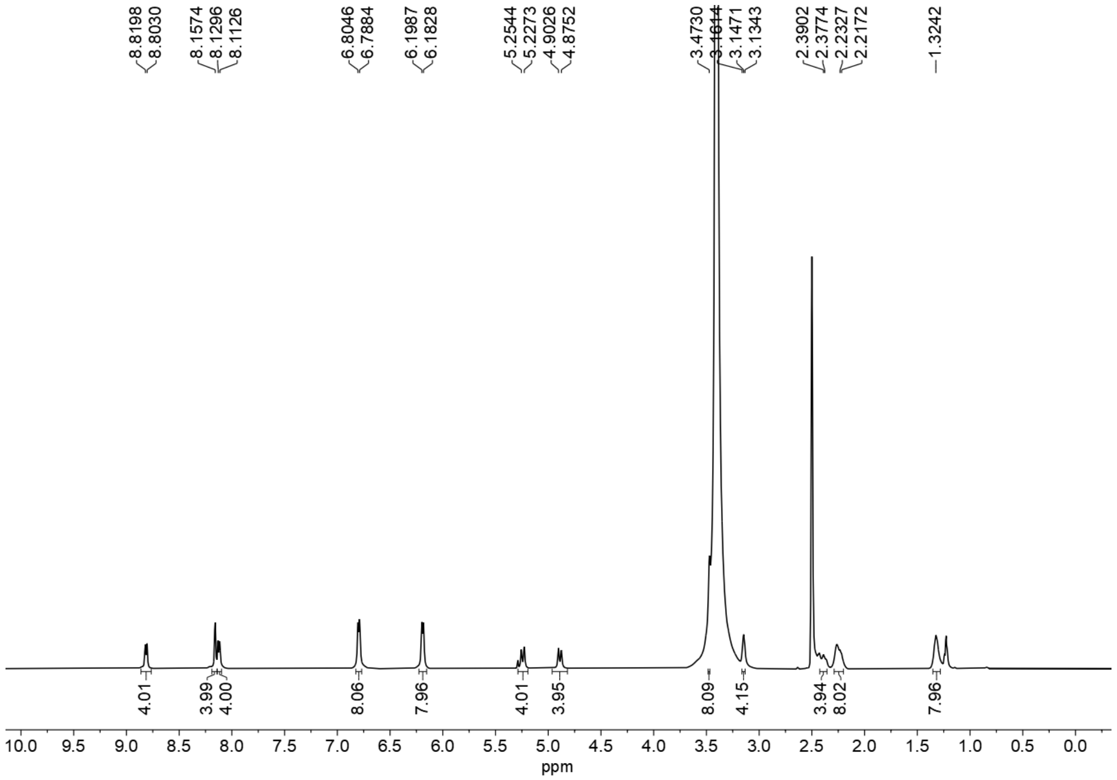

Figure S35. ^{1}H NMR (500 MHz, DMSO-d_6, 298 K) of **Cu-MAC**.

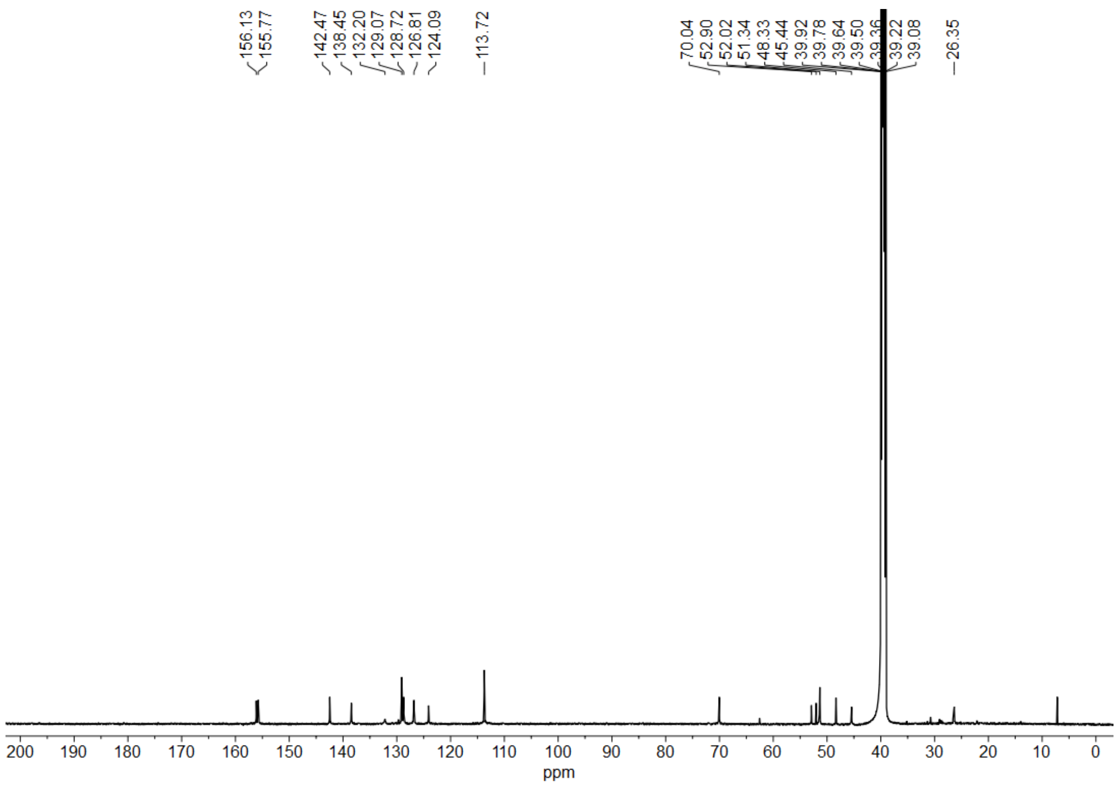

Figure S36. $^{13}C\{^{1}H\}$ NMR (151 MHz, DMSO-d_6, 298 K) of **Cu-MAC**.

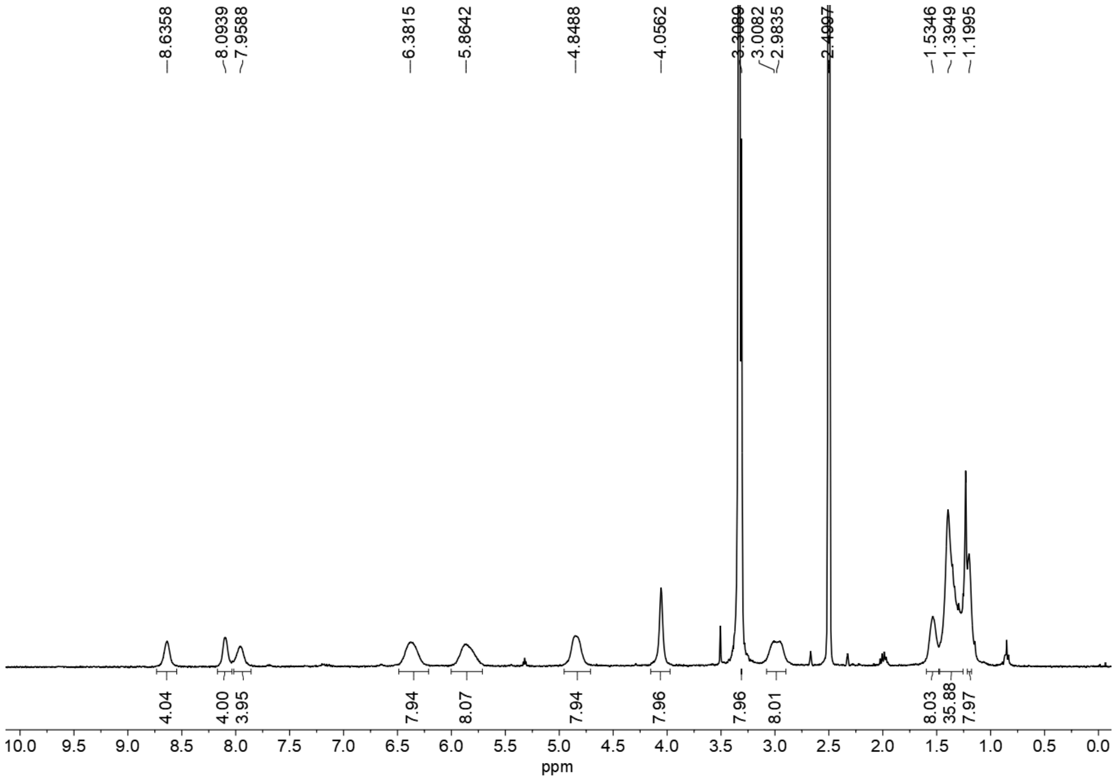

Figure S37. ^{1}H NMR (400 MHz, DMSO-d_6, 298 K) of **Cu-(CAT-Boc)**.

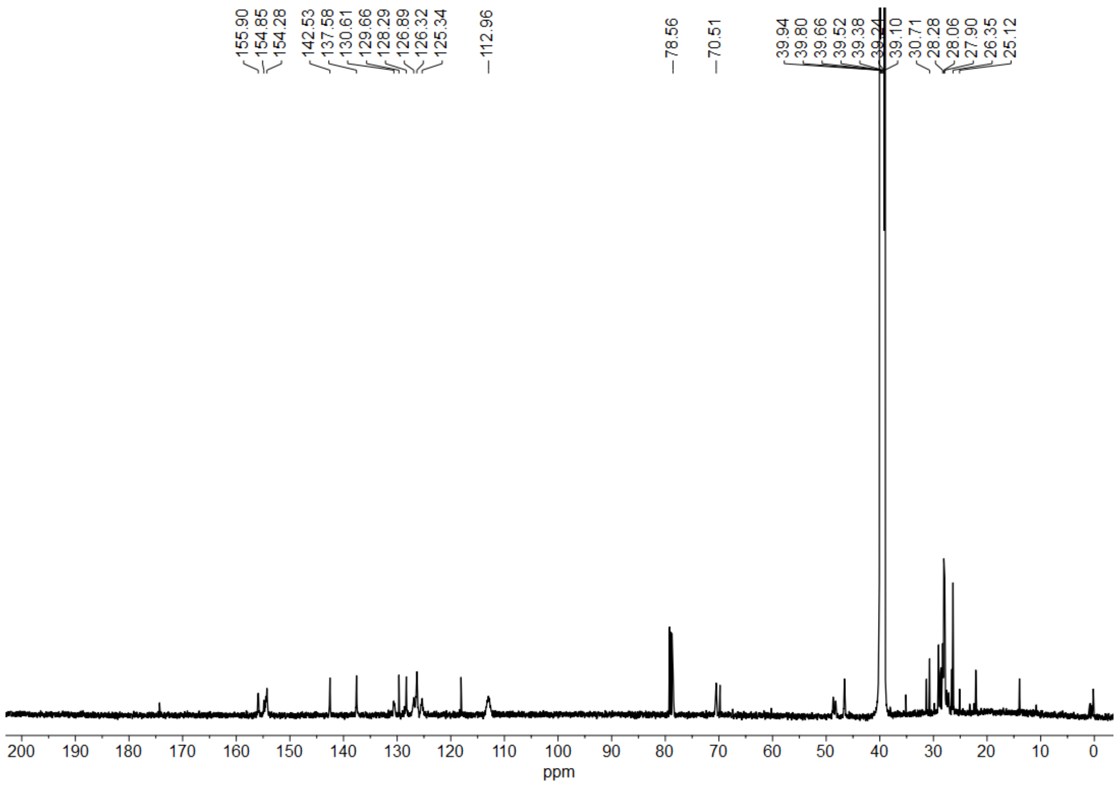

Figure S38. $^{13}C\{^{1}H\}$ NMR (151 MHz, DMSO-d_6, 298 K) of **Cu-(CAT-Boc)**.

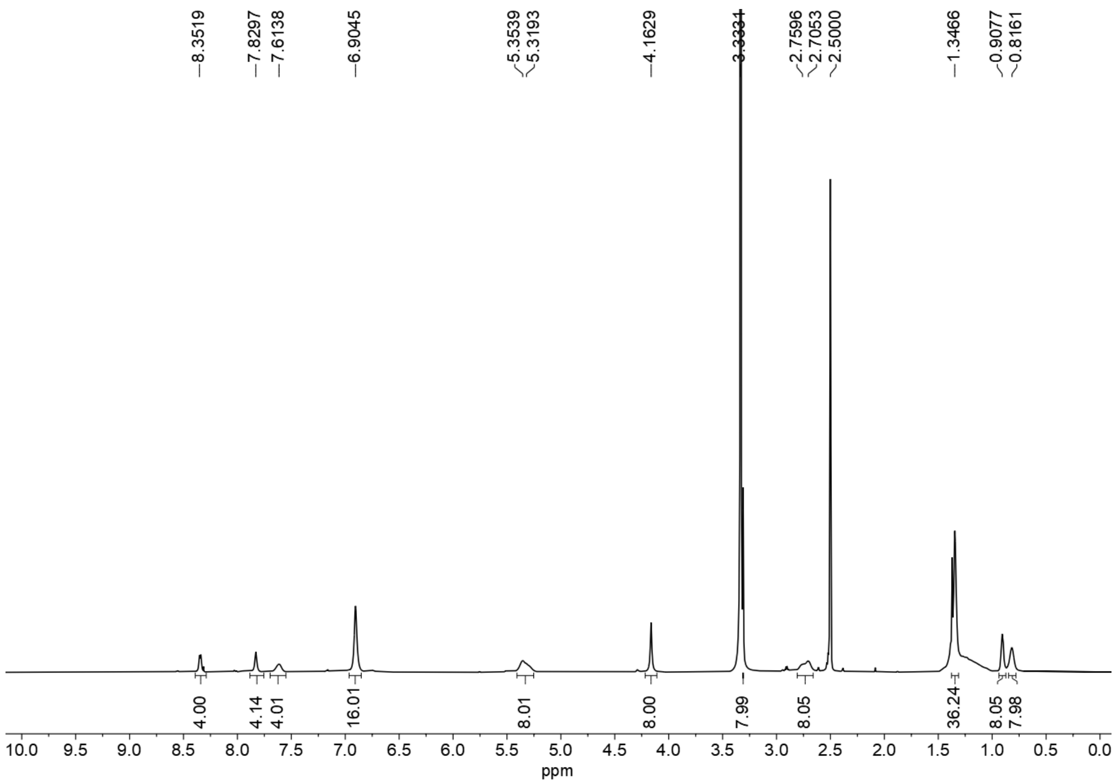

Figure S39. ^{1}H NMR (500 MHz, DMSO-d_6, 298 K) of **CAT-Boc**.

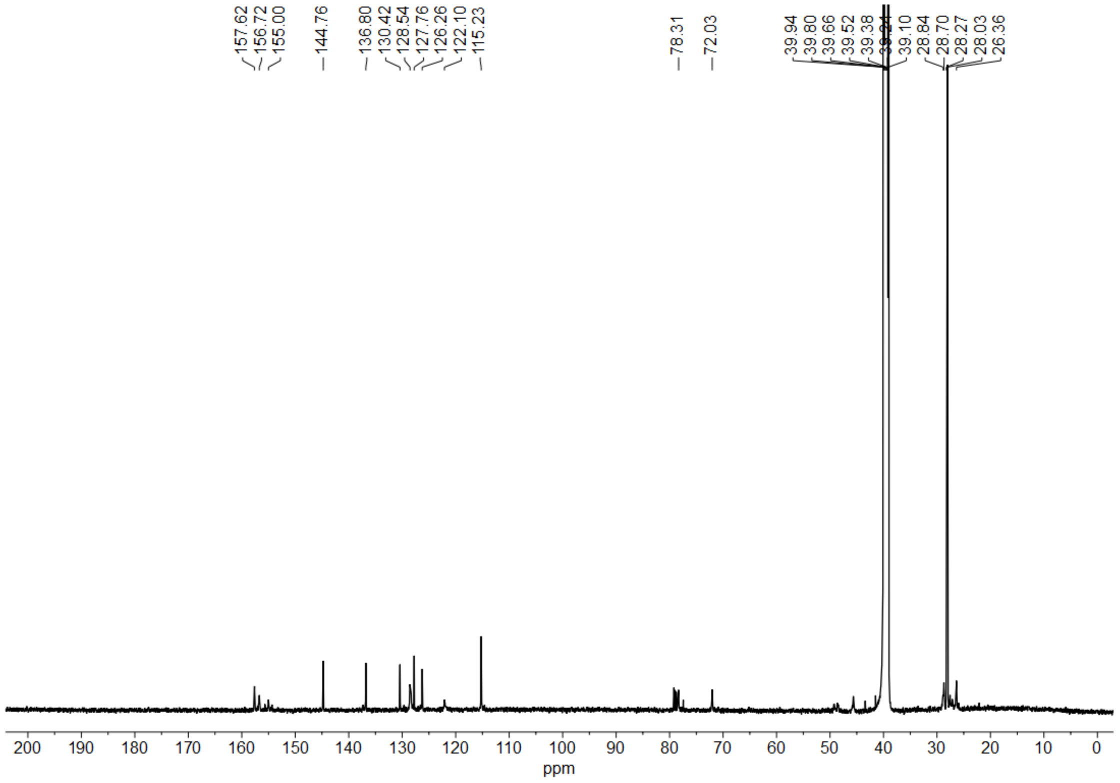

Figure S40. $^{13}C\{^{1}H\}$ NMR (151 MHz, DMSO-d_6, 298 K) of **CAT-Boc**.

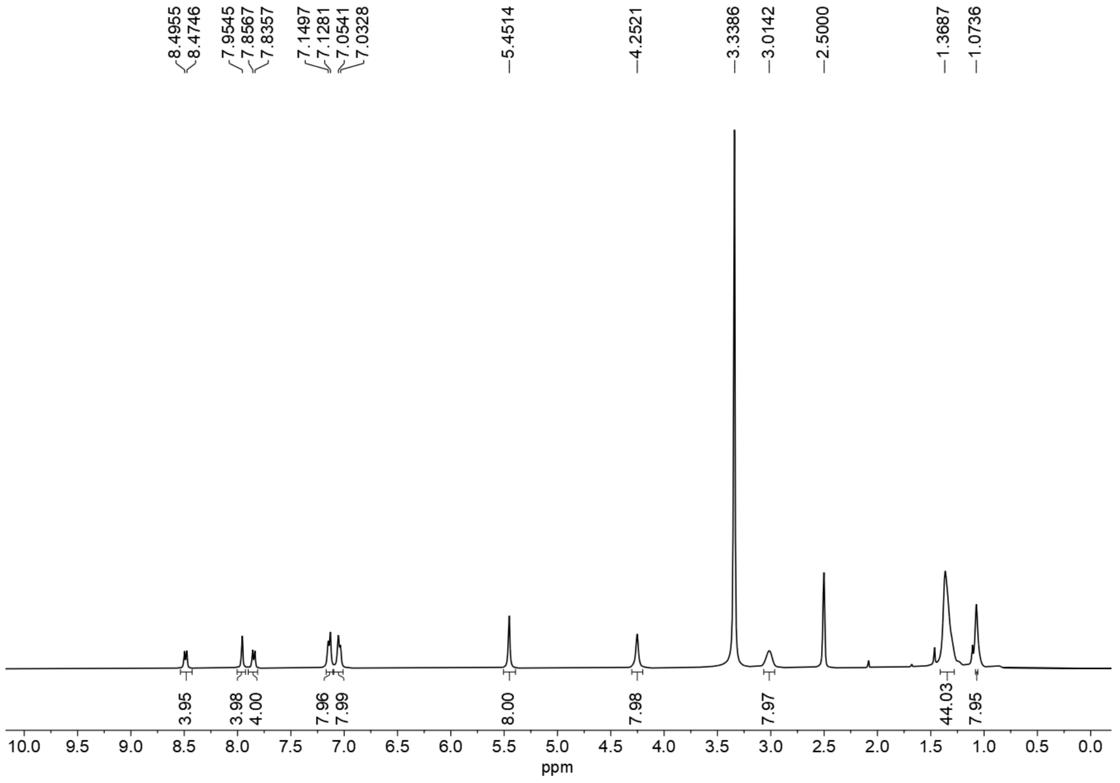

Figure S41. ^{1}H NMR (400 MHz, DMSO-d_6, 298 K) of **MAC-Boc**.

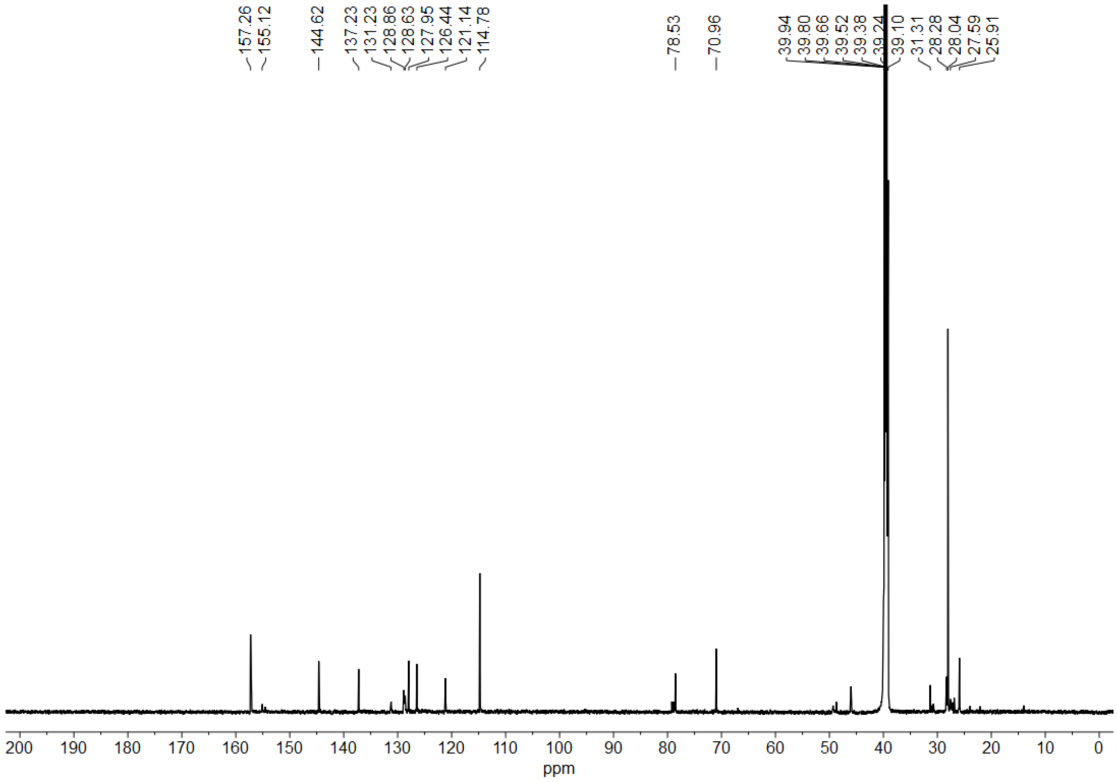

Figure S42. $^{13}C\{^{1}H\}$ NMR (151 MHz, DMSO-d_6, 298 K) of **MAC-Boc**.

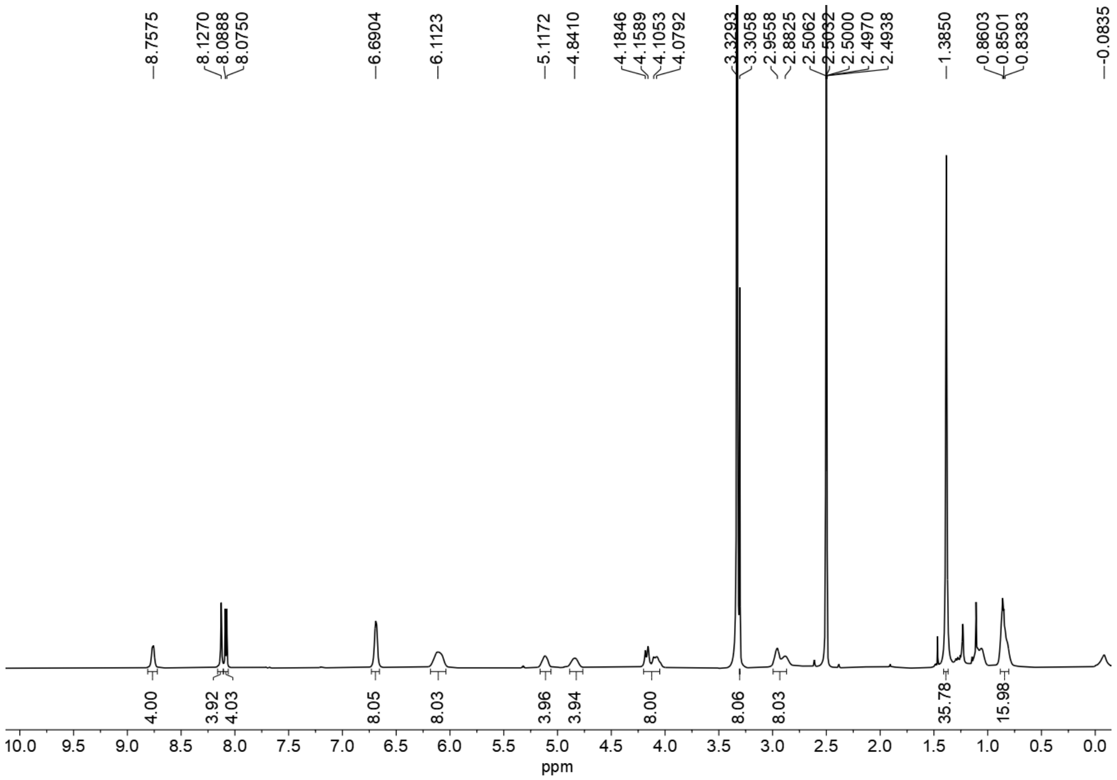

Figure S43. ^{1}H NMR (600 MHz, DMSO-d_6, 298 K) of **Cu-(MAC-Boc)**.

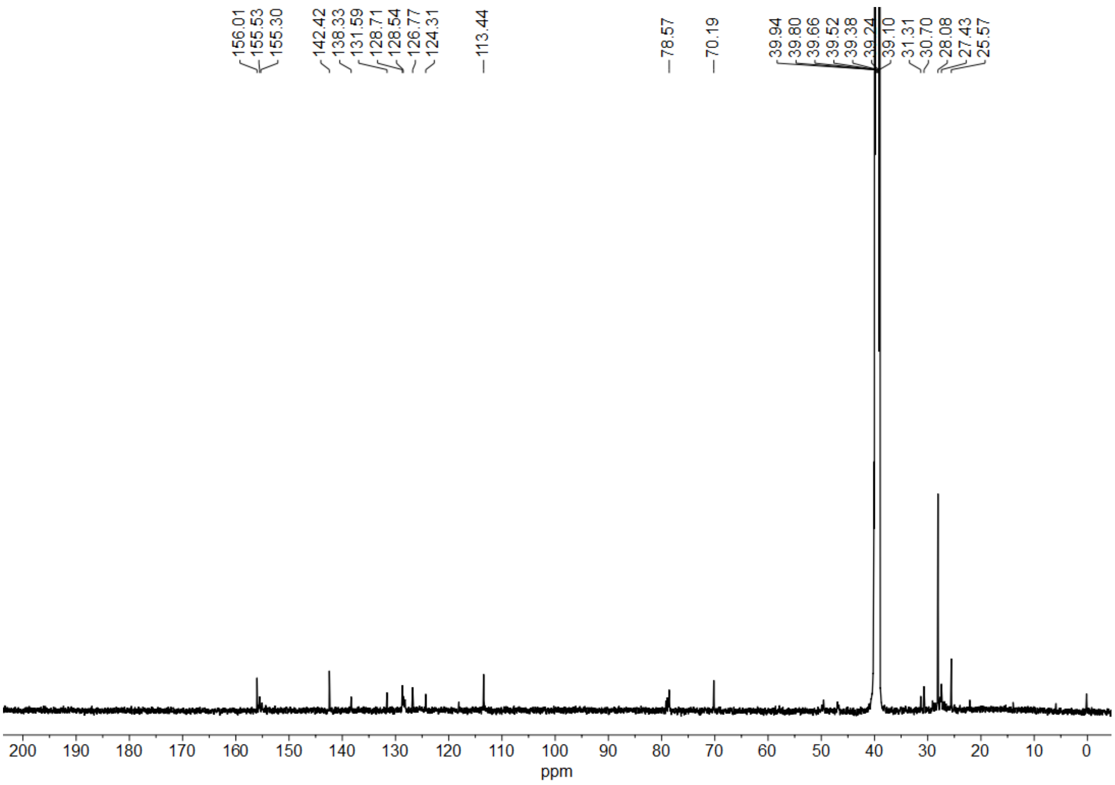

Figure S44. $^{13}C\{^1H\}$ NMR (151 MHz, DMSO-d_6, 298 K) of **Cu-(MAC-Boc)**.

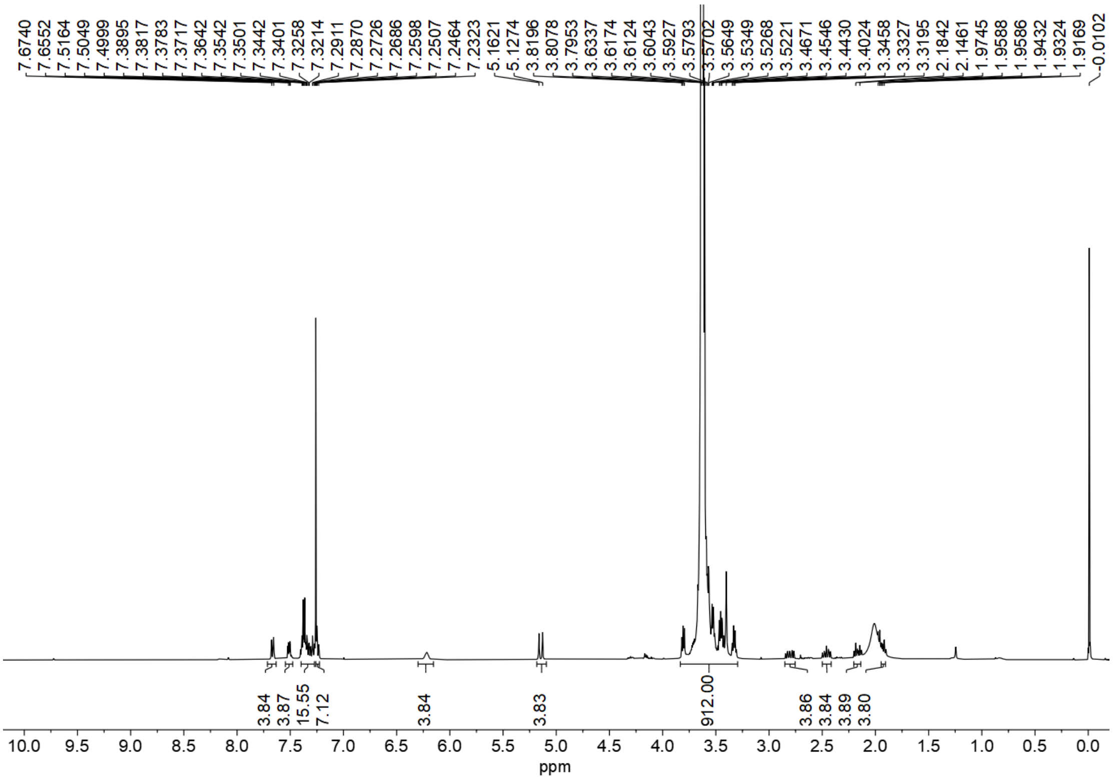

Figure S45. ^1H NMR (400 MHz, $CDCl_3$, 298 K) spectrum of **tetra PEG-DBCO**.

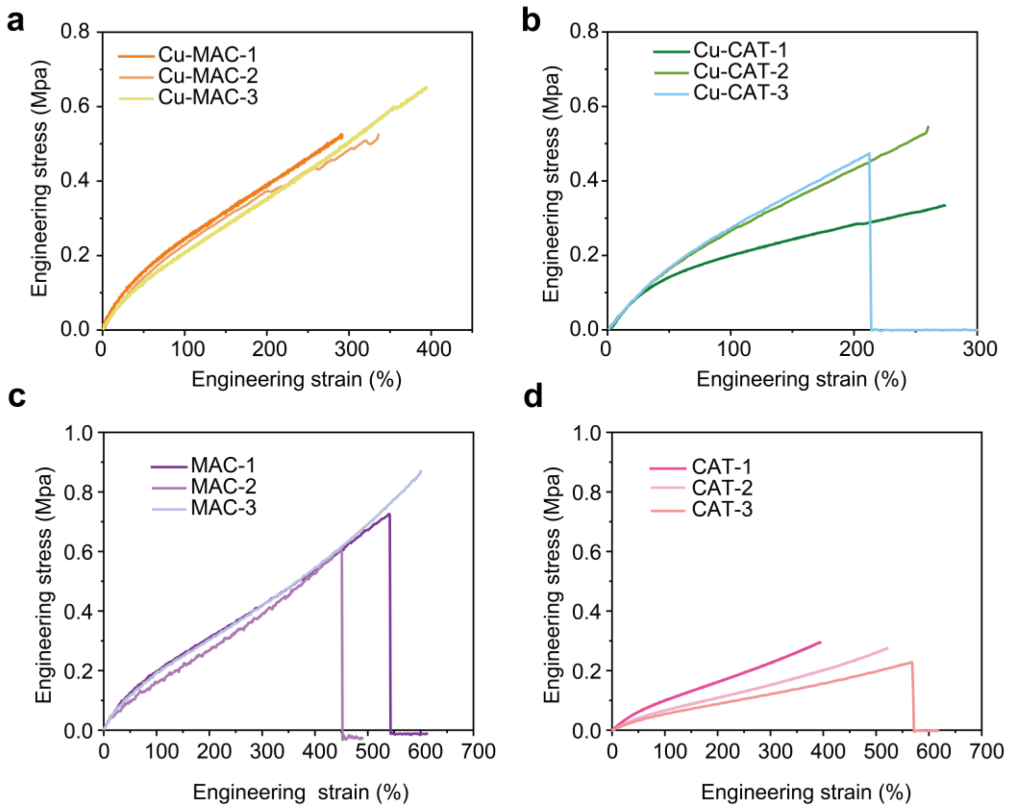

Figure S46. Stress-strain curves of (**a**) Cu-MAC, (**b**) Cu-CAT, (**c**) MAC and (**d**) CAT gels. Each curve represents a different individual sample taken from the same gel.

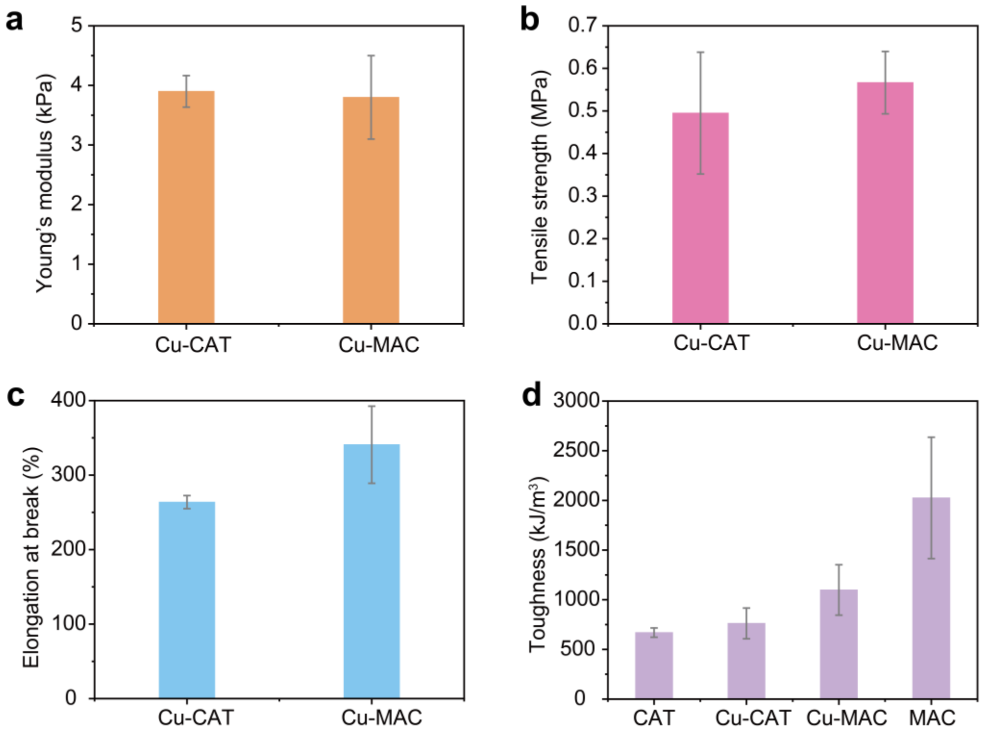

Figure S47. (**a**) Young's modulus, (**b**) Tensile strength, (**c**) Elongation at break of Cu-CAT and Cu-MAC gels. (**d**) Toughness of different polymer gels.

5. References

1. Angelos, S.; Yang, Y. W.; Patel, K.; Stoddart, J. F.; Zink, J. I. Ph-Responsive Supramolecular Nanovalves Based on Cucurbit [6] Uril Pseudorotaxanes. *Angew. Chem., Int. Ed.* **2008**, *47*, 2222–2226.
2. Yee, C.-C.; Ng, A. W. H.; Au-Yeung, H. Y. Control over the Macrocyclisation Pathway and Product Topology in a Copper-Templated Catenane Synthesis. *Chem. Commun.* **2019**, *55*, 6169–6172.
3. Chen, M.; Gu, Y.; Singh, A.; Zhong, M.; Jordan, A. M.; Biswas, S.; Korley, L. T.; Balazs, A. C.; Johnson, J. A. Living Additive Manufacturing: Transformation of Parent Gels into Diversely Functionalized Daughter Gels Made Possible by Visible Light Photoredox Catalysis. *ACS Cent. Sci.* **2017**, *3*, 124–134.
4. Zhu, L.; Li, J.; Yang, J.; Au-Yeung, H. Y. Cross Dehydrogenative C–O Coupling Catalysed by a Catenane-Coordinated Copper (I). *Chem. Sci.* **2020**, *11*, 13008–13014.
5. Tang, M. P.; Zhu, L.; Deng, Y.; Shi, Y. X.; Kin-Man Lai, S.; Mo, X.; Pang, X. Y.; Liu, C.; Jiang, W.; Tse, E. C. M. Water and Air Stable Copper (I) Complexes of Tetracationic Catenane Ligands for Oxidative C−C Cross-Coupling. *Angew. Chem.* **2024**, *136*, e202405971.
6. Deng, Y.; Lu, Z. G.; Lai, S. K. M.; Tang, M. P.; Mo, X.; He, S.; Phillips, D. L.; Tse, E. C. M.; Au-Yeung, H. Y. Structural Dependence of Catenand Effect: Thermodynamic and Kinetic Modulation of Catenane Coordination Properties Via Ring-Size and Exocyclic Substituent Variation. *Angew. Chem., Int. Ed.* **2025**, *64*, e202514599.
